# The Early Origin of the Antarctic Marine Fauna and Its Evolutionary Implications

**DOI:** 10.1371/journal.pone.0114743

**Published:** 2014-12-10

**Authors:** J. Alistair Crame, Alan G. Beu, Jon R. Ineson, Jane E. Francis, Rowan J. Whittle, Vanessa C. Bowman

**Affiliations:** 1 British Antarctic Survey, Natural Environment Research Council, Cambridge, United Kingdom; 2 GNS Science, Lower Hutt, New Zealand; 3 Geological Survey of Denmark and Greenland, Copenhagen, Denmark; Wesleyan University, United States of America

## Abstract

The extensive Late Cretaceous – Early Paleogene sedimentary succession of Seymour Island, N.E. Antarctic Peninsula offers an unparalleled opportunity to examine the evolutionary origins of a modern polar marine fauna. Some 38 modern Southern Ocean molluscan genera (26 gastropods and 12 bivalves), representing approximately 18% of the total modern benthic molluscan fauna, can now be traced back through at least part of this sequence. As noted elsewhere in the world, the balance of the molluscan fauna changes sharply across the Cretaceous – Paleogene (K/Pg) boundary, with gastropods subsequently becoming more diverse than bivalves. A major reason for this is a significant radiation of the Neogastropoda, which today forms one of the most diverse clades in the sea. Buccinoidea is the dominant neogastropod superfamily in both the Paleocene Sobral Formation (SF) (56% of neogastropod genera) and Early - Middle Eocene La Meseta Formation (LMF) (47%), with the Conoidea (25%) being prominent for the first time in the latter. This radiation of Neogastropoda is linked to a significant pulse of global warming that reached at least 65°S, and terminates abruptly in the upper LMF in an extinction event that most likely heralds the onset of global cooling. It is also possible that the marked Early Paleogene expansion of neogastropods in Antarctica is in part due to a global increase in rates of origination following the K/Pg mass extinction event. The radiation of this and other clades at ∼65°S indicates that Antarctica was not necessarily an evolutionary refugium, or sink, in the Early – Middle Eocene. Evolutionary source – sink dynamics may have been significantly different between the Paleogene greenhouse and Neogene icehouse worlds.

## Introduction

**Figure 1:**
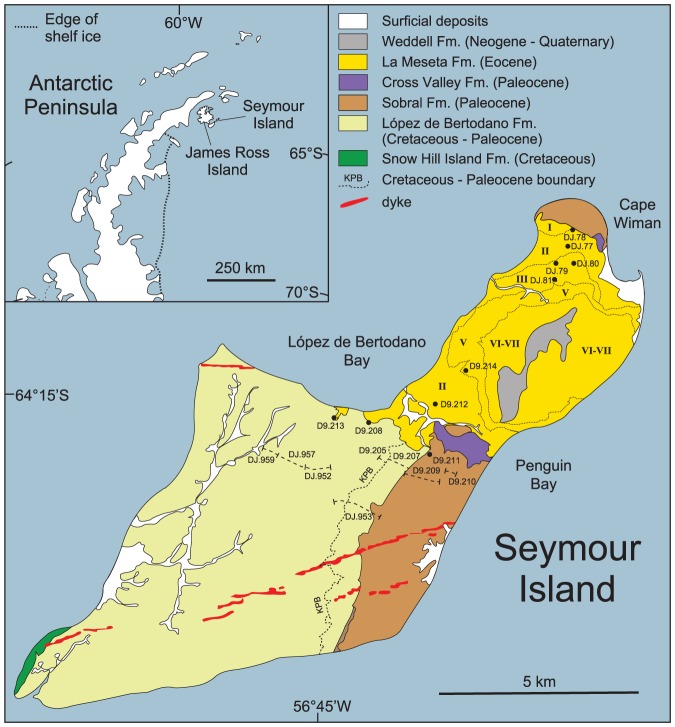
Geological and locality map for Seymour Island, north-eastern Antarctic Peninsula. Roman numerals within the La Meseta Formation indicate Telms 1–7; Telm 4 is a very thin unit that can be traced intermittently along the boundary between Telms 3 and 5. KPB =  Cretaceous/Paleogene boundary. Further details on stratigraphic nomenclature are contained in the text and S1 Appendix. Based on Montes et al [117], with modifications. Cross Valley lies between López de Bertodano Bay and Penguin Bay.

A series of recent studies has clarified that a number of key components of the modern Antarctic marine fauna were in place well before the onset of global cooling in the late Middle Eocene. This is particularly so within the marine invertebrates where significant elements of the numerically dominant Mollusca occur in the fossil record as much as 20 m.y. before the initial stages of cooling at ∼42 Ma [1], [2]. The critical faunas on which these findings are based are all from Seymour Island at the north-eastern tip of the Antarctic Peninsula (Fig. 1) where excellent vertical and lateral exposure has allowed unprecedented access to Late Cretaceous – Early Paleogene marine faunas and floras at a high paleolatitude (∼65°S) [3]–[7]. Since the initial discovery of these representatives of the modern Antarctic marine fauna, work has continued to refine both their taxonomic affinities and relative biostratigraphical positions. A number of new occurrences of modern Antarctic taxa in the fossil record have been recognised and it is important to place these and all previous records in as accurate a stratigraphical framework as possible. Within this highly fossiliferous locality it should be possible to match faunal trends and patterns directly with changes in paleoclimates and paleoenvironments. Is the introduction of elements of the modern fauna in some way linked to the aftermath of the mass extinction event at the Cretaceous – Paleogene (K/Pg) boundary, or perhaps to paleoclimatic events such as Early – Middle Eocene global warming? Are there elements within the fauna that might help us to determine whether Antarctica acted as an evolutionary source or sink during the Early Cenozoic?

## Materials and Methods

The Upper Cretaceous – Lower Paleogene strata of Seymour Island comprise the stratigraphically highest exposed levels of an extensive sedimentary basin situated on the north-eastern flank of the Antarctic Peninsula [8] (Fig. 1). In total, the sedimentary succession comprises a thickness in excess of 2325 m, although the stratigraphy is complicated by the fact that the two highest formations represent large-scale channel structures that have been cut into older, more regularly bedded units [4], [9]–[11]. The component sediments are overwhelmingly volcaniclastic in origin and dominated by mudstones, siltstones and sandstones deposited in various shallow-marine, shelf-deltaic environments. They are fossiliferous throughout, with particularly dense accumulations forming prominent shell beds (or coquinas) in the mid- to upper levels of the Eocene La Meseta Formation (LMF) [11]. It is important to emphasise that this very thick succession of sediments is by no means continuous, with the upper levels in particular being characterised by a series of unconformity-bounded transgressive – regressive cycles [12]–[14]. Nevertheless, as will be argued below, the overall faunal record is complete enough to allow a detailed analysis of changes in a number of major taxonomic groups between the latest Cretaceous (Maastrichtian) and latest Eocene (i.e. spanning the period between approximately 70–35 Ma). A comprehensive review of the litho-, bio-, and chrono-stratigraphies of the latest Cretaceous – Early Paleogene strata of Seymour Island is contained in S1 Appendix.

The positions of all gastropod and bivalve molluscs were plotted as accurately as possible within the generalised stratigraphic scheme presented in S1 Appendix; all British Antarctic Survey (BAS) field data were determined by abney level and Jacobs staff on a series of measured sections (Fig. 1). Latest Maastrichtian taxa were collected from a series of sections measured in central Seymour Island, with section DJ. 953 crossing the Cretaceous/Paleogene boundary (K/Pg) and recovery interval Kplb 10, and then terminating in the base of the Sobral Formation (SF) (Fig. 1). The K/Pg and Kplb 10 sections were repeated at D9. 205/207 and then extended vertically through the complete thickness of the SF (D9. 209/210, Fig. 1). Extensive use was also made of the stratigraphic positions of Maastrichtian - Paleocene molluscs described in the taxonomic monographs by Zinsmeister & Macellari [15] and Stilwell et al. [16]. In the latter study specimens were collected from numerous localities in a 70 km^2^ area of south-eastern Seymour Island and then co-located on a single master section using stratigraphic plane analysis [17]. Whilst this is indeed an interesting and innovative technique, it was found in practice that it produced a number of ranges that contrasted significantly with those in section D9. 209/210. These conflicts are outlined in more detail in the taxonomic appendix (S2 Appendix) where additional Maastrichtian – Paleocene records were added from all published sources, plus the collections of the Paleontological Research Institution (PRI), Ithaca, New York.

The Eocene LMF was investigated using transects at both the northern and south-western ends of the area of outcrop (Fig. 1). In the former of these regions the transect stretches from locality DJ. 78 in informal mapping unit Telm 1, through DJ. 77 (Telms 2 & 3) to DJ. 79 & 80 (Telm 3), and finally DJ. 81 (Telm 4). In the latter, Telm 1 was investigated at D9. 208 & 213, Telm 2 at D9. 212, and Telm 5 at D9. 214 (Fig. 1). In addition, extensive use was made of reference sections S1-2/LM86-1 on the western flank of the LMF, and D-8 on the northern flank in the taxonomic monograph published by Stilwell & Zinsmeister ([18], p. 23 & fig. 39). In this work the stratigraphic occurrences at a large number of localities were projected onto the sections and the results presented as four composite range charts([18], figs 40–43). As Stilwell & Zinsmeister ([18], p. 23) pointed out, it is not a straightforward task to combine sections from different localities into a single reference scheme for the whole LMF. This is because of the marked lenticular nature of the Telms and some significant NNE – SSW variations in thickness across the area of outcrop. In this study a single LMF reference section was established using the stratigraphic scheme presented in Casadio et al. ([19], fig, 2). In some cases it was then possible to plot stratigraphic positions and ranges directly onto it with reasonable accuracy, but in others only relative positions such as “mid-point in Telm” or “upper one-third of Telm” could be obtained. Further details of the ranges used and specimens examined for each taxon are given in the S2 Appendix, which includes a review of all relevant published sources, and the collections of the PRI. Because the biostratigraphical ranges used in this study were established by a variety of different means, and different research groups, it was not possible to fit confidence intervals to them [20].

The numbers of all specimens examined are catalogued in S2 Appendix, where reference is also made to the exact coordinates of each locality. The specimens collected in the 2009–10 field season were obtained under permit from the Foreign & Commonwealth Office, United Kingdom of Great Britain and Northern Island. Permit No. S6-5/2009 was granted under Section 6 of the Antarctic Act 1994 to the Director of the British Antarctic Survey, who then delegated authority for the material to be obtained by the authors. Specimens from earlier field seasons were collected under similar regulations. All UK specimens collected from Antarctica are contained within the reference collections of the British Antarctic Survey, High Cross Madingley Road, Cambridge CB3 0ET, UK, and may be accessed via the NERC Polar Data Centre (http://pdc.nerc.ac.uk). Seymour Island specimens held within the Paleontological Research Institution, Ithaca, New York, the Smithsonian Institution, Washington, D.C., and the collections of GNS Science, Lower Hutt, New Zealand were also examined.

## Results

**Figure 2:**
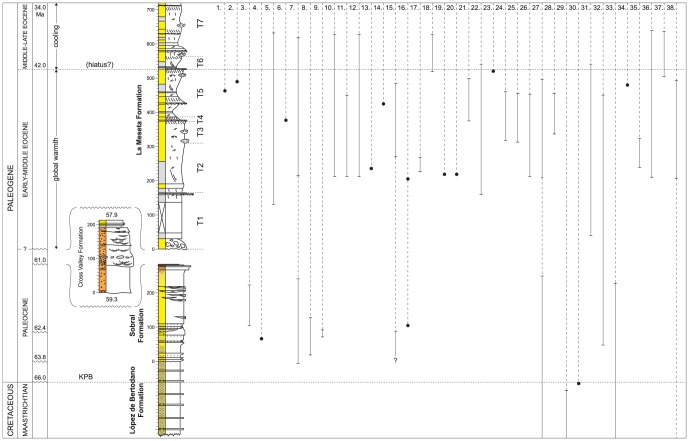
The fossil record of 38 modern Southern Ocean molluscan genera. Solid lines and dots depict actual fossil occurrences and ranges of 38 molluscan genera (1–26 =  gastropods, 27–38 =  bivalves). Full details as to how the occurrences and ranges were established within the stratigraphic framework are contained within the text and S2 Appendix. Klb 9 represents the topmost Maastrichtian stratigraphic unit of the LBF, KPB =  Cretaceous/Paleogene boundary, and (10) is Kplb 10, the recovery interval and topmost informal stratigraphic unit of the LBF. T1–7 =  Telms 1–7 of the LMF; ages (in Ma) are approximate and taken from Montes et al. [117] for the SF, CVF and topmost LMF (Telms 6–7). Further details on the age of the La Meseta Formation are given in S1 Appendix. Gastropods arranged in taxonomic order according to Bouchet & Rocroi [118]; bivalves according to Bouchet & Rocroi [119] and Taylor et al. [120]. 1.*Calliotropis*; 2. *Collonia*; 3. *Euspira*?; 4. *Amauropsis*?; 5. *Falsilunatia*; 6. *Sinuber*; 7. *Perissodonta*; 8. *Probuccinum*?; 9. cf. *Germonea*; 10. *Prosipho*; 11. *Pareuthria*; 12. *Chlanidota*; 13. *Trophon*; 14. *Fulgurofusus*; 15. *Volutomitra*?; 16. *Miomelon*?; 17. *Tractolira*; 18. *Aforia*?; 19. *Spirotropis*?; 20. *Typhlomangelia*; 21. *Epitonium*; 22. *Acirsa*; 23. *Acteon*; 24. *Neoacteonina*; 25. *Tornatellaea*; 26. *Kaitoa*; 27. *Leionucula*; 28. *Yoldia (Aequiyoldia)*; 29. *Limopsis*; 30. *Limatula*; 31. *Cyclocardia*; 32. *Parathyasira*; 33. *Conchocele*; 34. *Mysella*; 35. *Gaimardia*; 36. *Cyamiomactra*; 37. *Hiatella*; 38. *Thracia*.

Thirty-eight modern Southern Ocean molluscan genera are represented in the latest Cretaceous – early Paleogene fossil record of Seymour Island; these comprise 26 gastropods and 12 bivalves. In total these represent approximately 18% of the total modern fauna, 19% of the gastropod genera and 17% of the bivalves (http://www.biodiversity.aq). When traced back through the composite reference section, it is apparent that only five of the 38 genera (13%) extend across the K/Pg, and these are all bivalves: *Leionucula, Limopsis, Limatula, Conchocele* and *Thracia* (Fig. 2) (S2 Appendix). With the probable exception of *Conchocele*, all of these taxa are known to have extensive Late Cretaceous and earlier fossil records (Paleobiology Database; http://paleobiodb.org): *Limopsis* is now known to have an Early Cretaceous (Albian) origin [21], [22], *Limatula* can be traced back to at least the Late Jurassic, and *Thracia* to the latest Paleozoic (http://paleobiodb.org). It is likely that the occurrence of *Conchocele* in the earliest Maastrichtian of the Snow Hill Island Formation, James Ross Basin, represents the first global fossil record for the genus [23], [24] (S2 Appendix).

Therefore it is possible to draw at least a preliminary conclusion that the modern Antarctic bivalve fauna has deeper temporal roots than the gastropod one, and this can be strengthened by the fossil record of at least two other Southern Ocean genera. Although *Astarte* does not occur in the Seymour Island section, it can clearly be traced back in the global fossil record and shown to be widespread in its distribution through the greater part of the Mesozoic era (http://paleobiodb.org). Similarly, the lucinid *Lucinoma* occurs in the subantarctic regions at the present day and can be traced back as far as the Early Cretaceous in Japan [25] and latest Cretaceous in Denmark [26]. In comparison, it is not possible to trace any of the modern Antarctic gastropod genera back to the Cretaceous (Fig. 2). The one possible exception to this rule might be the struthiolariid, *Perissodonta*, which has an extensive Paleocene – Eocene record on Seymour Island (#7, Fig. 2). In a review of the phylogeny of the Struthiolariidae, Zinsmeister & Camacho [27] suggested that the Late Cretaceous New Zealand genus *Conchothyra* could be stem to the entire lineage, but it has to be stressed that no specimens of *Conchothyra* have yet been reported from Antarctica (S2 Appendix).

Within the uppermost levels of the Maastrichtian part of the López de Bertodano Formation (LBF) (Units Klb 6–9, i.e. the uppermost 450 m) there are approximately 19 genera/23 species of gastropods and 28 genera/33 species of bivalves ([28]–[31]; faunal list in S1 Appendix.). In addition it is apparent that within this interval bivalves are very much more abundant than gastropods, with estimates from various reference collections being in the region of 2∶1. Such a taxonomic structure for the latest Cretaceous Antarctic molluscan fauna is similar to that known from the very well studied U.S. Gulf Coast region. An analysis of the Late Maastrichtian Owl Creek, Prairie Bluff, Providence and Severn Formations combined produced the following results: 116 genera/207 species of gastropods, and 104 genera/206 species of bivalves. Some 97,000+ individual specimens of gastropods and bivalves have been examined from these four formations and they reveal a ratio of gastropods to bivalves of approximately 1∶4 [32]–[38]. In other words, although the absolute numbers are much higher on the Gulf Coast (it is, after all, a much bigger area), the relative proportion of gastropods to bivalves in terms of genera or species is very similar, but in terms of numbers of individuals is somewhat higher than in Antarctica. Relative parity in numbers of taxa, and in particular number of species, would appear to be a reasonably consistent feature of Late Cretaceous benthic molluscan assemblages per se [35], [39], [40].

In Antarctica the K/Pg is followed immediately by a 55–60 m thick recovery interval which is informally known as unit Kplb 10 of the LBF [5], [10]. It contains a genuine low diversity – high abundance fauna with just six gastropod and eight bivalve species (faunal list in S1 Appendix.). This fauna contrasts strongly with that found in the lower levels of the overlying Early Paleocene (Danian) SF, where the relative proportion of gastropods to bivalves changes substantially. Within this formation there are approximately 31 genera/34 species of gastropods and 14 genera/18 species of bivalves (Table 1; S2 Appendix); this increase in the relative proportion of gastropods to bivalves from the Maastrichtian to the Paleocene is statistically significant at both genus and species levels (exact binomial tests, P<0.003). Similarly, an analysis of the Danian Kincaid, Aquia and Clayton formations from the Gulf Coast yields a value of 1.4∶1 at the species level, the Danian Wangaloa Formation of New Zealand ∼3∶1, Middle Paleocene faunas of Europe and Greenland between 2.3∶1 and 2.5∶1, and Early – Middle and Middle Paleocene faunas of the Gulf Coast (Wills Point, Porters Creek and Naheola Formations) as high as 4∶1 (using references cited in ([2], S1 Appendix). Analyses of patterns of relative abundance are less precise in the Paleocene but there is a general impression from all regions of a steep increase in the number of gastropod individuals too.

**Table 1:** Numbers of gastropods and bivalves in the three stratigraphic intervals investigated.

	gastropod genera	gastropod species	bivalve genera	bivalve species	neogastropod genera	neogastropod species
**Early - Middle Eocene** (Telms 2–5, LMF)	63	100	37	49	33	52 (16.88)*
**Paleocene** (SF)	31	34	14	18	16	16 (11.02)*
**Maastrichtian** (LBF,Units Klb 6–9)	19	23	28	33	8	8 (7.95)*

Owing to the fact that they were collected over a number of years and by different groups it was not possible to sample-standardise the general collections of gastropods and bivalves with any degree of accuracy. However, the neogastropod collections are better constrained and reasonably accurate estimates of numbers of individuals can be made for all species. Therefore it is possible to use rarefaction to at least partially sample-standardise for the fact that the three time bins are of unequal stratigraphic thickness and temporal duration. * represents values for rarefaction to 100 individuals, i.e. E(S_100_). Total numbers of neogstropods for the three time bins are: Maastrichtian  = 344; Paleocene  = 343; Early - Middle Eocene  = 3483. Key: LBF =  López de Bertodano Formation; SF =  Sobral Formation; LMF =  La Meseta Formation.

In the Early Paleocene gastropods take over from bivalves globally as the dominant element in benthic molluscan assemblages and this is due very largely, but not exclusively, to the rise of one very large clade, the Neogastropoda [40]–[43]. To give some idea of the scale of this expansion through the Cenozoic the total number of neogastropod species in the terminal Cretaceous Maastrichtian stage globally can be estimated at 220 (http://paleobiodb.org) and those at the present day at ∼26000 [2]. Of course, the number of Maastrichtian taxa could be a significant underestimate (especially of those in the tropics) [44], [45], but nevertheless the scale of this global expansion is impressive and can probably be traced right back to a significant radiation in the Early Danian. Approximately 50% of the total Antarctic gastropod fauna at the species level at this time belongs within the Neogastropoda.

**Figure 3:**
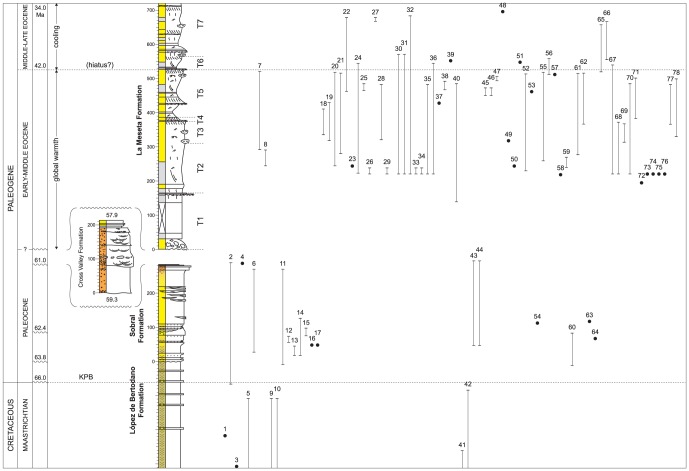
The stratigraphical radiation of the Neogastropoda in Antarctica. Solid lines and dots depict actual fossil occurrences and ranges of 80 neogastropod species. Full details as to how the occurrences and ranges were established within the stratigraphic framework are contained within the text and S2 Appendix. Klb 9 represents the topmost Maastrichtian stratigraphic unit of the LBF, KPB =  Cretaceous/Paleogene boundary, and (10) is Kplb 10, the recovery interval and topmost informal stratigraphic unit of the LBF. T1–7 =  Telms 1–7 of the LMF; ages (in Ma) are approximate and taken from Montes et al. [117] for the SF, CVF and topmost LMF. Further details on the age of the La Meseta Formation are given in S1 Appendix. Gastropods arranged in taxonomic order according to Bouchet & Rocroi [118]. *1.Heteroterma* sp. 2; 2. *Heteroterma* sp.; 3. *Antarctissitys austrodema*; 4. *Pyropsis* sp.; 5. *Taioma charcotiana*; 6. *Taioma sobrali*; 7. *Taioma bicarinata*; 8. *Taioma*? *antarctocarinata*; 9. *“Cassidaria” mirabilis*; 10. Neogastropod, n. gen. B; 11. *Austrosphaera bulloides*; 12. n. gen. *woolfei*; 13. *“Colus” delrioae*; 14. *Probuccinum*? *palaiocostatum*; 15. cf. *Germonea* n. sp.; 16.?*Pseudotylostoma pyrinota*; 17. n. gen.? *polaris*; 18. *“Penion”* n. sp. A; 19. *“Penion”* n. sp. B; 20. *Penion australocapax*; 21. *Prosipho stilwelli*; 22. *Prosipho lawsi*; 23. *Prosipho delli*; 24. *Prosipho polaris*; 25. *Prosipho antarctocosta*; 26. *Prosipho* n. sp. 1; 27. *Prosipho lamesetaensis*; 28. *Pareuthria hookeri*; 29. *Pareuthria* n. sp. 1; 30. *Chlanidota antarctica*; 31. *Chlanidota tuberosa*; 32. *Chlanidota antarctohimaleos*; 33. *Chlanidota*?*antarctohimaleos*; 34. *Chlanidota* n. sp. 1; 35. *Austroficopsis seymourensis*; 36. *Austroficopsis wimani*; 37. *Austroficopsis australis*; 38. *Austroficopsis austrinus*; 39. *Austroficopsis meridionalis*; 40. n. gen. *verrucosa*; 41. Neogastropod, n. gen. A; 42. *Cryptorhytis philippiana*; 43. *Microfulgur binodosa*; 44. *Paleopsephaea*? *nodoprosta*; 45. *Fusinus*? *doylei*; 46. *Microfulgur byrdi*; 47. *Fusinus*? *eonodatus*; 48. *Fusinus*? *suraknisos*; 49. *Fusinus*? *graciloaustralis*; 50. *Trophon radwini*; 51. *Eupleura suroabdita*; 52. Turbinellidae indet.; 53. *Fulgurofusus brecheri*; 54. *Miomelon*? sp.; 55. *Adelomelon fordycei*; 56.?*Adelomelon suropsilos*; 57. *Odontocymbiola amundseni*; 58. *Miomelon antarctica*; 59. *Tractolira* n. sp.; 60. *Volutomitra*? *antarctmella*; 61. *Volutomitra*? *cernohorskyi*; 62. *Volutomitra*? *iredalei*; 63. *Marshallaria*? sp.; 64.?*Cosmasyrinx (Tholitoma) antarctigera*; 65. *Zemacies finlayi*; 66. *Aforia canalomos*; 67. *Marshallaria*? *oliveroi*; 68. *Austrotoma* n. sp.; 69. *Austrotoma*? *ventricosa*; 70. *Austrosullivania lata*; 71. *Austrosullivania striata*; 72. *Gemmula askinae*; 73. *Spirotropis*? n. sp.; 74. *Typhlomangelia*? n. sp.; 75. *Agladrillia*? n. sp.; 76. *Makiyamaia*? n. sp.; 77.? *Splendrillia antarctoliqua*; 78.? *Cochlespira brychiosinus*; 79. *Pristimercia australis*; 80. *Coptostomella*? *notopolaris*. Species 1–10 unassigned to superfamily; 11–49, Buccinoidea; 50–62, Muricoidea; 63–78, Conoidea; 79–80, Cancellarioidea.

In order to track this radiation more closely the stratigraphic ranges of all neogastropods in the Seymour Island section were plotted at the species level (Fig. 3). Of the eight neogastropods known from the latest Maastrichtian, only one of them, *Heteroterma* sp., appears to cross the K/Pg (#2, Fig. 3). However, it should be stressed that this taxonomic category is a broad one and it cannot be determined with certainty that the single specimen occurring ∼8.5 m beneath the K/Pg is precisely equivalent to either that occurring in the mid-levels of the SF, or the four from the topmost levels of the same unit (S2 Appendix). *Heteroterma* is an essentially Maastrichtian – Paleocene genus restricted to the high southern latitudes and eastern Pacific margins (S2 Appendix).

In all other respects, the SF neogastropod fauna of Seymour Island is very different from that of both the latest Cretaceous LBF (units Klb 6–9) and the recovery interval, Kplb 10. The only neogastropods definitely recorded from the latter are a small number of specimens of the buccinoidean *Austrosphaera bulloides* (#11, Fig. 3; S1 Appendix), which occurred in the topmost 6 m of section DJ. 953 (Fig. 1). Thereafter, neogastropods begin to appear in some numbers in the fossiliferous concretionary interval at 48–120 m in the SF (Fig. 3). At least 16 different species have been recorded from this level and they have an altogether much more modern aspect than their latest Cretaceous counterparts. Within the large superfamily Buccinoidea there is at least one species referable to the modern Southern Ocean genus *Probuccinum*, and one very close to the living deep-sea genus *Germonea* (#14, 15, Fig. 3). A third new buccinid, assigned to the modern boreal genus *Colus* by Stilwell et al. [16], is in fact more likely to be a new genus (S2 Appendix), but its presence does serve to reinforce the sudden change to a gastropod fauna of much more modern aspect. In addition there is a volutid very close to the modern genus *Miomelon* from southernmost Patagonia (#54, Fig. 3), and a probable species of *Volutomitra* (#60, Fig. 3). At the genus level there is a statistically significant increase in the proportion of neogastropods in the total gastropod fauna from 28% in the Maastrichtian to 47% in the Paleocene (χ^2^ test, P = 0.011).

The true extent of the stratigraphic gap between the top of the SF and base of the Eocene LMF is unknown but must be at least equivalent to the interval of time represented by the deposition of the CVF (S1 Appendix). In addition it should be stressed that in the stratigraphic column presented here (Fig. 3) the maximum thickness of Telm 1 at the base of the LMF ( = 160 m) is shown, even though it is known to be considerably thinner over much of the basin. But what is apparent is that at a relatively low level in Telm 2 a prolific molluscan fauna is exposed and this can be traced through to the top of Telm 5 (Fig. 3). Whereas the total of 63 gastropod genera recorded from Telms 2–5 represents an approximate doubling in number from the Paleocene SF, the 100 species represents a three-fold increase; this in turn reflects, for the first time, the rise of several species-rich clades (Table 1; S2 Appendix). In comparison, there are only 37 genera of bivalves from the same unit, although it should be pointed out that some of these taxa are still extremely abundant, such as the species of *Cucullaea* and *Retrotapes* that are so prominent in the coquinas of Telm 5 [18]. There is a rise in the proportion of modern gastropod genera between the Paleocene SF (23%) and Early Eocene LMF, Telms 2–5 (33%) but neither this nor a similar comparison between bivalve genera (21% v 24%) is statistically significant (χ^2^ test, P>0.115). However, at the species level there is a significant increase in gastropods from 21% in the SF to 40% in the LMF (P = 0.004) (Fig. 3).

Finally, it is readily apparent that there is a very marked reduction in the number of molluscan species at the boundary between Telms 5 and 6 in the LMF (Fig. 3). The overall reduction in gastropods and bivalves between Telms 2–5 on the one hand and Telms 6 & 7 on the other is 63% (i.e. at the species level); the figure for gastropods alone is 68%, and that for neogastropods is 74%. This is the level at which there is a marked facies change and possible stratigraphical hiatus (S1 Appendix).

## Discussion

### (i) The K/Pg resets the evolutionary stage

Whereas five of the twelve modern bivalve genera having a fossil record on Seymour Island can be traced back to the Cretaceous period, or earlier, none of the 26 gastropods can (Fig. 2). Thus it would appear that the evolution of this key component of the living Antarctic marine fauna [46] is an essentially Cenozoic phenomenon. Study of the global fossil record indicates that mass extinctions are invariably followed by episodes of rapid diversification and biological restructuring [47]–[49], and this would certainly seem to have been the case in the Early Paleocene (Danian) SF. Here, a rapid development of gastropods, particularly at the 48–120 m interval, sees the establishment of a fauna comprising 31 genera, of which five had crossed the K/Pg (but became extinct at a higher level in the sequence) and seven ( = 23%) are modern. The latter comprise the first occurrence of the struthiolariid *Perissodonta*, two naticids (*Amauropsis*?, *Euspira*?) and four neogastropods (*Probuccinum*?, cf. *Germonea, Miomelon*? and *Volutomitra*?) (# 8,9,15,16, Fig. 2). In total, neogastropods comprise 50% (genus or species levels) of the fauna, with by far the largest grouping occurring within the Buccinoidea. This appears to have been the dominant superfamily in the polar regions from the Paleocene onwards [1], [2].

Following the approach adopted in a number of recent studies, we can assign the SF neogastropod taxa to three basic categories: survivors, invaders and locally evolved new taxa [50], [51]. Of the 16 genera, probably only two can be placed in the survivor category, in the sense that they survived the K/Pg extinction event in Antarctica (*Heteroterma, Taioma*; #2,6, Fig. 3); a third possible survivor is *Pyropsis* (#4, Fig. 3), which was widespread in both hemispheres in the Late Cretaceous (http://paleobiodb.org). *Austrosphaera* is a possible immigrant from central Chile [52], and *Paleopsephaea*? from the U.S. Gulf Coast (S2 Appendix). All the other 11 genera ( = 69% of Paleocene neogastropods) show strong southern high-latitude affinities. *Probuccinum*?, cf. *Germonea* and three new genera (“*Colus*”, n. gen. *woolfei* and n. gen.? *polaris*) can be classified as Antarctic taxa, and *Microfulgur*, *Marshallaria*? and?*Cosmasyrinx (Tholitoma)* suggest links with New Zealand.? *Pseudotylostoma* and *Miomelon*? in turn have lnks with Patagonia, and *Volutomitra*? shows more general southern high-latitude affinities (S2 Appendix).

The Paleocene radiation of gastropods continues into the Early - Middle Eocene LMF (S1 Appendix) where there is a two-fold increase in numbers at the genus level, and three-fold at the species level in Telms 2–5 (Table 1, Fig. 3). Of the 40 species assigned to modern Antarctic genera, two are vetigastropods, six struthiolariids (i.e. *Perissodonta*), three naticids, five ptenoglossans, 20 neogastropods, three heterobranchs and one opisthobranch. The total number of neogastropods in this unit (i.e. modern + extinct taxa) is 32 genera (a two-fold increase from the Paleocene) and 52 species (a three-fold increase). The Buccinoidea is again the dominant group (47%) and for the first time the Conoidea (25%) form a prominent element. Gastropods in general, but neogastropods in particular, show a very significant increase in numbers in Antarctica between the K/Pg and approximately late Middle Eocene, and this trend is still apparent after partial sample-standardisation of the three time bins using rarefaction (Table 1, Fig. 3).

### (ii) Diversification in a greenhouse world

It is by now well established that an approximately 20 m.y. interval between the mid-Paleocene and the late Middle Eocene (i.e. ∼42–62 Ma) represents the globally warmest time of the last 75 m.y. [53]. During the acme of this greenhouse world, the Early Eocene Climatic Optimum (EECO) (51–53 Ma), the polar – equatorial thermal gradient was unusually low and tropical rainforests spread to paleolatitudes of 55°–65° N and S, or occasionally even higher [54]–[57]. At the Eureka Sound locality, Ellesmere Island, Canadian Arctic (∼75°N), lush mixed conifer – broadleaved forests inhabited by alligators, turtles and thermophilic mammals suggest summer temperatures of 20°C, or more, and above-freezing winters [58]. Similarly, in Patagonia (∼47°S) a highly prolific Early Eocene leaf flora with tropical affinities indicates winter temperatures warmer than ∼10°C [59], [60].

In the marine realm it has been estimated that during the Early – Middle Eocene the tropics and subtropics were some 40–50% greater in areal extent than at the present day [61]. By the Late Paleocene, equatorial sea surface temperatures (SSTs) were in the region of 20°–26°C, and in the high latitudes approximately 8°–12°C; the overall latitudinal temperature gradient was in the order of half what it is at the present day (i.e. 28°–30°C) [62]. The polar – equatorial temperature contrast was reduced even further in the Early Eocene, with a combination of dinocyst assemblages and archael membrane lipid (TEX_86_) analysis indicating background Arctic SSTs of ∼22°C that rose to as high as 26°–27°C during the EECO [63], [64]. The same drill core samples from the Lomonosov Ridge have also yielded fossil palm pollen [64]. Using both Mg/Ca ratios and δ^18^O values from planktonic foraminifera, and TEX_86_ from bathyal sediments, SSTs of ∼30°C have been obtained from the Canterbury Plains, New Zealand (paleolatitude 55°S) for the interval 50.7–46.5 Ma (with peak values concentrated at 50.7–48.3 Ma) [65]. Even higher values of 34°C have been obtained from TEX_86_ analysis from the East Tasman Sea, S.W. Pacific (paleolatitude 65°S) for ∼53 Ma [66], but it has to be stressed that such values are almost certainly significant over-estimates and biased towards high summer values [67]. Nevertheless, they are testament to a very much reduced latitudinal gradient in SSTs in the Early – Middle Eocene [68], [69], and such gradients are in close agreement with those based on Mean Annual Temperature (MAT) and Coldest Mean Month (CMM) for Early Eocene continental interiors [57], [70].

There is a considerable volume of paleontological evidence to link this 20 m.y. period of extreme global warmth with a major phase of biotic diversification. This was certainly a time of significant radiation of tropical plants [60], [71] and in all probability this in turn facilitated the coeval proliferation of many insect, bird and mammal lineages [72]–[77]. In the marine realm too, there was a very marked increase in the numbers of invertebrate taxa, such as tropical corals, brachyuran crabs and molluscs, as well as many groups of fish [40], [78]–[81].

So, what precisely was the climate of Antarctica in the Early – Middle Eocene? Some exciting evidence from IODP Site U1356, approximately 300 km off the Wilkes Land Coast, East Antarctica (paleolatitude ∼65°S), indicates persistent near-tropical warmth throughout the Early Eocene. A palynological assemblage demonstrates that in the hinterland of the Antarctic continent, at ∼70°S, there were forests with diverse mesothermal to megathermal elements, including both palm and baobab trees [82]. Paratropical conditions in the lowlands of the Wilkes Land margin persisted in the interval 51.9–53.6 Ma but were replaced by a strong expansion of *Nothofagus*-dominated temperate rainforests between ∼46.0–49.3 Ma [82].

In comparison, the paleoclimate record of West Antarctica at approximately equivalent paleolatitudes is somewhat different. A variety of paleontological and geochemical proxies suggest that Late Cretaceous temperatures peaked in the Coniacian – Early Campanian (∼82–89 Ma) with MATs in the region of 16°–20°C on land, and up to 17°C in the Southern Ocean [83]–[86]. Thereafter marine temperatures declined steadily through the Late Campanian, and apart from brief excursions just before the K/Pg, were in the region of 8°C in the Late Maastrichtian – Early Paleocene [86], [87]. A recent comprehensive study of both the Sobral and Cross Valley formations using branched glycerol dialkyl glycerol tetraethers (br GDGTs) in bacterial membrane lipids has yielded continental paleotemperature estimates of 12.4+/−5°C for the latest Maastrichtian – earliest Paleocene and 8.7+/−5°C for the mid- to late Paleocene [88]. A predominantly cool temperate climate during the latest Cretaceous and Paleocene for the Antarctic Peninsula region is supported by paleobotanical data [84] and is consistent with paleofloral and GDGT- derived estimates from New Zealand [88].

Detailed correlation of the Eocene open ocean paleoclimate record with that of the Seymour Island continental margin sequence has always proved problematic. A general impression of the latter, gained from studies to date using a variety of paleoclimatic proxies, is that the LMF has a genuine admixture of both warm- and cool-temperate affinities, and that there is a significant cooling event somewhere near the top of the section [89]–[92]. The most recent and detailed paleotemperature analysis used clumped isotopes from bivalve shells to give temperature estimates in the range of 7°–10°C throughout the LMF. Such values are generally 1°–5°C higher than the previous δ^18^O estimates obtained by Ivany et al. [92], but are in good agreement with TEX_86_ temperatures of 9°–17°C obtained from sediments hosting the bivalve fossils [93]. These Eocene SST values from Seymour Island are on average 7°C lower than TEX_86_ temperatures obtained from a similar paleolatitude in the SW Pacific (East Tasman Plateau) [66]. This and other lines of evidence strongly suggest that there was a large-scale Eocene zonal SST gradient between the South Atlantic and SE Pacific oceans [93]. The most likely cause of this was poleward flow of warm subtropical waters into the Ross Sea region and then net return at depth by thermohaline circulation [93].

The overwhelming affinities of the LMF molluscan fauna are temperate, but it could be argued that, with over 100 species of gastropods and a bivalve fauna rich in cucullaeids, mytilids and a variety of heterodonts, these were predominantly warm-temperate [18]. Micropaleontological evidence suggests that characteristic subtropical fauna and flora appeared at least periodically in the Southern Ocean by the latest Paleocene and reached peak abundance in the Early – Middle Eocene [94], [95]. Further signs of what may well have been distinct pulses of cool- subtropical waters into the Seymour Island region during the Early – Middle Eocene are provided by the regular occurrence of nautiloids in Telms 1–3, and their less frequent appearance in Telms 4–6 [96]; turtles have also been found in Telms 2–5 [97].

Therefore the prominent radiation of neogastropods and other marine taxa on Seymour Island could be linked to a significant rise in SSTs from the Paleocene into the Early – Middle Eocene [88]. The cause of the abrupt reduction in the neogastropod radiation at the Telm 5–6 boundary is currently unknown, but is quite possibly climate-related (S1 Appendix). Superimposed on a general shallow cooling trend that may have initiated as early as early Middle Eocene [53], there is evidence of three distinct climate steps in the Weddell Sea region that are associated with temperature drops of approximately 3°C; these occurred at ∼43, 40 and 36 Ma [98]. The prominent cooling step at ∼41 Ma detected by Ivany et al. [92] within Telm 6 is also seen in the new paleotemperature curve developed by Douglas et al. [93], and there is evidence of an even steeper drop at ∼42 Ma that we have tentatively linked to the Telm 5–6 boundary (S1 Appendix). The position of the last (∼36 Ma) step is less clear but as this is close to the Eocene – Oligocene transition it may not be preserved on Seymour Island. It is becoming steadily more apparent that global climates deteriorated substantially well before the Eocene – Oligocene boundary, and a variety of evidence points to fluctuating volumes of ice on the Antarctic continent by ∼42 Ma [99].

### (iii) Implications for global biodiversity models

The marked increase in gastropods in general, and neogastropods in particular, between the mid-Paleocene (∼63 Ma) and late Middle Eocene (?42 Ma) in the Seymour Island section (Table 1, Fig. 3) can be interpreted as a significant high-latitude evolutionary radiation. This is particularly so when it is considered that 33% of all gastropod genera and 40% of all species within Telms 2–5 of the LMF can be linked to modern Southern Ocean taxa. There are at least 52 neogastropod species in this interval and 40% of these belong within modern Antarctic genera. It is important to emphasise that other taxonomic groups radiated substantially in Antarctica at this time too. One of the most important examples to emerge to date is that of fish, where some 35 species from 26 families have so far been identified from Telm 4 alone [100]. And at one remarkable locality in Telm 5 no fewer than 14 shark species have been recorded. This would appear to represent a level of taxonomic diversity equivalent to that of the tropical eastern Pacific at the present day [101], [102].

Although qualitative comparisons suggest that the scale of the taxonomic diversity increase in neogastropods was eclipsed by that seen in tropical/subtropical Paleocene – Eocene sections in both the U.S. Gulf Coast and Anglo-Paris basins ([2], [78]; JAC, unpublished observations], it is nevertheless intriguing to consider that the Early Cenozoic evolution of what is today one of the largest clades in the sea [2] may have been marked by a truly global radiation. And this is in turn important because it would indicate that the Antarctic was not necessarily acting as an evolutionary sink at this time for taxa that originated elsewhere. The concept of the tropics acting as an evolutionary source and both polar regions as sinks can be traced back some way in the scientific literature [103]–[105]. It received considerable impetus in the terrestrial realm from the formulation of the principle of Tropical Niche Conservatism (TNC), which simply states that many modern taxonomic groups originated in the formerly much more extensive tropics, with comparatively few spreading subsequently into temperate, high-latitude regions [106], [107]. Such a concept views the tropics as both an evolutionary cradle and a museum, but the poles as essentially just museums [108], [109].

Closely allied to TNC is the “Out of the Tropics” (OTT) hypothesis, which has been much more specifically linked to the marine realm. In this scenario many marine taxa preferentially originate in the tropics and then expand into higher latitudes but without losing their original tropical distributions; over time a distinct latitudinal gradient in taxonomic diversity is built up [110]. Significant empirical support has been obtained for OTT from a comprehensive global dataset of both living and fossil marine bivalves, and this in turn has been used to develop a dynamic evolutionary model that describes diversity, endemism and age in a system containing two distinct regions: the tropics and extra-tropics [111]. This model predicted that, on average, the polar regions would have a lower rate of origination, higher rate of extinction, and higher rate of immigration of marine bivalve genera than tropical/lower latitude regions. Taxonomic diversity in the polar regions predominantly reflects dispersal of taxa that evolved elsewhere rather than in-situ origination – extinction dynamics ([111]; see also [112]).

Clearly, our knowledge of gastropod clade dynamics lags significantly behind that of bivalves and the true biogeographic significance of the Early Cenozoic radiation of neogastropods in Antarctica is largely unknown. In the future it will be important to determine both whether the scale of increase from the Paleocene to Early – Middle Eocene is similar to that of lower-latitude localities, and whether there was significant faunal interchange between high- and low-latitudes during this interval. But the pattern established here offers a rare glimpse of high-latitude evolutionary dynamics in a greenhouse world and suggests that, at least for the neogastropods, Antarctica was not an evolutionary sink in the Early Cenozoic. Perhaps there was a fundamental difference in evolutionary source – sink dynamics between the Early Cenozoic greenhouse and Late Cenozoic icehouse worlds?

Finally, Erwin [113] has raised the intriguing possibility that taxonomic diversity does not necessarily track climate as closely as is often imagined. It may indeed build up during greenhouse intervals but then does not necessarily fall by equivalent amounts when climates deteriorate. In this way greenhouse intervals may act as essential global biodiversity pumps through long intervals of geological time. The relative balance of biodiversification between greenhouse and icehouse states is currently an area of intense investigation [114]–[116].

## Conclusions

- A comprehensive biostratigraphical analysis of the latest Cretaceous – early Paleogene molluscan fossil record of Seymour Island, Antarctica indicates the presence of 38 modern Southern Ocean genera (i.e. 26 gastropods and 12 bivalves). This figure is equivalent to ∼18% of the total modern benthic molluscan fauna.
- Only five of these genera can be traced back over the K/Pg into the Late Maastrichtian, and they are all bivalves. There is paleontological evidence to suggest that these five, and at least two other bivalve genera, have Late Cretaceous or earlier origins.
- The relative balance of benthic molluscan faunas changes immediately after the K/Pg with gastropods taking over as the dominant group; this is a reflection of a global trend. 50% of the species occurring within a prominent radiation of gastropods in the Early Paleocene Sobral Formation can be assigned to the large neogastropod clade; a number of these have a distinctly modern aspect.
- There is a more than three-fold increase in molluscan taxa at the species level between the SF and the essentially Early – Middle Eocene Telms 2–5 of the LMF. 52 species of Neogastropoda occur in this interval, and of these 21 can be assigned to modern Antarctic genera; overall, 40% of the total gastropod fauna has modern Southern Ocean affinities. The Buccinoidea is particularly common and contains a number of species-rich genera.
- The radiation of gastropods in general, and neogastropods in particular, can be linked to a distinct phase of global warming. There is no real evidence of persistent tropical climates within the LMF but it was certainly temperate, and in all probability warm-temperate, for long periods of time. The Early – Middle Eocene greenhouse world seems to have initiated a major phase of global diversification that included the polar regions in both the marine and terrestrial realms.
- A pronounced extinction event at the Telm 5–6 boundary (42 Ma?) may represent the first of three stepwise cooling events recognised in the Middle – Late Eocene deep-sea record of the Weddell Sea.
- It would appear that the roots of the modern Antarctic marine fauna may well lie within the last major phase of global greenhouse diversification (i.e. Early – Middle Eocene). This would imply that a number of taxa were able to adapt successfully to subsequent global cooling. Prior to the onset of global cooling in the late Middle Eocene, Antarctica may have served as a significant evolutionary centre for a variety of marine taxa. Source – sink models such as TNC and OTT, wherein the tropics act as an evolutionary source, and the poles as sinks, may be inappropriate for at least some major clades in a greenhouse world.

## Supporting Information

S1 Appendix **The early origin of the Antarctic marine fauna and its evolutionary implications: stratigraphy of Seymour Island.**
(DOC)

S2 Appendix **The early origin of the Antarctic marine fauna and its evolutionary implications: taxonomic appendix.**
(DOC)
