# Correction: The Early Origin of the Antarctic Marine Fauna and Its Evolutionary Implications

**DOI:** 10.1371/journal.pone.0120036

**Published:** 2015-03-24

**Authors:** The PLOS ONE Staff

Fig. 3 is incorrect. The authors have provided a corrected version here.

**Fig 3:**
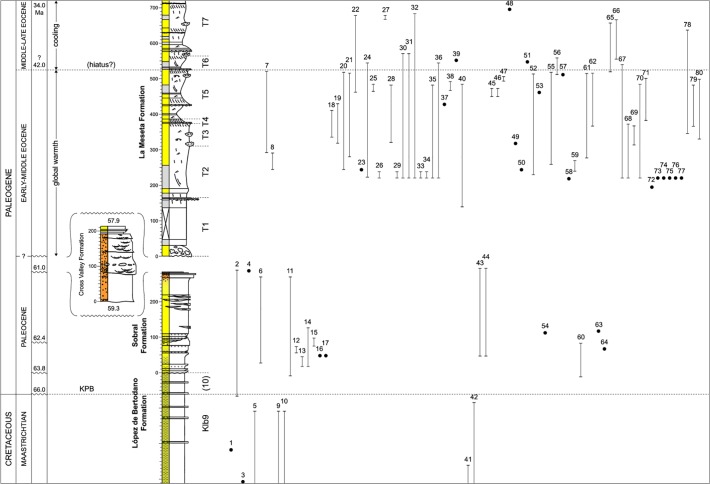
The stratigraphical radiation of the Neogastropoda in Antarctica. Solid lines and dots depict actual fossil occurrences and ranges of 80 neogastropod species. Full details as to how the occurrences and ranges were established within the stratigraphic framework are contained within the text and S2 Appendix. Klb 9 represents the topmost Maastrichtian stratigraphic unit of the LBF, KPB = Cretaceous/Paleogene boundary, and (10) is Kplb 10, the recovery interval and topmost informal stratigraphic unit of the LBF. T1–7 = Telms 1–7 of the LMF; ages (in Ma) are approximate and taken from Montes et al. [117] for the SF, CVF and topmost LMF. Further details on the age of the La Meseta Formation are given in S1 Appendix. Gastropods arranged in taxonomic order according to Bouchet & Rocroi [118]. 1.*Heteroterma* sp. 2; 2. *Heteroterma* sp.; 3. *Antarctissitys austrodema*; 4. *Pyropsis* sp.; 5. *Taioma charcotiana*; 6. *Taioma sobrali*; 7. *Taioma bicarinata*; 8. *Taioma*? *antarctocarinata*; 9. *“Cassidaria” mirabilis*; 10. Neogastropod, n. gen. B; 11. *Austrosphaera bulloides*; 12. n. gen. *woolfei*; 13. *“Colus” delrioae*; 14. *Probuccinum*? *palaiocostatum*; 15. cf. *Germonea* n. sp.; 16.?*Pseudotylostoma pyrinota*; 17. n. gen.? *polaris*; 18. *“Penion”* n. sp. A; 19. *“Penion”* n. sp. B; 20. *Penion australocapax*; 21. *Prosipho stilwelli*; 22. *Prosipho lawsi*; 23. *Prosipho delli*; 24. *Prosipho polaris*; 25. *Prosipho antarctocosta*; 26. *Prosipho* n. sp. 1; 27. *Prosipho lamesetaensis*; 28. *Pareuthria hookeri*; 29. *Pareuthria* n. sp. 1; 30. *Chlanidota antarctica*; 31. *Chlanidota tuberosa*; 32. *Chlanidota antarctohimaleos*; 33. *Chlanidota*?*antarctohimaleos*; 34. *Chlanidota* n. sp. 1; 35. *Austroficopsis seymourensis*; 36. *Austroficopsis wimani*; 37. *Austroficopsis australis*; 38. *Austroficopsis austrinus*; 39. *Austroficopsis meridionalis*; 40. n. gen. *verrucosa*; 41. Neogastropod, n. gen. A; 42. *Cryptorhytis philippiana*; 43. *Microfulgur binodosa*; 44. *Paleopsephaea*? *nodoprosta*; 45. *Fusinus*? *doylei*; 46. *Microfulgur byrdi*; 47. *Fusinus*? *eonodatus*; 48. *Fusinus*? *suraknisos*; 49. *Fusinus*? *graciloaustralis*; 50. *Trophon radwini*; 51. *Eupleura suroabdita*; 52. Turbinellidae indet.; 53. *Fulgurofusus brecheri*; 54. *Miomelon*? sp.; 55. *Adelomelon fordycei*; 56.?*Adelomelon suropsilos*; 57. *Odontocymbiola amundseni*; 58. *Miomelon antarctica*; 59. *Tractolira* n. sp.; 60. *Volutomitra*? *antarctmella*; 61. *Volutomitra*? *cernohorskyi*; 62. *Volutomitra*? *iredalei*; 63. *Marshallaria*? sp.; 64.?*Cosmasyrinx (Tholitoma) antarctigera*; 65. *Zemacies finlayi*; 66. *Aforia canalomos*; 67. *Marshallaria*? *oliveroi*; 68. *Austrotoma* n. sp.; 69. *Austrotoma*? *ventricosa*; 70. *Austrosullivania lata*; 71. *Austrosullivania striata*; 72. *Gemmula askinae*; 73. *Spirotropis*? n. sp.; 74. *Typhlomangelia*? n. sp.; 75. *Agladrillia*? n. sp.; 76. *Makiyamaia*? n. sp.; 77.? *Splendrillia antarctoliqua*; 78.? *Cochlespira brychiosinus*; 79. *Pristimercia australis*; 80. *Coptostomella*? *notopolaris*. Species 1–10 unassigned to superfamily; 11–49, Buccinoidea; 50–62, Muricoidea; 63–78, Conoidea; 79–80, Cancellarioidea.

## References

[1] Crame JA, Beu AG, Ineson JR, Francis JE, Whittle RJ, Bowman VC (2014) The Early Origin of the Antarctic Marine Fauna and Its Evolutionary Implications. PLoS ONE 9(12): e114743 doi:10.1371/journal.pone.0114743 25493546 10.1371/journal.pone.0114743 PMC4262473

