# Supplementary material for: The Early Origin of the Antarctic Marine Fauna and Its Evolutionary Implications
Source: PLoS One. 2014 Dec 10;9(12):e114743. doi: 10.1371/journal.pone.0114743 (PMC4262473; doi:10.1371/journal.pone.0114743)
Supplement: S1 Appendix — The early origin of the Antarctic marine fauna and its evolutionary implications: stratigraphy of Seymour Island. (DOC) [file pone.0114743.s001.doc]

**Appendix S1**

**The early origin of the Antarctic marine fauna and its evolutionary implications: Stratigraphy of Seymour Island**

1. *Latest Cretaceous and K/Pg boundary*

The ~900m thickness of Maastrichtian sediments exposed on Seymour Island and assigned to the López de Bertodano Formation represents a remarkably uniform succession of siltstones, fine sandstones and discontinuous concretionary horizons, with the uppermost levels being the most fossiliferous (Units Klb 6-9 of Sadler [10]) [17, 23, 121]. The Cretaceous – Paleogene boundary (KPB) is marked by a prominent interval of greenish-weathering glauconitic sandstones some 5-6m thick. At the base of this interval is a 1m sharp-based concretionary glauconite that is slightly, but clearly, diachronous when traced across central Seymour Island. This bed is equivalent to Zinsmeister’s ([122], p.557) “Lower Glauconite” whose base corresponds approximately to the occurrence of the last ammonite and a small iridium anomaly [123]. It has been used in this and other studies as the field marker for the KPB [23, 28, 86, 87]. Directly above the Lower Glauconite is a 2-3m interval of yellowish to tan-weathering siltstones and fine sandstones in which in-situ fossils are scarce apart from very obvious fish debris (scales, bones, teeth, etc). This “fish kill horizon” is directly overlain by a second prominent glauconitic sandstone, the ”Upper Glauconite”, which marks the top of the KPB interval ([122], fig. 3).

1. *Paleocene*

Directly overlying the Upper Glauconite there is a subtle change in lithologies to massive grey-brown mudstones and muddy siltstones bearing numerous small concretions. This is the 55 – 60m thick Unit Kplb 10 of the López de Bertodano Formation [10] which is characterised by a distinctive high abundance - low diversity molluscan fauna that includes the shallow-burrowing suspension feeding bivalves *Lahillia* and *Cucullaea*, deeper-burrowing, deposit feeding nuculids, and the probable deposit feeding gastropod, *Struthiochenopus* (see full list below). This is generally regarded as the “recovery fauna” that immediately post-dated the K/Pg mass extinction [16, 87, 124].

Unit Kplb 10 terminates in a slight angular discordance with the overlying Sobral Formation (SF), whose base is dated by Montes et al. [117] at 63.8 Ma using a combination of micropalaeontological techniques and strontium isotope stratigraphy (SIS). In section D9. 209/210 (Fig. 1) the SF comprises 296m of mudstones, muddy sandstones and sandstones with characteristic grey-green weathering hues. Finer-grained lithologies in the lower levels have distinctive, mottled yellowish-grey colouration and contain numerous macrofossils, many of which are associated with small concretions. A particularly rich concretionary interval between approximately 48 – 120m suggests a return to shelf-depth diversity and abundance levels last seen in the latest Maastrichtian (i.e. upper Unit Klb 9). Between approximately 80 – 92m a series of thicker, coarser sandstones with high glauconite content form a lithologically distinctive interval. The base of one of these sandstones, at ~92m, is sharply defined and, when traced laterally, can be shown to be discordant. This low-angle surface has been taken to mark the boundary between two allomembers (i.e. unconformity-bound packages) by Marenssi et al. [14] but in both this and previous studies there is no evidence to suggest that it marks a significant extinction event; the boundary is dated at 62.4 Ma [117]. Macrofossils become much scarcer in the upper SF and this can be linked to a marked change in sedimentary facies. For example, a distinctive set of sandstones between 185 – 230m shows the characteristic features of stacked ebb-tidal sandbars. A more typical SF macrofauna returns in the very highest levels of the unit and is dated at ~61Ma (i.e. Danian – Selandian boundary)[117].

The Cross Valley Formation (CVF) represents the fill of a deep erosional channel or valley system cut into older deposits. The principal area of outcrop in central Seymour Island (Fig. 1) measures ~1.5 x 0.6km in plan view and cuts down some 200m into the underlying SF. This channel fill comprises a thick succession of massively bedded, poorly sorted sandstones, pebbly sandstones and conglomerates which contain sandy – clay intervals, volcanic clasts and large fragments of carbonised wood. These deposits are of inferred fluvial origin and perhaps represent the product of flash floods from a volcanic hinterland on the Antarctic Peninsula [9, 10, 125]. In contrast, the topmost 25m of the CVF comprises thinner-bedded and finer-grained deposits that are in places distinctly fossiliferous (= CVF Allomember C of Marenssi et al. [14]). Leaf material occurs at several levels and there are at least two silty mudstone units with a relatively poorly preserved marine fauna of gastropods, bivalves, echinoids, crinoids, sharks teeth, and even penguin bones [126]. However, the most distinctive elements are large numbers of oysters, and fragments of both branching and encrusting bryozoans. Age control, which is very largely based on palynomorphs, suggests a date of 59.3Ma for the strongly onlapping, diachronous base of the CVF, and 58.6Ma for the base of Allomember C [117, 127]. This places the latter unit within the early Thanetian (i.e. Late Paleocene) with the top of the CVF being dated at ~57.9Ma [117].

1. *Eocene*

The stratigraphically highest unit, the La Meseta Formation (LMF), represents a second incised valley system cut into an emergent shelf during a period of lowered sea level. However, the principal valley is much larger than that of the CVF, measuring some 7km in its longest (NE – SW) axis by 4km at its widest; when traced westwards in the central Seymour Island region the LMF clearly onlaps onto progressively older beds (i.e. the CVF, SF and as low as Unit Klb 8 in the LBF (Fig. 1) [10, 11, 128]. The general form of the LMF depositional unit is that of a series of stacked lenticular bodies composed of mudstones, silty mudstones, fine – medium-grained sandstones, and shelly conglomerates [129, 130]. Such bodies have been interpreted to represent a complex network of shallow-water channels, such as are often encountered in estuarine and deltaic environments [9, 10]. Nevertheless, it has to be emphasised that the prolific fauna is fully marine, and a comprehensive stable isotope analysis was unable to detect any brackish or freshwater intervals [92]. Originally split into seven informal mapping units (“Tertiary Eocene La Meseta”, or Telms, 1-7) [10], the LMF stratigraphy was later refined into six erosionally based internal units, or Allomembers [11, 13]. In practice it has not always been possible to correlate either the Telms or Allomembers precisely across the full width of the depositional basin (see below), and the total thickness can only be estimated at ~720m [11].

The basal unconformity of the valley fill is a first rank surface of complex origin with more than 350m of relief [130]. It was initially linked by Sadler [10] to a late Ypresian (49Ma) global low sea-level stand and this was partially corroborated by micropalaeontological studies indicating an essentially late Early Eocene age for the earliest deposits [127, 131-133]. Nevertheless, strontium isotope stratigraphy subsequently placed the base of the LMF at 56.2Ma [13], although it will be pointed out below that dating the lower levels has proved more problematical than the uppermost ones. Whereas Telm 2 was dated as Early Eocene (~54.2 – 53.6Ma) by strontium isotopes, the spread of values for Telms 3-5 was more difficult to interpret; the base of Telm 5 was placed at 51Ma and its top at ~48Ma [92, table 2, fig. 8]. Telms 6 and 7 ranged from 43Ma (Middle Eocene) to ~34Ma (i.e. the Eocene – Oligocene boundary), and these ^87^Sr/^86^Sr dates for the topmost levels were consistent with current biostatigraphical age determinations [89, 90, 92, 133].

Nevertheless, in a recent re-examination of LMF dinoflagellate cyst biostratigraphy, Douglas et al. [93] determined that the Early Eocene was completely missing, and the entire sequence could be constrained within the age range of 45 – 34 Ma. Such an age model rests heavily on correlations with the magnetostratigraphically calibrated organic-walled dinoflagellate cyst biostratigraphic framework established recently for the SW Pacific Ocean and Wilkes Land margin of Antarctica [134]. Whilst these studies undoubtedly represent important advances in the Paleogene biostratigraphy of the southern high-latitudes we have found in practice that it is far from easy to establish definitive bio- and chrono-stratigraphies for the LMF, and considerable uncertainties remain as to how some of the principal lithological units fit together.

In many respects it is perhaps easier to work from the top downwards to establish an age model for the LMF. This is because there is general agreement that the most reliable part of the strontium isotope curve is that established for upper Telm 6 and the topmost Telm 7 [92,93]. Here, strong agreement between the ^87^Sr/^86^Sr ratios obtained and the global strontium isotope curve [135] indicate an age range between 41 Ma (upper Telm 6) and 34 Ma (top of Telm 7) ([92], fig. 8). Then, working down-section from that point the prominent extinction event at the Telm 5-6 boundary has been tentatively dated in this study at 42 Ma (Fig. 3). This is because the most likely cause of the extinction is some form of regional, or even global, cooling and 42 Ma may mark the initiation of ice caps on Antarctica [99,136]. It is even possible that this extinction in Antarctica is linked to the end-Lutetian event which is recorded globally in both the marine and terrestrial realms [137]. The prolific shell beds of Telm 5 could well be largely Lutetian in age and, as stated below, there is evidence to suggest that Telm 4 is simply a basal facies to the much larger Telm 5. So far, this interpretation of the age of the upper LMF is not strikingly different from that proposed solely on dinoflagellate cyst biostratigraphy by Douglas et al. [93].

The real problems with the LMF stratigraphy are in establishing the age and relationships of the basal three units, i.e. Telms 1-3. It does not appear that Telm 1 was sampled by either Ivany et al. [92] or Douglas et al. [93] and yet in its northernmost exposure it is up to 160m thick (see below). Similarly, Telm 2 is another complex unit that only appears to have been sampled at one locality by Ivany et al. [92]. It appears to be considerably thicker in the south than the north and, as demonstrated by the careful mapping of both Sadler [10] and Montes et al. [117], is almost certainly at least partially laterally equivalent to Telm 3. Some further detail on all these three units is given in the section below, but in our opinion their true relationship, and thus the true age of the base of the LMF, will only be established by a programme of further detailed mapping, section-measuring, and comprehensive sample collection for micropaleontology. It is for this reason that in this study we prefer to group Telms 1-5 under the informal heading of “Early – Middle Eocene” and Telms 6 & 7 as “Middle – Late Eocene” (Figs 2 & 3).

As most of the available fossil records for the LMF are tied to Telms rather than Allomembers, the former have been chosen as the primary lithostratigraphic units used in this study. Nevertheless, precise correspondence between the two was considered throughout using calibrations provided by both Marenssi et al. ([11], fig. 4) and Casadio et al. ([19], fig. 2). Telm 1 remains an enigmatic unit as it is only exposed at the northern end of the island within the Valle de las Focas immediately to the south of Cape Wiman, and in small, scattered exposures along the westernmost extension of the area of outcrop [10]. These two localities effectively represent opposite sides of the basin and it is clear that Telm 1 is an irregularly developed marginal facies. Up to 160m thick at the Valle de las Focas, it comprises thinly laminated, red-brown silts and fine sands closely associated with a megabreccia facies that includes large, reworked clasts of underlying SF and CVF (Fig. 1). A distinctive fauna includes large, flat specimens of *Ostrea antarctica* and broken fragments of the coarsely ribbed limid bivalve, *Acesta (Antarcticesta) laticosta* [18, 138]. In marked contrast, Telm 1 on the south-western margin of the basin comprises no more than a 2m thickness of shelly hash conglomerate interbedded with fine-grained pebbly sandstones; these lithologies are characterised by a deep, rusty-red colouration. Fossils are altogether more common in this region and include well-preserved serpulids, echinoids, corals, asterozoans and small bivalves, but the assemblage is dominated by some 22 species of brachiopods and 40 species of bryozoans [139-141]. This has the immediate appearance of a shallow-water, high-energy community such as would have been linked to predominantly hard substrates on the valley margin. Although Cabrera & Olivero [142] have suggested that these south-westernmost exposures of the LMF may in fact be lowermost Telm 2, this unique facies could also be interpreted as part of the initial high-energy depositional environments within the LMF basin (i.e. Telm 1).

It is likely that Telm 1 passes conformably both laterally and upwards into the ~140m-thick Telm 2, which is best exposed in the Cross Valley region between López de Bertodano Bay and Penguin Bay [10, 11] (Fig. 1). Here, the low topographic profile can be linked to a high mud content and lack of thick shell beds. The unit is characterised in its lower levels by dark, friable mudstones, very finely laminated siltstones, and fine sandstones bearing evidence of both slump folds and scar infills [10, 130]. Very thin coquinas (<5cm thick) are present and in places Telm 2 is intensely fossiliferous. The small nuculid bivalve, *Nucula nova*, in particular, is prolific in the mud-rich facies [18]. The relationship between Telm 2, which is best exposed towards the south-western end of the area of LMF outcrop, and the 92m-thick Telm 3, which is exclusive to the north-eastern end, is uncertain, but it is likely that they are at least partially laterally equivalent [10, 11, 130]. Telm 3 is characterised by buff-weathering, cross-bedded sands and silts, conglomeratic shelly sands and abundant shell beds showing a high standard of preservation [18, 85]. The upper limit of the unit is marked by the first *Cucullaea*-bearing, purple and green sands of Telm 4 ([10], p. 311).

Telm 4 comprises a distinctive, ~3m-thick *Cucullaea raea* shell bed that can be traced intermittently along the western flank of the LMF outcrop where it directly overlies Telm 3 in the north and Telm 2 in the south. It is clearly a ravinement surface with an associated transgressive lag characterised by abundant phosphate pebbles and glauconite, and as such could represent the first significant hiatus within the LMF [10, 11, 130]. Nevertheless, strontium isotope stratigraphy suggests that there may be no great temporal discontinuity here ([92], table 2 & fig. 8; 117), and it is also apparent that Telm 4 passes conformably up into Telm 5. Indeed, Stilwell & Zinsmeister ([18], p. 15) regarded Telm 4 as the initial shell bank facies of Telm 5.

Telm 5 is an approximately 100m-thick interval that forms more rugged relief and consists of laminated silty-sand layers alternating with bivalve-rich coquinas in which large forms of *Cucullaea* are particularly common. These *Cucullaea* shell accumulations come in two main configurations: persistent beds, 1-2m thick, that can be traced laterally for several kilometres, and thicker lenses that pinch out in a few metres ([10], p. 311). These shell beds are in places highly fossiliferous, and Telm 5 yields the most abundant and diverse marine invertebrate assemblage within the entire LMF [13, 18]. Sadler ([10], fig. 5) has emphasised that the overall form of Telm 5 is lensoid but its boundary with Telm 3 in particular needs to be carefully re-checked.

It should be stressed that that there is no evidence of a discontinuity in the field between Telms 5 and 6, apart from a marked facies change with the incoming of rusty-weathering sands alternating with venerid shell beds. This is the most significant lithological and facies change in the entire LMF section and is accompanied by a marked decrease in the marine invertebrate fauna [18, 92]. The ~40m-thick Telm 6 passes conformably up into the topmost unit, Telm 7 (~110m thick), which is typified by a variety of more resistant sandstone lithologies and some almost monospecific shell beds [10]. Telms 6 and 7 appear to be shallow-water, shoreface deposits [92, 130,].

**References**

(References cited in the main paper are not repeated here)

1. Olivero EB (2012) Sedimentary cycles, ammonite diversity and palaeoenvironmental changes in the Upper Cretaceous Marambio Group, Antarctica. Cret Res, 34, 348-366.
2. Zinsmeister WJ (1998) Discovery of fish mortality horizon at the K-T boundary on Seymour Island: Re-evaluation of events at the end of the Cretaceous. J Paleont, 72, 556-571.
3. Elliot DH, Askin RA, Kyte FT, Zinsmeister WJ (1994) Iridium and dinocysts at the Cretaceous – Tertiary boundary on Seymour Island, Antarctica: Implications for the K-T event. Geology, 22, 675-678.
4. Macellari CE (1988) Stratigraphy, sedimentology, and paleoecology of Upper Cretaceous/Paleocene shelf-deltaic sediments of Seymour Island. Geol Soc Am Mem, 169, 25-53.
5. Doktor M, Gaździcki A, Marenssi SA, Porębski SJ, Santillana SN, Vrba AV (1988). Argentine – Polish geological investigations on Seymour (Marambio) Island, Antarctica, 1988. Pol Pol Res, 9, 521-541.
6. Tambussi C, Reguero M, Marenssi SA, Santillana SN (2005) *Crossvallia unenwillia*, a new Spheniscidae (Sphenisciforme, Aves) from the late Paleocene of Antarctica. Geobios, 172, 1-9.
7. Wrenn JH, Hart GF (1988) Paleogene dinoflagellate cyst biostratigraphy of Seymour Island, Antarctica. Geol Soc Am Mem, 169, 321-447.
8. Marenssi SA, Net LI, Santillana SN (2002) Provenance, environmental and paleogeographic controls on sandstone composition in an incised-valley system: the Eocene La Meseta Formation, Seymour Island, Antarctica. Sed Geol, 150, 301-321.
9. Porębski SJ (1995) Facies architecture in a tectonically-controlled incised-valley estuary: La Meseta Formation (Eocene) of Seymour Island, Antarctic Peninsula. Stud Geol Pol, 107, 7-97.
10. Porębski SJ (2000) Shelf-valley compound fill produced by fault subsidence and eustatic sea-level changes, Eocene La Meseta Formation, Seymour Island, Antarctica. Geology, 28, 147-150.
11. Harwood DM (1988) Upper Cretaceous and lower Paleocene diatom and silicoflagellate biostratigraphy of Seymour Island, eastern Antarctic Peninsula. Geol Soc Am Mem, 169, 55-129.
12. Coccoza CD, Clarke CM (1992) Eocene microplankton from La Meseta Formation, northern Seymour Island. Ant Sci, 4, 355-362.
13. Askin RA (1997) Eocene - ?Earliest Oligocene terrestrial palynology of Seymour Island, Antarctica. The Antarctic region: geological evolution and processes (ed. C.A. Ricci), 993-996. Terra Antarctica Publications, Siena.
14. Bijl PK, Sluijs A, Brinkhuis H (2013) A magneto- and chemostratigraphically calibrated dinoflagellate cyst zonation of the early Paleogene South Pacific Ocean. Earth Sci Rev, 124, 1-31.
15. McArthur JM, Howarth RJ, Bailey TR (2001) Strontium isotope stratigraphy: LOWESS Version 3: Best fit to the marine Sr-isotope curve for 0-509 Ma and accompanying look-up table for deriving numerical age. J Geol, 109, 155-170.
16. Doria G, Royer DL, Wolfe AP, Fox A, Westgate JA, Beerling DJ (2011) Declining atmospheric CO_2_ during the late Middle Eocene climatic transition. Am J Sci, 311, 63-75.
17. Benton MJ (1995) Diversification and extinction in the history of life. Science, 268, 52-58.
18. Stilwell JD, Gaździcki A (1998) A new limid bivalve from the La Meseta Formation (Eocene) of Seymour Island, Antarctic Peninsula. Acta Geol Pol, 48, 149-155.
19. Bitner MA (1996) Brachiopods from the Eocene La Meseta Formation of Seymour Island, Antarctic Peninsula. Pal Pol, 55, 65-100.
20. Hara U (2001) Bryozoa from the Eocene of Seymour Island, Antarctic Peninsula. Pal Pol, 60, 33-156.
21. Hara U (2007) Biogeographical relationship of the South America – Antarctic Cenozoic bryozoan biota: The example of the austral genus *Aspidostoma*. Antarctica: A keystone in a changing world (ed. A.K. Cooper, C.R. Raymond et al.) USGS Open File Report 2007-1047, Extended Abstract 214, 6p.
22. Cabrera MIL, Olivero EB (2011) An Eocene articulated Polyplacophora (Mollusca) from the La Meseta Formation, Antarctica and the stratigraphy of the fossil-bearing strata. J Paleont, 85, 970-976.

**Taxonomic list of gastropods and bivalves from the López de Bertodano Formation, Units Klb 6-9.**

*Acmaea* n. sp. 1; *Leptomaria hickmanae* Harasewych, Oleinik & Zinsmeister, 2009; *Leptomaria antipodensis* Harasewych, Oleinik & Zinsmeister, 2009; *Amberleya spinigera* Wilckens, 1910; *“Cerithium”* n. sp.; *Mesalia* n. sp. A; *Mesalia* n. sp. B; *Capulus sulcatus* Wilckens, 1910; *“Vanikoropsis” arktowskiana* (Wilckens, 1910); *Amauropsis* n. sp.; *Struthiochenopus nordenskjoldi* (Wilckens, 1910); *Struthiochenopus* n. sp.; *Heteroterma* sp. 1; *Heteroterma* sp. 2; *Antarctissitys austrodema* Stilwell & Zinsmeister, 2003; Neogastropod, n. gen. A; Neogastropod, n. gen. B; *“Cassidaria” mirabilis* Wilckens, 1910; *Cryptorhytis*? *philippiana* Wilckens, 1910; *Taioma charcotiana* (Wilckens, 1910); *“Pyropsis” gracilis* Wilckens, 1910; *Acteon (Tenuiacteon) antarctihadrum* Stilwell & Zinsmeister, 2002; *Oligoptycha* cf. *O. concinna* Hall & Meek, 1854.

*Leionucula suboblonga* (Wilckens, 1907); *Solemya rossiana* Wilckens, 1910; *Australoneilo gracilis* (Wilckens, 1907); *Nordenskjoldia nordenskjoldi* Wilckens, 1910; *Austrocucullaea oliveroi* Zinsmeister & Macellari, 1988; *Cucullaea ellioti* Zinsmeister & Macellari, 1988; *Cucullaea antarctica* Wilckens, 1907; *Limopsis (Limopsis) antarctica* Wilckens, 1910; *Modiolus* cf. *M. pontotocensis* del Valle & Medina, 1980; *Pinna freneixae* Zinsmeister & Macellari, 1988; *Pinna anderssoni* Wilckens, 1910; *Phelopteria feldmanni* Zinsmeister & Macellari, 1988; *Pulvinites antarctica* Zinsmeister, 1978; *Entolium seymourensis* Zinsmeister & Macellari, 1988; *Entolium sadleri* Zinsmeister & Macellari, 1988; *Acesta shackletoni* Zinsmeister & Macellari, 1988; *Acesta webbi* Zinsmeister & Macellari, 1988; *Seymourtula antarctica* (Wilckens, 1910); *Limatula* sp. nov.?; *Pycnodonte (Phygraea)* cf.  *P. (P.) vesiculosa* (Sowerby, 1816); *Pycnodonte (Phygraea) seymourensis* (Wilckens, 1910); *Eselaevitrigonia regina* (Wilckens, 1910); *Oistotrigonia pygoscelium* (Wilckens, 1910); *Lucina scotti* (Wilckens, 1910); *Conchocele townsendi* (White, 1890); *Dozyia drygalskiana* (Wilckens, 1910); *Lahillia larseni* (Sharman & Newton. 1897); *Cyclorisma chaneyi* Zinsmeister & Macellari, 1988; *Surobula nucleus* (Wilckens, 1910); *Panopea clausa* Wilckens, 1910; *Goniomya hyriiformis* (Wilckens, 1910); *Thracia askinae* Zinsmeister & Macellari, 1988; *“Teredolites”* sp.

N.B. A number of unpublished manuscript names have not been accepted here; this is particularly so for several unsubstantiated neogastropod taxa.

**Taxonomic list of gastropods and bivalves from the López de Bertodano Formation, Unit Kplb 10.**

*Leptomaria seymourensis* Harasewych, Oleinik & Zinsmeister, 2009; *Perissodonta austerocallosa* Stilwell, Zinsmeister & Oleinik, 2004; *Struthiochenopus hurleyi Struthiochenopus* Stilwell, Zinsmeister & Oleinik, 2004; *“Vanikoropsis” arktowskiana* (Wilckens, 1910);*Austrosphaera bulloides* (Oleinik & Zinsmeister, 1996).

*Leionucula suboblonga* (Wilckens, 1907); Nuculanidae indet.; *Cucullaea ellioti* Zinsmeister & Macellari, 1988; *Pinna freneixae* Zinsmeister & Macellari, 1988; ? *Pycnodonte (Phygraea)* cf.  *P. (P.) vesiculosa* (Sowerby, 1816); *Lahillia larseni* (Sharman & Newton. 1897); *Marwickia/Cyclorisma* sp.; Lucinidae indet.

N.B. This list is based upon BAS field studies at localities DJ. 953 and D9. 207 (Fig. 1). Certain records from Stilwell et al. [16] that appear to occur less than 50m above the KPB have been excluded owing to uncertainties as to the accuracy of the stratigraphic plane technique.
