# Supplementary material for: The Early Origin of the Antarctic Marine Fauna and Its Evolutionary Implications
Source: PLoS One. 2014 Dec 10;9(12):e114743. doi: 10.1371/journal.pone.0114743 (PMC4262473; doi:10.1371/journal.pone.0114743)
Supplement: S2 Appendix — The early origin of the Antarctic marine fauna and its evolutionary implications: taxonomic appendix. (DOC) [file pone.0114743.s002.doc]

**Appendix S2**

**The early origin of the Antarctic marine fauna and its evolutionary implications: taxonomic appendix**

Specimen numbers for all material examined are given for each species described below; at the end of the Appendix geographical coordinates for all BAS localities mentioned in the paper are given.

Stratigraphic ranges for Paleocene samples from localities D9.209-211 are quoted in terms of either metres above the base of the Sobral Formation, or above the KPB].

**Maastrichtian, and Maastrichtian – Paleocene taxa**

*Heteroterma*? sp. 1 and 2; *Heteroterma* sp. (# 1,2, Fig. 3)

Material: *Heteroterma* sp. 1 – DJ. 957. 217 (10.5 – 22m in section DJ. 957), LBF unit Klb 7 of Sadler [10]. *Heteroterma* sp. 2 – DJ. 952. 186 (14 – 17m) (i.e. ~ 185 m beneath the KPB; Klb 8); DJ. 953. 40 (0 – 7.5m, Klb 9), 499, 569 (39 – 40.5m, i.e. ~48m beneath the KPB, Klb 9). *Heteroterma* sp. – DJ. 953. 103 (7.5 – 16.5m), 370, 500 (39 – 40.5m) (Klb 9); D9. 205. 256 (~ 8.5m beneath KPB; Klb 9). PRI collections – PRI 58301, 58389, 58400, 58817, 60567.

A group of distinctive, small (25 – 40mm), fusiform to biconical specimens from the uppermost levels of the López de Bertodano Formation have proved difficult to identify precisely (DJ.952.186; DJ. 953. 40, 103, 499, 500, 569; DJ. 957. 217; D9. 205.256). The spire typically comprises three complete whorls with slightly concave profiles and there is a short, straight to moderately recurved anterior canal. The peripheral angulation of the whorl is accentuated by a spiral row of blunt nodes, with approximately ten nodes per whorl. The simple aperture is lenticular in form and there are no folds on the straight to slightly recurved columella. Sculpture consists of fine, spiral riblets that are evenly spaced on both the whorl flanks and over the nodules. The strongly slanting growth line on the shoulder of the last whorl is not unlike that of certain turrids (= Conoidea).

The general form of these specimens suggests an assignment to either *Heteroterma* Gabb, 1869 or *Nekewis* Stewart, 1927. The former of these taxa tends to have a more strongly biconic shape and the latter is more pagodaform, but these differences are subtle and can be missed in small collections [143, 144]. The López de Bertodano specimens show a reasonably close resemblance to both the type specimen of *Heteroterma*, *H. trochoidea* Gabb (1869) and *Heteroterma*? *acrita* Saul 1988a, both from the Paleocene (Late Danian – mid-Selandian) of California. *Heteroterma*? *acrita* can in turn be compared with the Danian *H. zelandica* Marshall, 1917 from New Zealand and “*Cominella*” *praecursor* Wilckens 1907 (= *Heteroterma*? *tumida* (Wilckens)) from the Maastrichtian of southern Patagonia [145]. A second Danian species from the same region is *H. elegans* Griffin & Hunicken (1994).

Therefore, on balance, it would seem that the López de Bertodano specimens are closer to *Heteroterma* than *Nekewis*, and they are tentatively assigned to this genus. In addition two distinct species of *Heteroterma*? are identified: *Heteroterma*? sp. 1, although represented by only a single specimen (DJ.957.217), is distinguishable by its slightly larger size and two distinct spiral cords on the last whorl; *Heteroterma* ? sp.2 shows some variability in size and shape, and four specimens (DJ.953.103, 370, 500; D9. 205. 256) are only referred to *Heteroterma*? sp.

It is clear from examination of the Paleontological Research Institution (PRI) collections in Ithaca that W.J. Zinsmeister had collected a number of very similar specimens from the López de Bertodano Formation and assigned manuscript names to them. On the labels accompanying the specimens he used both “*Heteroterma*” and “*Nekewis”* but it is not easy to see the criteria he used to distinguish them. For example, whereas one of the taller specimens (PRI 58389), which resembles *H*.? sp. 1 of this study, has been labelled “*Heteroterma* n.sp.”, another (PRI 58817) is called “*Nekewis* sp.” WJZ also identified a series of smaller specimens (PRI 58301) as “*Nekewis macellari* n. sp.” But it is not clear how this taxon differs from either “*Nekewis* n. sp. B” (PRI 58400) or “*Nekewis* sp.” (PRI 60567). Stilwell et al. ([16], text-fig. 2) identified a “*Nekewis* n. sp.” that ranges through the latest Maastrichtian to stop at a level ~72m beneath the KPB. Such a range is very close to that of “*Heteroterma*? sp.2 of this study. Resolution of all these taxonomic problems will require a full revision of all southern high-latitude *Heteroterma/Nekewis*.

A further problem with these two taxa is their familial placement. Gabb [146] originally placed *Heteroterma* in the Pleurotomidae (=Turridae, i.e. Conoidea in modern classifications) and this practice was followed by Stewart [147], Finlay & Marwick [148] and Wenz [149]. Zinsmeister [143] emphasized the general similarity of *Heteroterma* to *Tudicla* Röding, 1798 and placed both it and *Nekewis* in the family Tudiclidae Finlay & Marwick, 1937; this taxon is now regarded as of subfamily rank within the Turbinellidae [118]. However, Popenoe & Saul [150] indicated that neither *Heteroterma* nor *Nekewis* have a tudiclid fold at the base of the columella, and neither has the characteristic expanded inner lip of *Tudicla spirillus*. In addition they both have an eye-shaped rather than a circular aperture. Popenoe & Saul [150] concluded that both *Heteroterma* and *Nekewis* were in fact closer to the Perissityidae Popenoe & Saul, 1987 than Tudiclidae, and this link was confirmed by Saul [144, 151]. Early Perissityidae clearly have some links to the Buccinoidea but in the classification of Bouchet & Rocroi [118] the family is unassigned to superfamily within the Neogastropoda.

It is also apparent that *Heteroterma* in particular can be confused with *Pyropsis* Conrad, 1860, and this is especially so with some of the Paleocene taxa from the southern high latitudes [152,153]. *Pyropsis* generally differs from *Heteroterma* by its more pyriform (pear-shaped) rather than biconical outline, wider pleural angle, and lower spire. The aperture is situated more posteriorly and the anterior canal is neither slightly twisted nor fasciolate [152]. Although it has been suggested by Squires [152] that the Pyropsidae should be placed within the superfamily Pyrifusoidea Bandel & Dockery [154], it is just as likely that *Pyropsis* can be accommodated within the Perissityidae [155].

For the purposes of this study *Heteroterma* , *Nekewis* and *Pyropsis* are retained within the Perissityidae, although it is noted that an alternative placement within the Tudiclidae has recently been advocated [153].

*Antarctissitys austrodema* Stilwell & Zinsmeister, 2003 [30] (# 3, Fig. 3)

Material: At least six specimens from localities 754, 1557, 1604 and 1606 [30]. These localities were located, as far as possible, on the USGS topographic map (many numbers are difficult to read on the pdf copy) and the positions then transferred to both the geological map and composite aerial photograph. It is likely that all four of these localities occur in the lower levels of López de Bertodano Formation unit Klb9, and this in turn translates to a level above the base of BAS locality DJ.952 ([23], figs 2 & 4) but ending in its mid-levels (~25-75m level). Although no specimens of *Antarctissitys austrodema* occur in the BAS collections it is likely that there are some in the as yet unidentified material contained in the WJZ collection, PRI. A group of four specimens bearing only the number PRI 62070 and the name “n. gen. B” could belong to this species.

*Antarctissitys austrodema* Stilwell & Zinsmeister, 2003 is another form that appears to be closely related to both *Heteroterma* and *Nekewis*. This distinctive species has a general pyriform shape but a lower spire makes it appear broader and more rounded in form. The smallest specimen illustrated by Stilwell & Zinsmeister ([30], fig. 2F,G) does bear some resemblance to *Heteroterma*? sp. 1 but the larger specimens are quite different. *Antarctissitys austrodema* is also characterised by two prominent, obliquely trending columella folds, and a variably developed parietal callus. The significance of the presence or absence of columellar folds in these taxa is unclear, and in some present day neogastropod families folds are present or absent in different genera.

Neogastropod, n. gen. A (# 41, Fig. 3)

Material : BAS DJ. 957. 107 (0 – 10.5m, LBF, Klb 7); DJ. 957. 210-216 (10.5 – 22m); DJ. 957. 240 (22 – 33.5m); PRI 58275: = PUI 425 (4453), field no. 89-33, “Maastrichtian, López de Bertodano Formation”; PRI 58431: from the drawer labelled ‘types’, no other information available; PRI 58590: PU N-84 (5072); López de Bertodano Formation, Klb8; PRI 60492: loc. 1546, no other details given. Judging from the BAS specimens, neogastropod n. gen. A is restricted to López de Bertodano Formation unit Klb7. However, specimen PRI 58590 is labelled as Klb8, so to take account of this the stratigraphic range is extended upwards to approximately the 50m level in section DJ. 957 (i.e. ~ 27m beneath thestart of section DJ. 952 (Fig. 1).

This is almost certainly a new genus and species of neogastropod with strong affinities to the family Fasciolariidae. At first sight there is a close resemblance to the genus *Anomalofusus* Wade, 1916. This is particularly so with certain Late Cretaceous taxa from the US Gulf Coast such as *Anomalofusus substriatus* Wade, 1916 and *A. lemniscatus* Sohl, 1964 ([156], figs 7,8, 11-13). The small to medium size, fusiform shape, lanceolate aperture and distinctive ornament pattern of well defined spiral cords superimposed on strong, axial costae all bring these Gulf Coast taxa to mind. Nevertheless there are differences in form, particularly in the whorl profile where the Antarctic taxon has less well rounded whorls and a more distinct sutural ramp. At this stage it is best to regard this new form as buccinoidean, very probably Fasciolariidae, but an informal new genus A.

It is clear from examination of the PRI collections that WJZ intended to describe this taxon as a new genus and species of gastropod and on various labels the manuscript name of “*Acoldium ornata* (= *ornatum*) n. sp.” is used. However, there is no trace of this genus in the palaeontological or zoological literature and *Acoldium ornatum* must be regarded as a *nomen nudum*. It is just possible that it is equivalent to “*Buccinum* n. sp. A” of Stilwell et al. ([16], text-fig. 2) but this taxon seems to be based on a single occurrence ~70m beneath the K-Pg boundary. This would in turn equate to a level of approximately the DJ.952/DJ.953 transition ([23], fig. 2), i.e. considerably above all the other closely spaced occurrences of n. gen. A of this study. There is simply no other information available on the identity of “*Buccinum* n. sp. A”.

Neogastropod, n. gen. B (# 10, Fig. 3)

Material : DJ. 952. 748, 749 (91 – 112.5m, LBF, Klb 9); DJ. 953. 174 (16.5 – 30m, Klb 9); DJ. 953. 347, 458, 568, 571 (39 – 40.5m, Klb 9); DJ. 957. 237-239 (22 – 33.5m, Klb 7); DJ. 957. 325-327 (33.5 – 45m, Klb 7); DJ. 957. 479 (88.5 – 99m, Klb 8); PRI 61573: loc. 1556, although only one PRI number is given there were approximately 15-20 specimens in the tray; PRI 58300: loc. 1436; this specimen bears the manuscript name “*Cibium grandis* (= *grande*) n. sp.”, but this must be another *nomen nudum*. *Cibium* (or *Cybium*) is a valid fish genus (WoRMS; http://www.marinespecies.org/) but there is no record of a gastropod genus bearing this name; PRI 58302: loc. 1439; also labelled “*Cibium grande* n. sp.”; PRI 62008: loc. 754; incomplete with much of the last whorl missing. In addition there are records of this taxon from the following WJZ field localities: K179, 1157, 1478, 1514, 1522, 1556, 1583, 1604, 1620, 1625. Stratigraphic range for this study taken from the BAS specimens only.

Small – medium (up to 80mm length) with a highly distinctive bucciniform profile. The last whorl in particular is characterised by a broad, gently sloping ramp that is further accentuated by a series of low, broad tubercles on the shoulder; this results in the well-rounded aperture appearing to project beyond the general outline of the shell (see the putative holotype, DJ. 957. 325). Both inner and outer lips present; short, deep anterior canal and well developed fasciole. Ornament of prominent tubercles present on the last four whorls. A number of the better preserved PRI specimens show that fine spiral threads are also present.

There is no obvious resemblance between this taxon and any other known Late Cretaceous neogastropod. This very distinctive new genus would appear to be much closer to the Buccinidae than Fasciolariidae.

“*Cassidaria*” *mirabilis* Wilckens, 1910 [157] (# 9, Fig. 3)

Material: DJ. 951. 4-23 (LBF, unit Klb 9, “close to camp” – see composite aerial photo); DJ. 952.47 (1.5 – 4m, Klb 9); DJ.952. 122 (4 – 14m); DJ. 952. 177-181 (14 – 17m); DJ. 952. 238 (17 – 29m); DJ. 952. 290, 296 (29 – 41m); DJ. 953. 273 (30 – 39m, Klb 9); DJ. 953. 570 (39 – 40.5m, ~48m beneath the KPB); DJ. 957. 80-83 (0 – 10.5m, Klb 7); DJ. 957. 348, 349 (45 – 55m, Klb 8); DJ. 957. 459 (69.5 – 88.5m, Klb 8); DJ. 957. 477, 478 (88.5 – 99m, Klb 8); DJ. 959. 78 (0 – 14m, Klb 7);DJ. 959. 217,218 (28.5 – 41m, Klb 7); PRI 58413 (= PU 1109; 4389), Maastrichtian, López de Bertodano Formation; PRI 60485 (= PU 1543; 4558), Maastrichtian, López de Bertodano Formation; PRI 60486 (= PU 1546; 4561), Maastrichtian, López de Bertodano Formation; PRI 61315 (= PU 1566; 4578). Stratigraphic range for this study taken from the BAS specimens only.

The general form of this medium – large species (>90mm length), prominent anterior canal and distinctive pattern of ornament strongly suggest that it is a neogastropod rather than a member of the genus *Galeodea* Link, 1807 (= *Cassidaria* Lamarck, 1816) in the Tonnoidea. There is a distinctive elongate – fusiform outline, with a narrow, pointed spire and acute apex. On some of the larger specimens (>12cm; e.g. PRI 58413, PRI 60485, PRI 60486, PRI 61315) it is clear that the outer lip is thickened and flared and thus the outline less symmetrical than in smaller specimens. Corresponding broad, smooth inner lip on best preserved specimens; some also show signs of a prominent parietal callus. The anterior canal is straight to slightly sinuous and of moderate length. Distinctive ornament style of small, pointed, closely spaced tubercles which in places coalesce to form strong spiral threads.

In his original description of “*C*.” *mirabilis*, Wilckens [157] also differentiated a new variety, *papillosa*. He compared the ornament of this form to both *“Nassa” papillosa* Linnaeus, 1758, as well as the genera *Pyropsis* and *Purpura* Bruguière, 1789. Although there is indeed a general resemblance in ornament style to a number of modern intertidal muricids, it is not possible at present to assign this taxon to a neogastropod family with any degree of certainty.

*Cryptorhytis*? *philippiana* Wilckens, 1910 [157] (# 42, Fig. 3)

Material : DJ. 951. 8,9,13,24,25 (LBF, Klb9, close to camp – see composite aerial photograph); DJ. 952. 182-184 (14 – 17m, Klb 8); DJ. 952. 237 (17 – 29m, Klb 9); DJ. 952. 289 (29 – 41m, Klb 9); DJ. 952. 348 (41 – 51.5m, Klb 9); DJ. 952. 442-446 (51.5 – 57.5m, Klb 9); DJ. 952. 557 (57.5 – 68.6m. Klb 9); DJ. 952. 630-640 (68.6 – 76m, Klb 9); DJ. 952. 754 (91 – 112.5m, Klb 9); DJ. 953. 41-47 (0 – 7.5m, Klb 9); DJ. 953. 97-102 (7.5m – 16.5m, Klb 9); DJ. 953. 173 (16.5 – 30m, Klb 9); DJ. 953. 248-251 (30 – 39m, Klb 9); DJ. 953. 346, 469-473, 492, 564-566 (39 – 40.5m, Klb 9, ~48m beneath the KPB); DJ. 957. 84-87 (0 – 10.5m, Klb 7); DJ. 957. 152-154 (10.5 – 22m, Klb 7); DJ. 957. 241, 242 (22 – 33.5m, Klb 7); DJ. 957. 276 (33.5 – 45m, Klb 7); DJ. 957. 350 (45 – 55m, Klb 8); DJ. 957. 413 (55 – 69.5m, Klb 8); DJ. 957. 476 (88.5 – 99m, Klb 8); DJ. 959. 83-85 (0 – 14m, Klb 7); DJ. 959. 139, 140, 145 (14 – 28.5m, Klb 7); DJ. 959. 219 (28.5 – 41m, Klb 7); DJ. 959. 248 (41 – 55m, Klb 7); DJ. 959. 332, 333 (55 – 70m, Klb 7); D9. 205. 293 (Klb 9, ~1m beneath KPB).

A common, medium-sized (<90mm) neogastropod with a fusinid/fasciolariid profile and distinctive ornament style. The latter comprises a combination of regular, narrow spiral cords that are superimposed on much stronger transverse costae. In this respect there are some resemblances to neogastropod n. gen. A of this study, especially in the smaller specimens (<50mm). Nevertheless *Cryptorhytis*? *philippiana* has a taller, more slender shell, the apical 3-4 whorls of which form an acute spire. In addition there is a distinctive whorl profile that is accentuated by a broad sutural ramp that slopes at a greater angle than in n. gen. A.; the shell profile is indeed close to that of a number of well known fasciolariids. In a collection of some 80+ specimens the anterior canal is always incomplete but there are indications that it would have been relatively long and spindle-shaped (see also [157], pl. 4, fig. 20).

In his original description of *Cryptorhytis philippiana*, Wilckens ([157], p.94-95) made comparisons with similar taxa assigned to the same genus from Trichinopoly, southern India, North America, and the Gosau Beds of Austria. The precise status of all these forms is still uncertain and thus assignment to *Cryptorhytis* has to be qualified. The genus is currently retained within the Fasciolariidae in Snyder’s [158] comprehensive catalogue of the family.

*Taioma charcotiana* (Wilckens, 1910) [157] (# 5, Fig. 3)

Material : DJ. 951. 1-3, 10-12, 22 (LBF, Klb9, close to camp – see composite aerial photo); DJ. 952. 12 (1-1.5m, Klb 9); DJ. 952. 35, 36 ((1.5 – 4m, Klb 9); DJ. 952. 124-136, 761, 762 (4 – 14m, Klb 9); DJ. 952. 153-172 (14 – 17m, Klb 9); DJ. 952. 217, 218 (17 – 29m, Klb 9); DJ. 952. 271-279, 295 (29 – 41m, Klb 9); DJ. 952. 345-347 (41 – 51.5m, Klb 9); DJ. 953. 94, 95 (7.5 – 16.5m, Klb 9); DJ. 953. 175-177 (16.5 – 30m, Klb 9); DJ. 953. 240, 243-246 (30 – 39m, Klb 9); DJ. 953. 465-468, 558 (39 – 40.5m, Klb 9, ~48m beneath the KPB); DJ. 957. 40-63 (0 – 10.5m, Klb 7); DJ. 957. 156-168 (10.5 – 22m, Klb 7); DJ. 957. 229-234, 255 (22 – 33.5m, Klb 7); DJ. 957. 264-267 (33.5 – 45m, Klb 7); DJ. 957. 440 (69.5 – 88.5m, Klb 8); DJ. 957. 475 (88.5 – 99m, Klb 8); DJ. 957. 513 (99 – 115m, Klb 8); DJ. 959. 214. (28.5 – 41m, Klb 7); DJ. 959. 236 (41 – 55m, Klb 7); DJ. 959. 323-325 (55 – 70m, Klb 7)

A common, medium-sized species, with occasional specimens exceeding 15cm in length. Strong fusinid profile with a narrow pointed spire, moderately long anterior canal and distinctive pagodaform whorl profile. Ornament comprises strong bicarinate whorls covered by finer spiral lirations. The aperture is rarely well preserved but there are indications that it is slightly flared with an accentuated outer lip.

This species was originally described by Wilckens [157] as *Fusus charcotianus*. It is clear from his text that he had some trouble in assigning the species to a precise generic category, suggesting that it showed affinities to both fusinids and turbinellids. In the end the closest form was stated to be *Serrifusus dakotensis* (Meek & Hayden, 1856) [159] from the Maastrichtian Fox Hills Formation of North Dakota ([160], pl. 17, figs 3 & 4), and there is indeed at least a superficial resemblance to this taxon. Wilckens [157] also noted that a close comparison could be made with “*Lagena*” *nodulosa* Stoliczka (1867) from the probable Maastrichtian of the Ariyalur Group of southern India, but this species is still very imperfectly known. In subsequent studies *Fusus charcotianus* has been referred to the genus *Taioma* Finlay & Marwick, 1937, and this decision was upheld in the comprehensive taxonomic review of New Zealand Cenozoic Mollusca by Beu & Raine [155]. These authors retained the family Taiomidae and indicated that it may have been more widespread both geographically and stratigraphically than previously thought. *Taioma patagonica* Griffin & Hünicken [145] from the Maastrichtian Cerro Cazador Formation of south-western Patagonia is a further possible member of the genus but is currently in need of further detailed investigation.

Taiomidae is at present best regarded as a Late Cretaceous – Early Cenozoic family currently unassigned to superfamily within the Neogastropoda [118] and restricted to Greenland and the southern high-latitudes.

“*Vanikoropsis*” *arktowskiana* (Wilckens, 1910)* [157]

Material: DJ. 951. 27, 28 (v.close to the base of DJ. 952, López de Bertodano Formation (LBF)); DJ. 952. 300-311 (29 – 41m in section DJ. 952); DJ. 952. 365-367 (41 – 51.5m); DJ. 952. 643, 644 (68.6 – 76m); DJ. 953. 358 (39 – 40.5m); DJ. 953. 618 (40.5 – 52.5m); DJ. 956. 11 (Cape Lamas, about 15 – 20m above the base of López de Berodano Formation unit Klb 2 (the so-called ‘dismal clay’); DJ. 957. 99, 100 (0 – 10.5m); DJ. 957. 278-287 (33.5 – 45m); DJ. 957. 353-363 (45 – 55m); DJ. 957. 427, 428 (55 – 69.5m); DJ. 957. 446, 447 (69.5 – 88.5m); DJ. 959. 82, 111 (0 – 14m); DJ. 959. 347 (55 – 70m); D9. 200. 12 (~ 5m beneath K/Pg); D9. 205. 253, 254 (~ 9m beneath K/Pg); D9. 206. 236 (~ 13m above K/Pg); D9. 209. 22 (0 – 25.5m); D9. 209. 253 (25.5 – 48m); D9. 209. 456-459 (48 – 69m); D9. 209. 626 (69 – 87m); D9. 209. 671-673 (69 – 87m); D9. 209. 742-746 (87 – 102m); ?D9. 210. 5 (42 – 51m); ?D9. 210. 74 (67.5m); D9. 211. 82, 83, 89 (48 – 57m); D9. 211. 185 – 204 (48 – 57m); D9. 211. 418-421 (27 – 48m); D9. 211. 62-67 (57m).

In addition, the following specimens assigned to *Vanikoropsis*? sp. were also used to define the stratigraphic range of this species: DJ. 951. 20 (close to the base of DJ. 952); DJ. 952. 240, 241 (17 – 29m); DJ. 952. 537 (57.5 – 68.6m); DJ. 952. 700 (76 – 91m); DJ. 953. 181, 181a (16.5 – 30m); DJ. 953. 259, 260 (30 – 39m); DJ. 953. 680, 681 (62 – 79m); DJ. 953. 925 (100 – 115m); DJ. 953. 1245, 1246 (143.5 – 154m); DJ. 958. 2 (~50m in DJ. 957); DJ. 959. 148, 149 (14 – 28.5m); D9. 206. 16 (~1.5m above K/Pg); D9. 209. 254 (25.5 – 48m); D9. 209. 463 (48 – 69m); D9. 211. 423 – 427 (27 – 48m).

Approximately 80 specimens are recorded in Stilwell et al. [16] but these were not used in the construction of the range plots.

This is a medium-sized (height typically 25-35mm, but occasionally larger), thick-shelled form with a distinct, high-spired, naticiform profile and well-rounded last whorl. The spire is approximately one-third of the total shell height and sharply pointed. The simple, ovate to sub-rounded aperture has an inner lip projecting broadly over a narrow, open umbilicus. The distinctive ornament pattern comprises regular, evenly spaced spiral cords that are broader than the interspaces; on some specimens the pattern is less regular where it is interrupted by pronounced prosocline growth lines. It is likely that this species ranges from approximately the base of the López de Bertodano Formation (Early Maastrichtian ?) to the top of the Sobral Formation (late Early Paleocene) on Seymour Island. It is entirely consistent in form throughout this range.

This species was originally assigned by Wilckens [157] to the genus *Eunaticina*?, with resemblances being noted to both *E. obliqua* Gabb from the Late Cretaceous of California and *E. ornata* Stoliczka from the Ariyalur Group of southern India [161]. In later Antarctic stratigraphic studies the species was simply referred to as *Eunaticina arktowskiana* [28, 124], and then transferred to *Vanikoropsis* Meek, 1876 (Family Vanikoridae; Hypsogastropoda) in the monograph by Stilwell et al. [16]. Such a placement agrees with the views of Sohl [162] and it is indeed clear that *V. artowskiana* is morphologically very close to *V. nebrascensis* (Meek & Hayden) from the Maastrichtian Pierre Shale and equivalent strata in North America; specimens such as USNM 132665 seem to be very similar indeed to the Antarctic material ([162], pl. 5, figs 14, 17). There is also a reasonable similarity to the three species provisionally assigned to *Vanikoropsis* from the Paleocene of West Greenland [163].

Nevertheless the assignment of this Antarctic taxon to the Vanikoridae, and *Vanikoropsis* in particular must be held in some doubt as it is almost certainly the only taxon that could have drilled the numerous predatory boreholes found in a variety of prey taxa throughout the Maastrichtian – Paleocene measured section. The only other naticids are small and rare (see below) and could not possibly account for the numerous boreholes. Some of these holes measure in excess of 6mm in diameter and could only realistically have been made by the largest specimens of “*Vanikoropsis*” *arktowskiana*. For this reason we prefer at the moment to keep the genus name in open nomenclature and believe that in future it should be formally assigned to a new genus within the Naticidae. Given the widespread occurrence of this species throughout the section we also think it unlikely to be a hard substrate-associated littorinid, although the similarities to this group are again quite striking [1]).

*This taxon is not shown on either Figs 2 or 3 but is included in this Appendix as it is clearly widespread throughout the Maastrichtian – Paleocene, and almost certainly a naticid.

*Leionucula suboblonga* (Wilckens, 1907) [164] (# 27, Fig. 2)

Material: WJZ Maastrichtian localities are listed in Zinsmeister & Macellari ([15], p. 256); these authors record the base of the *L. suboblonga* range as the top of Unit Klb6 (*sensu* Sadler [10]). BAS Maastrichtian material in stratigraphic order is as follows: DJ. 959. 101-108 (0 - 14m above base of section), 130-134 (14 - 28.5m), 210-213 (28.5 - 41m), 326-330 (55 – 70m); DJ. 952. 317 (29 – 41m), 499-505 (51.5 – 57.5m); DJ. 953. 359–361 (39 – 40.5m). The base of section DJ. 959 occurs at 397.5m beneath the KPB and is equivalent to the mid-levels of Klb7 ([23], p. 418); specimens DJ. 953. 359-361 occur ~39.5m beneath the KPB.

WJZ Paleocene localities are as follows: 9, 459, 477, 497, 514, 725, 754, 757, 762, 776, 777, 1119, 1130, 1131, 1134-1136, 1148, 1430-1432, 1467, 1473, 1502, 1505, 1506, 1510, 1519, 1697, 1529, 1531, 1533-1537, 1548, 1586. According to Stilwell et al ([16], p. 17 and table 4), *L. suboblonga* ranges up to approximately 162m above the KPB. This would place the upper limit close to the base of the strongly discordant glauconite at ~98m in the BAS Sobral Formation section (see below).

BAS specimens from unit Klb10, i.e. the recovery interval immediately above the KPB, are as follows: DJ. 953. 897–914, 922-924 (100 – 115m in section DJ. 953), 949-999, 1031-1032, 1043a, 1044, 1066-1076 (115 – 134.5m), 1110-1144 (134.5 – 143.5m), 1204-1220 (143.5 – 154m), 1310-1319 (154 – 160m); D9. 206. 175, 225-227; D9. 207. 6, 11, 18, 19, 21-45, 47, 57, 64-69, 98-112, 126, 136-158, 223-225. Specimens from the Sobral Formation are: DJ. 953. 1310-1319 (from within the basal 6m); D9. 211. 366-385, 488-490 (~ 22.5 – 79.5m in section D9. 209). Whether *N. suboblonga* ranges any higher in the Sobral Formation is currently uncertain (see below).

It is apparent that this common and stratigraphically wide-ranging species shows a considerable degree of variation in both size and form. With several specimens measuring over 40mm in length and 30mm in height it is certainly large for the genus and typically elongate (or “sub-oblong”) in form. The anterior margin is bluntly rounded and the ventral margin tends to be straight to slightly concave in its posterior section. The posterior margin is short and subtruncate, and a key taxonomic character would appear to be its angle with the hinge; nevertheless, it is apparent that this is a somewhat variable feature within a large collection and care must be exercised in its use. From the limited material available, it would appear that *Nucula oblonga* Wilckens ([164], pl. 6, fig. 1) from the Maastrichtian Cerro Cazador Formation of southern Patagonia has a longer and straighter posterior margin that forms a narrower angle with the hinge, but it is hard to see that *N. suboblonga* ([164], p.33) really has “more centrally located umbones”. Wilckens ([164], p. 33) also states that the umbo is narrower, the lunule smaller, and there is a more convex posterior hingeline that results in a less steep descent to the posterior margin. As Wilckens [164, p.33] had some 36 specimens of *N. oblonga* at his disposal we have to accept that these differences from his second species, *N. suboblonga*, are real but in view of the degree of variation within the latter in Antarctica a careful review of the Patagonian material is required. *Leionucula suboblonga* was retained as a valid taxon by both Zinsmeister & Macellari ([15], fig. 3, 1-5) and Stilwell et al. ([16], pl. 1, figs 1-4, 8).

*Limopsis (Limopsis) antarctica* Wilckens, 1910 [157] (# 29, Fig. 2)

Material: A single specimen is known from WJZ locality “St5” which is located immediately beneath the KPB in the central region of the area of outcrop [15, figs 1 & 2]. In addition, Wilckens ([157], p. 33) records two valves from Swedish South Polar Expedition Locality 8, which is slightly further south along the line of KPB outcrop, and very probably of latest Maastrichtian age. There are also records of five incomplete specimens from Maastrichtian strata on Snow Hill Island immediately to the south-west of Seymour Island. Approximately three of these definitely come from SSPE Locality 4 on the south-western corner of Spath Peninsula, with another four poorly preserved specimens probably originating from the same locality ([157], pp. 33 & 97). This material from SSPE Locality 4 is particularly interesting as it must occur at a much lower level within the López de Bertodano Formation, if not at its base ([23], figs 2 & 4). Extensive recollection within the López de Bertodano Formation since the SSPE has produced only a very small number of specimens [15].

The only specimen bearing the characteristic hinge and dentition of *Limopsis* is a right valve illustrated by Wilckens ([157], pl. 2, fig. 15) that probably comes from SSPE Locality 4 on Snow Hill Island. It is certainly larger than the left valve illustrated by the same author (pl. 2, fig. 14) but agrees well in general dimensions with the specimen described by Zinsmeister & Macellari ([15], p. 263 & fig. 8, 1). On balance, these authors believed that their material was closer to to the subgenus *Limopsis* Brocchi, 1814 than to *Pectunculina* d’Orbigny, 1843.

Although *Limopsis* has not been recorded from above the KPB on Seymour Island, five specimens of *Limopsis (Limopsista?) antarctominuta* are known from Eocene erratics within Mc Murdo Sound, East Antarctica ([165], p. 272 & pl. 2, fig. g). This is a tiny form with a height of no more than 4.5mm that bears a close resemblance to *Limopsis (Limopsista) microps* Finlay & Marwick ([148], p. 24 & pl. 1, figs 12 & 13) from the Paleocene of New Zealand. *Limopsis* is relatively common in the Early Miocene Cape Melville Formation of King George Island, South Shetland Islands [22] and is now the dominant bivalve genus in the modern Antarctic fauna [166].

*Conchocele townsendi* (White, 1890) [167] (# 33, Fig. 2)

Material: It is clear from the collections held within BAS that this is a stratigraphically wide-ranging species that can be traced down to at least the mid-levels of the Snow Hill Island Formation, the unit directly underlying the López de Bertodano Formation. It has been collected from both the Cape Lamb and Karlsen Cliffs members of this unit where it has an earliest Maastrichtian (or possibly latest Campanian?) age and lies at least 1200m beneath the KPB [23, 121]. Within the Karlsen Cliffs Member the occurrence of “*Thyasira” townsendi* are so prolific as to form well cemented limestone lenses up to 35m thick that mark the sites of former cold seeps [168]. Although cemented *“Thyasira”* pods are missing from the overlying López de Bertodano Formation, there are certain levels where the species re-occurs in reasonably large numbers, and this is usually in association with dark, sulphurous mudstones and occasional specimens of both *Solemya* and *“Lucina” scotti*. Smaller-scale cold seeps may have been a persistent feature of the Maastrichtian stage in Antarctica.

*“T.” townsendi* was collected as high as approximately 47m beneath the KPB as part of the last prolific bedding plane assemblage within the latest Maastrichtian. It has also been recovered at two levels within the Sobral Formation: an incomplete single valve, D9. 209. 613, from the 69-87m level, and a complete internal mold, D9. 209. 1186, from the 237-250m level.

Full descriptions of the species have been given by White [167], Weller [169], Wilckens [157] and Zinsmeister & Macellari [15]. In the latter paper there was some discussion as to whether the species could be referred to the subgenus *Thyasira (Conchocele)* Gabb, 1866 but on balance the authors decided that it was best left within *Thyasira, sensu stricto* ([15], p. 276). Nevertheless, examination of the large BAS collections suggests very strongly that the large size, very oblique form, almost straight anterior margin, and long, narrow sulcus are typical features of both modern and fossil species of *T. (Conchocele)* as defined by Gabb [146]. It is also apparent that *Conchocele* should now be regarded as a taxon of generic rank [24].

It should be noted that, strictly speaking, *Conchocele* occurs north of the Polar Front at the present day, but only just so in the Magellan region of southernmost Patagonia (in the form of *Conchocele fuegiensis*) [170,171].

*Thracia askinae* (Zinsmeister & Macellari, 1988) [15] (# 38, Fig. 2)

Material: A relatively uncommon but persistent species that, like *Conchocele townsendi*, can be traced back through the López de Bertodano Formation and down to at least the mid-levels of the underlying Snow Hill Island Formation [15, p. 282]. BAS material from the López de Bertodano Formation: DJ. 952. 392 (41 – 51.5m, i.e. Telm 9 and ~154m beneath the KPB); DJ. 957. 274 (33.5 – 45m, Telm 7), 519, 520 (99 – 115m, Telm 8); DJ. 959. 136 (14 – 28.5m, Telm 7).

A medium-sized species (with length typically in the 40mm+ range and height ~30mm), with a subquadrate outline and a right valve very slightly larger than the left. The posterior margin is distinctly rostrate and a well defined umbonal ridge extends to the posteroventral margin. Ornament typically comprises low, irregular growth increments ([15], fig. 16.4).

*Limatula* sp. nov. ? (# 30, Fig. 2)

Material: A small concretion from ~1.5m beneath the Lower Glauconite contains a current swept assemblage of tiny valves (<6mm) assignable to the modern genus *Limatula* Searles-Wood, 1839. They are characterised by a thin, translucent shell and very fine radial ornament; all are currently assigned to the BAS number D9.206.2. This material is clearly distinguishable from small specimens of *Seymourtula antarctica* (Wilckens, 1910) and shows some similarity to the modern *Limatula (Limatula) simillima* Thiele, 1912 [166]. Its stratigraphic position indicates an age of very latest Maastrichtian. The genus *Limatula, sensu lato,* had a wide stratigraphical and geographical distribution in the Cretaceous and may extend back to the Late Palaeozoic (http://paleobiodb.org).

**Paleocene taxa**

*Perissodonta austerocallosa* (Stilwell, Zinsmeister & Oleinik, 2004) [16] (# 7, Fig. 2)

Material: DJ. 953. 1322-1325 (topmost 6m, unit Klb 10); D9. 207. 176 (topmost 6m unit Klb 10); D9. 207. 220 (basal 6m, SF); D9. 211. 492 (0 – 27m, SF). There are also four specimens from two separate WJZ localities: 497 (1369m, i.e. 315m above the KPB, SF); 1434 (holotype) (1165m = 111m above the KPB, SF).

All the BAS specimens are incomplete and rather poorly preserved. Nevertheless they clearly comprise a distinctive group of small – medium (>35mm in length), robust, sub-globose shells with a prominent spire and thickened outer lip. In particular they show clear traces of a distinct spire callus (*sensu* Zinsmeister & Camacho) ([27], text-fig. 4) which partially, but not completely, envelopes the spire. The specimens can be confidently referred to the Struthiolariidae, and following the approach adopted by Beu [1] should be assigned to the modern southern high-latitude genus *Perissodonta* Martens, von [172] and not *Antarctodarwinella* Zinsmeister [173] (which, together with *Struthiolarella* Steinmann & Wilckens, 1908, is a subjective synonym of *Perissodonta*).

Of the six species of *Perissodonta* now recognised from the Eocene La Meseta Formation [18], the greatest resemblance is shown to *P.ellioti* (Zinsmeister) [173]. However, this species can be readily distinguished by its larger size, more prominent sinuous growth lines and massive callus on the inner lip ([27], pl. 1, figs 1-4).

*Amauropsis*? sp. (# 4, Fig. 2)

Material: D9. 211. 206, 225 (48 – 54m, SF); WJZ 1519 (1073m – i.e. 19m above the KPB); WJZ 1531 (1053m, i.e. ~1m beneath the KPB).

The BAS material comprises two small specimens of height 13mm and 10mm, respectively. Although the standard of preservation is less than optimum, it is clear that these are moderately high-spired, naticiform, bear a moderately impressed suture with a narrow, shallow channel, and lack any obvious strong ornament. In fact both specimens have a smooth, shiny appearance and overall there are considerable similarities with the modern polar naticid genus *Amauropsis* Mörch, 1857 and in particular to species such as *A. rossiana* Smith, *A. powelli* Dell, *A. anderssoni* (Strebel) and *A. (Kerguelenatica) grisea* (Martens) [166, 174].

*Amauropsis notoleptos* Stilwell, Zinsmeister & Oleinik [16] from the Sobral Formation is based on two specimens that again show significant similarities to modern *Amauropsis* species but are not particularly well preserved. A close link in form is noted by these authors to the type species, *A. islandica* (Gmelin, 1791), but this species is considerably larger than the fossil material, making precise comparisons difficult. *A. meierensis* Zinsmeister [143] and the very closely related species *A. martinezensis* Dickerson from the Late Paleocene of the Simi Hills, California do resemble the Antarctic specimens but can be distinguished by their more elevated spires ([143], fig. 2, K-M).

It is concluded that these four specimens represent a distinct taxon that shows considerable resemblance to *Amauropsis* but the poor preservation precludes a definite assignment to this genus. It should again be noted that the heights in the composite section given by Stilwell et al. [16, table 4] appear to be too low. According to the figures given, one of them occurs within the topmost Maastrichtian, and the other in lower Unit Kplb 10. The two BAS specimens come from the very fossiliferous interval in the lower Sobral Formation and are used to calculate the stratigraphic range in this study (Fig. 2).

*Euspira*? sp. (# 3, Fig. 2)

Material: D9. 209. 800 (102 – 120m, SF); D9. 209. 1180 (229.5 – 237m); Stilwell et al. [16] record seven specimens of *Euspira antarctidia* n. sp. from three WJZ localities: 1519, 1535 & 1538. It is clear that the first two of these lie within Unit Klb 10 of the López de Bertodano Formation but the latter, lying at approximately 49m above the KPB, could be close to the base of the Sobral Formation. It is unclear how Stilwell et al. ([16], p.35) derived a stratigraphic range of 1072 – 1130m for their new species (i.e. with the top of this range being 76m above the KPB). In neither their text-figs 2 nor 4 is the range shown as rising above 1100m.

This third probable naticid species from the Paleocene of Seymour Island is characterised by a series of small shells with a more compact and globose form. The two BAS specimens measure approximately 13mm (height) by 15mm (width) and 17 x 16mm, respectively, have a greatly expanded final whorl, and show little trace of any spiral ornament. There is some reasonably close resemblance between them and one of the paratypes of *Euspira antarctidia* Stilwell, Zinsmeister & Oleinik ([16], pl. 7, figs 8 & 10), but this taxon too, is not well preserved and cannot be assigned with confidence to *Euspira* [1]. Stilwell et al. ([16], p. 35) noted a possible link between *E. antarctidia* and *Euspira* cf. *E. pueyrredonensis* (Stanton) from the Paleocene Cerro Dorotea Formation of southwestern Patagonia ([145], figs 4.4 & 4.5).

As with *Amauropsis*? sp., it is unlikely that this comparatively small and rare species could account for anything other than a very small percentage of the predatory boreholes that occur throughout the Sobral Formation.

*Heteroterma* sp. (# 2, Fig. 3)

Material : D9. 209. 816 (102 – 120m, SF); D9. 210. 55-58 (42 – 51m); WJZ 1104 (1375m, i.e. 321m above the KPB)

Following the lines of argument given above for the Maastrichtian specimens, this rather poorly preserved material is referred to the genus *Heteroterma*. In general size and profile the specimens are closest to this genus, although the apertures and anterior canals are all incomplete. In style of ornament, D9. 209. 816 is closer to the Maastrichtian *Heteroterma* sp. 1, and D9. 210. 85-88 are closer to *Heteroterma* sp. 2. However, given the relatively poor preservation of the material no attempt has been made to extend the informal Maastrichtian taxonomic categories into the Paleocene. The specimens D9. 210. 55-58 are also close to that described by Stilwell et al. ([16], pl. 7, figs 13 & 14) (= WJZ 1104) as *Heteroterma*? n. sp.

Note that, as with *Austrosphaera bulloides* and *Microfulgur binodosa*,WJZ locality 1104 seems to be 20 – 30m too low as it occurs within the unfossiliferous concretionary unit near the top of the BAS measured section (Fig. 3 ).

*Pyropsis* sp. (# 4, Fig. 3)

Material : a single specimen, D9.210.59 (42-51m, i.e. uppermost Sobral Formation).

A small (width = 19mm, height ~ 20-25mm), incomplete specimen from which both the apex of the spire and the anterior canal is missing. Nevertheless, there are clear indications of a low spire and pyriform profile, and there is an immediate resemblance to small specimens of *Pyropsis zelandica* (Marshall) from the Wangaloa Formation of New Zealand (comparisons being made with specimens in the A. Grebneff Collection, GNS Science; see also ([148], pl. X, figs 8-10; 175, pl. 2, fig. p). Specimen D9.210.59 has approximately ten small tubercles on the shoulder of the final whorl upon which fine, regularly spaced spiral cords are superimposed. This pattern of ornament is close to that seen in *P. zelandica*.

Originally described as *Heteroterma zelandica* by Marshall [176], it is now apparent that this species should very probably be re-assigned to *Pyropsis* using the criteria outlined above under Maastrichtian *Heteroterma* [see also 151, 152, 177]. It can be distinguished from small, co-occurring specimens of *Tudiclana simulator* Finlay & Marwick (1937) by the latter’s higher spire and more fusiform outline ([175], pl. 2, fig. m). The precise taxonomic position of the single specimen assigned by Stilwell et al. [16] to *Pyropsis*? *australis* n. sp. is uncertain. Although it does resemble some specimens of the common *P. zelandica*, it differs from others, and may be significantly different from Late Cretaceous material of the NE Pacific assigned by Squires [152] to *Pyropsis*. Such confusion again highlights the need for a thorough taxonomic revision of *Heteroterma*, *Nekewis* and *Pyropsis* in the southern high latitudes.

*Taioma sobrali* Stilwell, Zinsmeister & Oleinik, 2004 [16] (# 6, Fig. 3)

Material : D9.209. 817 (102-120m, SF); D9. 209. 884, 886 (120-135m); N.B. D9. 209.886 is poorly preserved and only tentatively referred to *Taioma sobrali*? The following material was also used to establish the stratigraphic range: WJZ 497 (1369m, i.e. 315m above KPB); WJZ 1104 (321m); WJZ 1105 (315m); WJZ 1130 (40m); WJZ 1431 (90m). N.B. Once again, these WJZ heights seem to be on the low side; in particular WJZ 1130 (40m) would occur within the top of LBF Unit Kplb 10, and this seems to be too low.

Although Stilwell et al. [16] believed this species to be “medium-sized for the genus”, all the available material appears to be in the 30 – 40mm length range and thus considerably smaller than *Taioma charcotiana* or *T. bicarinata* (see below); it might be better classified as “small for the genus”. This species has a fusiform to pagodaform outline and comprises 4-5 noded, pagodaform whorls. The prominent last whorl is moderately inflated, bicarinate and with a steeply sloping sutural ramp. The noded keel comprises small, variably developed and closely spaced tubercles, and there is a variably developed ornament of fine, evenly and closely spaced spiral cords. Details of the apertural region are incomplete but the columella appears to be smooth and slightly concave.

Stilwell et al. ([16], pp.46 – 47) described *T. sobrali* as “extremely close morphologically” to *T. bicarinata* Stilwell & Zinsmeister [18] from the Eocene La Meseta Formation of Seymour Island. However, the latter is a somewhat larger species, with specimens in the BAS collections measuring up to 83 mm in length and 55 mm in width. In addition its tubercles are much more fully developed, and in the best-preserved specimens have distinct, blade-like tips. This style of ornament is also readily distinguishable from that of the Maastrichtian *T. charcotiana*, which also has a higher-spired, more fusiform shell and a different profile to the aperture. In general size and appearance *T. sobrali* is close to the type species, *T. tricarinata* Finlay & Marwick, 1937 from the Danian Wangaloa Formation of New Zealand, but this species is clearly tricarinate [16]. In his review of Antarctic Paleogene faunas, Beu [1] indicated that “*Marshallaria” variegata* Stilwell, Zinsmeister & Oleinik [16] from the Sobral Formation of Seymour Island might be reinterpreted as either immature specimens of *T. sobrali* or perhaps a new species within the same genus. Resolution of this issue will require careful re-examination of the type material.

At least one specimen from the Selandian Agatdal Formation of West Greenland [163] belongs within the Taiomidae [1, 163]. Thus *Taioma* is an austral (i.e. Weddellian) genus ranging from Late Cretaceous (Maastrichtian) to the late Early or Middle Eocene that also has a single occurrence in the Middle Paleocene of Greenland. It is currently retained within its own family but this in turn is unassigned to a superfamily within the Neogastropoda [118].

*Austrosphaera bulloides* (Oleinik & Zinsmeister, 1996) [52] (# 11, Fig. 3)

Material : DJ. 953. 1247-1251 (143.5 – 154m, i.e. 55.5 – 66m above the KPB; 154m = the topmost levels of Unit Klb 10); DJ. 953. 1320 – 1321 (154 – 160m, i.e. 66 – 72m above the KPB, = the basal 6m of the Sobral Fm [SF]); D9. 207. 202, 219 (48 – 52m, SF); D9. 209. 88 (17.2 – 25.5m); D9. 209. 460 (48 – 69m); D9. 209. 741 (87 – 102m); D9. 210.5 (42 – 51m); D9. 210. 74 (67.5m = top of section D9. 210); D9.211. 90 (48 – 57m); D9. 211. 208 – 213 (48 – 54m); D9. 211. 263 (48 – 54m); D9. 211. 428 – 430 (27 – 48m); PRI 58162 (= PU K-75; 5052) “*Austrosphaera patagonica* Feruglio, 1937”, Paleocene, Sobral Fm.; PRI 58854 (= PU 496; 4348) PU496 = 1179.42m, i.e. 125.4m above the K/Pg (= 1054m) “*Seymourosphaera elevata* Oleinik & Zinsmeister, 1996”; PRI 58858 (= PU 496; 4348) (see above) *“Seymourosphaera bulloides* Oleinik & Zinsmeister”, Sobral Fm.; PRI 58881 (= PU 746; 4354), 80.22m above the KPB, *Seymourosphaera* cf. *bulloides* Oleinik & Zinsmeister; Paleocene, Sobral Fm.; PRI 63229 (= RV 8200; 5069), “*Seymourosphaera* sp.”, N.B. label says “NW side of the prominent meseta at the northern end of Seymour Island”, Telm 4, La Meseta Fm (= an error?).

The stratigraphic occurrences used in this study are taken from the BAS field work in 1999 and 2010. As there is a possibility that *Austrosphaera* occurs again after a long gap at the top of the Sobral section, the WJZ locality 1104 was also marked on the section to reinforce the rather poorly preserved BAS specimens D9. 210. 5 and D9. 210. 74. However, when this is done locality 1104 plots in the middle of an unfossiliferous concretionary zone, and it is probably ~20m lower than the fossiliferous units at the top of the Sobral Fm. On looking at some of the other WJZ localities ([16], table 4), they also appear to be rather low. For example, 1189 plots at 17m above the K/Pg, 1134 at 29m above, and 1091 at 38m. We checked the incoming of *Austrosphaera* independently on two lines of section (DJ. 953 and D9. 207) and in both cases it only comes in at the very highest levels (i.e. within the topmost 5m of Unit Kplb 10). This casts some doubt on the stratigraphic plane method of analysis to estimate precise levels within the Sobral Formation from composite sections ([16], appendix; [17]).

The stratigraphically lowest neogastropod taxon to occur above the KPB first appears in the uppermost levels of Unit Kplb 10 of the López de Bertodano Formation and then throughout the greater part of the overlying Sobral Formation. It comprises a series of small (typically 40-45 mm in length), almost biconical to semi-spherical individuals in which a very short spire is almost completely covered by the last whorl. There is a distinctive concave columella, an almost smooth exterior, and an extensive callus but only a poorly developed fasciole. In their original description of this taxon, Oleinik & Zinsmeister [52] stressed the uncertain taxonomic affinities with links being made to certain genera within the Buccinidae, Nassariidae and Pseudolividae. In the end they assigned the Seymour Island specimens to a new genus, *Seymourosphaera*, which clearly had very close taxonomic links to *Austrosphaera* Camacho, 1949, and was tentatively placed within the subfamily Pseudolivinae, ? Family Buccinidae ([52], p.926).

The genus *Austrosphaera* is based on a single, incomplete specimen assigned to *A. glabra* Camacho, 1949 [178] from the Upper Cretaceous – Lower Paleocene Rio Bueno Formation, Tierra del Fuego. Camacho [179] indicated that *Cominella patagonica* Feruglio 1936 from the Paleocene Salamanca Formation, Chubut should also be included in this genus but is distinguishable from the type by its higher spire, less well developed callus, and presence of faint spiral ornament ([52], figs 4.7 – 4.9). With this generic concept in mind, Oleinik & Zinsmeister [52] determined that their Antarctic material should be referred to a new, closely related genus, *Seymourosphaera*, which was distinguished by more prominent spiral sculpture, a more concave columella, broader and thicker callus, narrower and shallower parietal and siphonal canals, and lack of a fasciole. Nevertheless, examination of the BAS and PRI collections (the types are, of course, missing!) together with the published descriptions reveals that all these characters are very variably developed and collectively insufficient to effect a separation at generic level. This study follows both Bandel & Stinnesbeck [180] and del Rio [153] in regarding *Seymourosphaera* as a synonym of *Austrosphaera*. It is possible that Oleinik & Zinsmeister [52] originally intended their taxon to be just a subgenus of *Austrosphaera* (see in particular their Fig. 6), but even this level of distinction is unwarranted. *Austrosphaera* is an endemic taxon from the Maastrichtian – Danian of central Chile, Patagonia and the Antarctic Peninsula [153].

A further difficulty with the concept of *Seymourosphaera* as developed by Oleinik & Zinsmeister [52] is their separation into four distinct species. The type species, *S. bulloides*, has a shell height of approximately 40mm, a subfusiform to biconical outline, a teleconch of four whorls, and variably developed spiral ornament. The height: width ratio is 1.39, spire height = 7mm maximum, and the average apical angle = 88°. It can be distinguished from *S. subglobosa,* which has a less elevated shell, lower spire and wider apical angle. *S. depressa* also has a wider apical angle but lower spire, wider aperture, and lack of radial sculpture on the spire whorls. Possibly the most distinctive of the four species is *S. elevata* which has a more elongate – oval profile, a half-moon shaped aperture, the smallest apical angle and absence of spiral sculpture ([52], figs 5.1 – 5.27).

*Seymourosphaera elevata* is the only species to occur in Unit Kplb 10 of the López de Bertodano Formation; it then continues upwards into the informal units 2 and 3 of the Sobral Formation where it co-occurs with the three other species. And it is within these three units that it has proved extremely difficult to differentiate between the four species using the criteria published by Oleinik & Zinsmeister [52]. These species simply grade one into the other and it would seem equally tenable that they represent one rather variable species. Therefore in this study all the Antarctic material has been referred to the one species, *Austrosphaera bulloides* [52].

Oleinik & Zinsmeister [52] established a set of morphological criteria for distinguishing their four species from both *A. glabra* Camacho and *A. patagonica* (Feruglio), and these distinctions have been accepted in this study. However, no detailed comparisons were made with the specimens from the Paleocene Cerro Dorotea Formation of south-western Patagonia assigned to *A. patagonica* by Griffin & Hunicken [145]. This material was subsequently re-examined by del Rio [153] who used further material from the Early Danian Roca Formation of the Neuquen Basin to reinforce the concept of *A. patagonica*. This species is clearly characterised by a last whorl bearing a well developed spiral depression, and a large, oval aperture that widens towards the middle of the whorl ([153], figs 3-16, 3-17). *A. patagonica* is distinguished from *A. glabra* by its less globose shell with a higher spire. Although it is just possible that the specimens of *A. patagonica* illustrated by Griffin & Hunicken ([145], figs 5.2 – 5.6) could be linked to the broadest forms of *A. bulloides* as defined in this study, it is more likely that they represent a new species [153]. The oldest known species of *Austrosphaera*, *A. difficilis* (d’Orbigny, 1842) from the Maastrichtian Quiriquina Formation of central Chile, has a very distinctive profile and high spire ([180], pl. 1, J, K; pl. 2, A-D).

n. gen. *woolfei* (Stilwell, Zinsmeister & Oleinik, 2004) [16] (# 12, Fig. 3)

Material : D9. 209. 461 (48 – 69m, SF); D9. 209. 619 (69 – 87m); D9. 211. 93 (48 – 57m); D9. 211. 215 – 224 (48 – 57m); D9. 211. 264 (48 – 57m); D9. 211. 364, 365 (27 – 48m); D9. 211. 408, 409 (27 – 48m); PRI 58639 (= PU9; 4340); PRI 61810 (= PU 1548; 4563)

Although relatively common (some 50+ known specimens in the BAS and PRI collections) this taxon is confined to a narrow stratigraphic interval in the lower levels of the Sobral Formation. According to Stilwell et al. ([16], table 4), it occurs between 114 and 125m above the KPB and in the BAS collections it occurs between 58.5 and 78m at locality D9. 209,and 7.5 - 52.5m at D9. 211. These BAS heights have been used to establish the vertical range in Figure 2.

A full description of this new taxon was given by Stilwell et al. ([16], pp. 40-41). In his revision of the Antarctic Paleogene molluscan fauna, Beu [1] suggested that there were significant differences from *Levifusus* Conrad, 1865, a northern taxon, and advocated assignment to a new genus. Stilwell et al. ([16], p. 41) did note a number of similarities with *Levifusus*? *quadrifunifer* Darragh [181] from the Paleocene Pebble Point Formation of Victoria, Australia, and the precise generic status of this species needs to be established too.

“*Colus*” *delrioae* Stilwell, Zinsmeister & Oleinik, 2004 [16] (# 13, Fig. 3)

Material: No BAS specimens; Stilwell et al. [16, p. 39] base their new species on three specimens from localities 1431 (1144m, i.e. 90m above the KPB, SF), 1535 (1078m / 24m), 1556 (not listed in Table 4); the holotype (UBA 16816) is poorly localised.

Concern is again expressed about the stratigraphic accuracy of locality WJZ 1535, as a position just 24m above the KPB would put this specimen in the middle of the recovery interval, Unit Klb 10 of the López de Bertodano Formation (and in Stilwell et al. ([16], p.80) locality 1535 is listed in the figure captions as “Sobral Formation”). Three further specimens were found, however, in the collections of the PRI, Ithaca, and these are listed as follows: PRI 61694 (= WJZ 1456, 1154m, i.e. 100m above the KPB); PRI 61720 (= 1435, 1165m / 111m); PRI 65456 (= 1587, 1134m / 80m). Therefore if the specimen from locality 1535 is excluded, the other four all come from a comparatively narrow interval ranging from approximately 80 – 111m above the KPB. Such a narrow range agrees well with that depicted in Stilwell et al. ([16], text-figs 2 & 4).

A medium – large (holotype = 90 mm height), high-spired, fusiform gastropod that is immediately distinguishable by its almost smooth form. The narrow, acute spire comprises approximately 50% of the shell height and exhibits at least six distinct whorls. The latter are very weakly rounded to almost flush and there is a narrow, channelled suture. The characteristic profile of the large last whorl is due to the sharp contraction at its base to produce a narrow and very slightly twisted anterior canal. The aperture is oval to sub-elliptical in shape and bears a thin, smooth outer lip. There are some traces of weakly developed growth lines on the best-preserved specimens, but no other signs of ornament.

In their description of this new species, Stilwell et al. [16] linked it firmly to the modern northern genus *Colus* Röding, 1798, even though the siphonal canal is clearly longer in the type species, *C. islandicus* Mohr, and twisted more strongly in an abaxial direction. Further examination of a series of *Colus* species housed in the Department of Invertebrate Zoology, National Museum of Natural History, Smithsonian Institution, confirms that the whorl profiles of *Colus s.s.* are consistently more convex and there is invariably a very fine spiral ornament. The presence of a longer anterior canal gives the aperture a more distinctive, obliquely elongated profile and in some species, such a *C. stimpsoni* (Mörch), this results in a more sinusoidal form to the columella. *“Colus” delrioae* has general buccinoidean features but should be assigned to a new genus.

*Probuccinum* ? *palaiocostatum* Stilwell, Zinsmeister & Oleinik, 2004 [16](# 14, Fig. 3)

Material : Stilwell et al. [15]record eight specimens from the following localities: WJZ 9 (1168m, i.e. 114m above the KPB, SF); WJZ 746 (1134m, = 80m); WJZ 1434 (1166m, = 112m); WJZ 1699 & 1701 (not listed in Table 4). In addition a series of specimens that can very probably be referred to this species are housed in the PRI: PRI 61945 (WJZ 1701), PRI 62390 (WJZ 1725), PRI 65448 (WJZ 4354) (none of these localities is listed in [16]), plus an unnumbered specimen herein referred to as PRI Specimen A. It is possible that the only specimens in the BAS collections that could be referred to this taxon are D9. 209. 584 (48 – 69m, SF) and D9. 209. 885 (120 – 135m).

Although the holotype has a height of 26mm, the two paratypes are 54mm and 44mm, respectively, and PRI 65448 and PRI Specimen A are both in the region of 65mm. This makes the fossil taxon considerably larger than all modern representatives of the genus, which typically fit in the 13 – 20mm size range. The teleoconch comprises five convex whorls and there is a prominent axial sculpture of broad, low folds that tend to fade across the last whorl ([16], p.41, pl. 10, figs 1-3). The aperture is sub-rounded to elliptical and there is a short, slightly twisted anterior canal. The holotype ([16], pl. 10, figs 1 & 2) certainly shows a resemblance to the modern genus *Probuccinum* Thiele, 1912, although it has to be borne in mind that this in itself is a somewhat variable taxon (as illustrated by e.g. ([182], pl. 55, figs 1-4). *Probuccinum palaiocostatum* is perhaps closest in general form to the modern *P. tenerum* (E.A. Smith) but the style of ornament can be linked to the much slenderer *P. costatum* Thiele. Because the match is not exact to any modern species it is perhaps best to only tentatively assign this fossil form to the genus *Probuccinum*. No other fossil taxa have been assigned to this genus.

cf. *Germonea* n. sp. (# 15, Fig. 3)

Material : D9. 211. 60 (48 – 57m, SF); D9. 211. 61 (putative holotype) (48-57m); ?D9. 209. 728 (87 - 102m);? D9. 211. 262 (48 – 54m).

Within the BAS collections there is a small group of specimens that can be related to a buccinoidean taxon characterised by a medium-sized, fusiform shell comprising 4-5 evenly ovate whorls and a distinctive ornament pattern where variable strength growth lines intersect fine and evenly spaced spiral cords to form, in places, a fine, cancellate pattern. The aperture is large, narrowly elliptical and with a short, broad siphonal canal that is slightly oblique to the shell axis. There is a long, gently curving columella and siphonal fold but no fasciole or pseudoumbilicus. The best preserved specimen and putative holotype of the new species is D9. 211.61, which has a length of 49mm and width of 27mm. The other BAS specimens are less well preserved but are closer to D9. 211. 61 than any other specimens in the collections.

This taxon immediately invites comparison with some of the larger modern Antarctic deep-sea buccinoideans. The general form and ornament style are not unlike that of the widespread species *Antarctoneptunea aurora* (Hedley) and the Scotia Sea species *Cavineptunea monstrosa* Powell. But by far the closest match is with a third species, *Germonea rachelae* [183], a species restricted today to abyssal depths of 2196 – 3714m in the Scotia Sea. The general form of the shell, style of the ornament and nature of the apertural region are close to those of both the holotype and all three paratypes, and even the partially preserved protoconch on specimen D9. 211.61 seems to be in agreement with that of *G. rachelae* ([183], figs 77-87). Further specimens would be desirable before definitely assigning these fossils to a modern genus, but the match is a close one and it may perhaps represent a more onshore occurrence of a taxon confined at the present day to abyssal depths.

If this identification of cf. *Germonea* n. sp. is correct then it immediately brings into question the status of at least some of the material currently assigned to *Zygomelon apheles* Stilwell, Zinsmeister & Oleinik, 2004. This is particularly so of the holotype (USNM 511896) which seems to be very close to the material described above. Stilwell et al. [16] attributed their new species to the volutid genus *Zygomelon* Harasewych & Marshall*,* 1995 largely on the basis of a single, oblique columellar fold. Traces of this fold seem to be present on one of the paratypes (USNM 511898) and it is possible that this specimen, together with USNM 511897, comprise a different taxon that is also characterised by a slightly more angular whorl profile ([16], pl. 10, figs 4, 7 & 8). USNM 511899 is a larger specimen that may be closer to cf. *Germonea* n. sp. ([16], pl. 10, figs 9 & 10). Careful examination of the BAS specimens indicates that there are no columellar folds.

?*Pseudotylostoma pyrinota* (Stilwell, Zinsmeister & Oleinik, 2004) [16] (# 16, Fig. 3)

Material: One specimen from WJZ locality 9 (1168m, i.e. 114m above the KPB, SF).

Originally assigned to the essentially northern genus *Sycostoma* Cox, 1931 by Stilwell et al. [16], it was pointed out by Beu [1] that a closer match may well be with the Patagonian Danian genus *Pseudotylostoma* Ihering, 1903. Although the two taxa currently assigned to this genus are poorly preserved ([145], figs 4.8 – 4.11), they do show a general resemblance to the solitary Antarctic specimen ([16], pl. 8, figs 23, 24). It is apparent that *Pseudotylostoma* is not a naticid, and Beu [1]), noting a general resemblance to *Austrosphaera*, tentatively placed it within the Buccinoidea.

n. gen.? *polaris* (Stilwell, Zinsmeister & Oleinik, 2004) [16] (# 17, Fig. 3)

Material: One specimen from WJZ locality 9 (1168m, i.e. 114m above the KPB). N.B. In the figure caption to the illustration of this taxon ([16], p. 81; pl. 8, figs 27, 28) it is attributed to the López de Bertodano Formation but this cannot be if this locality is correctly positioned at 1168m. Such a level is well within the Sobral Formation.

In his revision of the Antarctic Paleogene faunas, Beu [1] stressed that there were a number of significant differences from the Late Cretaceous – Oligocene northern genus *Strepisdura* Swainson, 1840, and suggested that it was more likely a new taxon within the Buccinoidea. It could well be related to the poorly preserved *Pseudofax*? *paucus* ([16], pl. 8, figs 25, 26) (also known from just a single specimen).

*Microfulgur binodosa* (Stilwell, Zinsmeister & Oleinik, 2004) [16] (# 43, Fig. 3)

Material: WJZ localities: 497 (= 1369m, i.e. 315m above KPB, SF); 746 (= 1134m, 80m); 1138 (= 1107m, 53m); 1104 (= 1375m, 321m); 1431 (= 1144m, 90m). N.B. Again, these values look on the low side; locality 1138 (53m above KPB) has been excluded because it falls within the upper levels of Unit Kplb 10, and both 497 and 1104 occur within the upper concretionary zone. The discrepancy may be because Stilwell et al. ([16], text-fig. 2) used a regional dip of 7.4° and BAS used a slightly higher value (10° on average). In addition Stilwell et al. ([16], p.42) gave the stratigraphic range as 1134 – 1375m (i.e. 80 – 321m above the KPB) but this clearly excluded locality 1138 (= 1107m) (see their Table 4); PRI 62380 (= PU 746; 4354).

Although Stilwell et al. [16] described this taxon as a new species within the North American Late Cretaceous genus *Serrifusus* Meek & Hayden, 1856, Beu [1] indicated that it was better placed within the New Zealand Paleocene genus *Microfulgur* Finlay & Marwick, 1937 (see also [155]). Like a number of other taxa, it is missing from the central, and probably shallower water, section of the Sobral Formation but reappears in the topmost levels (#43, Fig. 3).

*Paleopsephaea*? *nodoprosta* Stilwell, Zinsmeister & Oleinik (2004) [16] (# 44, Fig. 3)

Material: 12 WJZ specimens from the following localities: 746 – 1134m, SF (i.e. 80m above the KPB); 1104 – 1375m (321m); 1586 (148m); 1701. N.B. locality 1701 is listed on p.45 but does not appear in the list of localities and stratigraphic heights given in Table 4.

A small – medium biconic/fusiform shell (~45mm height) with 4 – 5 moderately strongly noded whorls. The last whorl in particular has a steep, moderately concave, adapical slope, and 12 – 14 axially extending nodes ([16], pl. 9, figs 6, 7, 11 – 16). There is a comparatively narrow, sublenticular aperture, and a short, slightly twisted anterior canal. Such features are all indicative of a small – medium fasciolariid, and a reasonably good match can be made to the type species of *Paleopsephaea*, *P. mutabilis* Wade from the Campanian – Maastrichtian Ripley Formation of the US Gulf Coast [16, 156]. This comparison is strengthened by the presence of narrow columellar plaits in the Antarctic specimens but somewhat weakened by the presence of a shallow anal sinus [1]. The possibility that *Paleopsephaea* could also be assigned to the Volutidae has already been discussed at length (e.g. [184]), and *Paleopsephaea neozelanica* Finlay & Marwick, 1937 from the Wangaloa Formation of New Zealand is almost certainly a turbinellid [175]. Thus it seems best at the moment to leave this taxon as only tentatively assigned to *Paleopsephaea*.

*Miomelon*? sp. (# 54, Fig. 3)

Material: One BAS specimen, D9. 209. 818 (102 – 120m, SF).

An incomplete fusiform to bucciniform internal mould that was probably originally in excess of 55mm in height and had a maximum width of at least 32mm. The specimen comprises a large last whorl together with a much smaller penultimate one. The suture between the two whorls is deeply impressed and there is a well developed, rounded shoulder. Sculpture consists of well-rounded axial ribs that extend across the entire width of the whorl in a gentle sinusoidal curve; interspace width is approximately equal to that of the ribs. There is an elongate – elliptical aperture but the nearly straight columella is rather poorly preserved and bears no trace of plaits.

The specimen bears a general resemblance to fossil and modern representatives of the volutid genus *Miomelon,* which comprises approximately four fossil and four living species from southernmost Argentina and Chile; in this region it has a fossil record spanning Eocene – Miocene [185]. However, there is no precise match to any known species within this genus and the assignment can only be regarded as provisional. An alternative position may be within *Palaeomelon*, a Neogene taxon found along the Pacific coast of Chile; however, this genus is characterised by a more elongate spire, the teleoconch whorls are more acutely shouldered, and the axial ribs less continuous from suture to suture [185].

*Volutomitra*? *antarctmella* (Stilwell, Zinsmeister & Oleinik, 2004) [16] (# 63, Fig. 3)

Material: Two specimens from localities 1108 and 1635, SF; these are rather imprecisely placed at ~ 121 – 156m above the KPB ([16], p. 48).

This is a small fusiform species (height ~20 – 33mm) with rounded - subquadrate whorls that have a distinctive telescoped profile ([16], pl. 10, figs 11 – 14). The last whorl in particular shows the pattern of a subsutural swollen collar that passes aborally into a marked constriction that can be traced around the entire whorl. An unusual style of ornament comprises a row of small, well-rounded nodules on the shoulder of each whorl together with a reticulate pattern of fine spiral cords and orthocline growth lines. There is a narrow, lenticular aperture and a gently concave columella bears at least two closely spaced columellar folds [16].

It would seem unlikely that this species can be referred to either the essentially tropical genus *Mitra* or the subgenus *Eumitra*. Further examination of both the types and the new BAS specimen will be necessary and in the interim it would be better to tentatively refer it to a southern taxon such as *Volutomitra* or, perhaps, *Proximitra.*

*Marshallaria*? sp. (# 63, Fig. 3)

Material: D9. 209. 815 (102 – 120m, SF).

A single specimen of a small – medium (height = 25 mm), fusiform ‘turrid’ with a broad sinus clearly visible on the gently concave sutural ramp. The ornament comprises prominent and closely spaced spiral cords that are crossed by narrow axial costellae; these are especially prominent on the whorl shoulders. There is a lenticular/eye-shaped aperture and smooth, gently concave columella.

There is quite a strong resemblance to the genus *Marshallaria* from the Paleocene – Miocene of New Zealand and Australia, and in particular to the type species, *M. multicincta* (Marshall) from the Wangaloa Formation ([148], pl. XI, figs 10-12). However, confirmation of this generic assignment will depend on further material becoming available. As noted above, *M. variegata* [16] may in fact be a *Taioma*.

?*Cosmasyrinx (Tholitoma) antarctigera* Stilwell, Zinsmeister & Oleinik (2004) [16] (# 64, Fig. 3)

Material: Stilwell et al. ([16], p. 50] state that the three specimens come from three separate localities: 496, 1519 and 1535, SF. Whereas the former of these localities occurs at 1179m (i.e. 125m above the KPB), the latter two are at levels of 18m and 23m respectively above the KPB. These last two stratigraphic positions seem unlikely as this would put them in the lower levels of López de Bertodano Formation Unit Klb 10. Locality 496 plots at 125m above the KPB and this correlates with the fossiliferous interval low down in section D9. 209, and the whole of D9. 211; this is the sole position indicated on Fig. 3 for this species. The stratigraphic range plotted for *C. (T.) antarctigera* in Stilwell et al. ([16], text-fig. 2) seems to be in error as over 20 occurrences are given, as well as a long range through the late Maastrichtian. The latter occurrence need to be substantiated.

This second probable conoidean from the Sobral Formation has been identified as a new species within the subgenus *Cosmasyrinx (Tholitoma)* Finlay & Marwick (1937) by Stilwell et al. [16]. Only three specimens are known, but the preservation of the paratype (USNM 511908) is such as to suggest a close resemblance to the type species, *Cosmasyrinx (Tholitoma) dolorosa* Finlay & Marwick ([148], pl. XII, figs 6, 7 & 11) [1]. It is a comparatively small form (holotype height = 7.0mm). with a distinctive pagodaform outline and sharply defined keel on the last whorl. A moderately deep anal sinus is present on the middle part of the shoulder slope ([16], pl. 10, figs 15, 16).

*Leionucula hunickeni* (Zinsmeister & Macellari, 1988)? [15] (# 27, Fig. 2)

Material: 31 specimens from localities 9, 497, 746, 1104, 1505, 1519 & 1534, Sobral Formation [16, p. 17 and table 4].

The precise taxonomic status of this species is in some doubt. It clearly co-occurs with *N. suboblonga* as two of its localities are within Kplb10 (i.e. 1519 and 1534) and three more are within the fossiliferous lower to mid- levels of the Sobral Formation (i.e. 1126, 1134, 1168). Zinsmeister & Macellari ([15], p. 256 & fig. 3, 10-11) distinguished it by the smaller size and “straighter anterior” [sic = posterior margin], which “slopes at a steeper angle” (presumably a more acute angle with respect to the hinge?). Using these two criteria alone it is not entirely clear how this species differs from *L. oblonga* (Wilckens) (see above), and when comparisons are made with small specimens found in amongst large populations of *L. suboblonga* the distinction becomes even more blurred. The holotype of *L. hunickeni* ([15], fig. 3, 10-11) does appear to have a more rounded ventral margin, particularly in the posterior section, and the lunule is much less conspicuous. However, whether this is a consistent feature maintained throughout the stratigraphic range within the Sobral Formation is uncertain. Later collections of small nuculids ranging throughout the SF on the whole show greater affinity with *L. suboblonga.*

*Parathyasira austrosulca* (Stilwell, Zinsmeister & Oleinik, 2004) [16] (# 32, Fig. 2)

Material: Two specimens from locality 1519, Sobral Formation. Stilwell et al. ([16], p. p. 22 and table 4) give the stratigraphic height of locality 1519 as 1073m but if this is correct then it would place the specimens only 19m above the KPB, i.e. well within Unit Kplb 10 of the López de Bertodano Formation. A much more likely stratigraphic position is given in Text-figure 2 within the same paper where *T. austrosulca* n. sp. occurs at ~1169m, i.e. ~115m above the KPB and 54m above the base of the SF.

This is a minute (maximum height = 5.5mm), erect to subcircular, strongly prosogyrous form with a very clearly defined sulcus on the posterodorsal margin, and very little ornament ([16], pl. 3, figs 15 & 16). In their discussion of this species, Stilwell et al. ([16], p. 22) made a strong comparison with the modern Antarctic species, *Thyasira dearborni* Nicol, 1965, noting only that the fossil form was a “bit smaller” and the posterior sulcus “slightly broader” (see also [166], pp. 56-57 & figs 91, 92; [24], fig. 47; [182], pl. 12, figs 7a, b). Such a comparison does indeed seem to be valid, but it should be noted that *T. dearborni* is now placed within the genus *Parathyasira* Iredale, 1930 [24]; this would seem to be a suitable generic assignment for the SF species too.

**Eocene taxa**

*Calliotropis antarchais* Stilwell, 2005 [186] (# 1, Fig. 2)

Material: USNM 523858 (holotype) and USNM 523859, 523860 (paratypes) from locality 456, central Telm 5 ([186], p.11).

Appears to be restricted to Telm 5, La Meseta Formation (LMF); superfamily Seguenzioidea, *sensu* [118].

*Collonia rara* (Stilwell & Zinsmeister, 1992) [18] (# 2, Fig. 2)

Material: One specimen from locality 443, Telm 5 ([18], p. 93).

Stilwell & Zinsmeister [18, p. 93] indicated that the precise generic assignment of their *Calliostoma (Maurea) rara* was uncertain, and Beu ([1], p. 216) suggested that this single specimen was in fact closer to *Collonia* Gray, 1850 (family Colloniidae, superfamily Turbinoidea, *sensu* [118].

*Falsilunatia* sp. (# 5, Fig. 2)

Material: Stilwell & Zinsmeister ([18], p. 110) assign 906 specimens to *Polinices (Polinices)* cf. *P. (P.) subtenuis* (Ihering, 1897). These are from the following localities: 1-6, 8, 10, 14-16, 18, 19, 22, 23, 25, 26, 28, 29, 443-445, 447, 450, 451, 453, 455-457, 462, 467, 469-471, 486, 487, 489, 491, 577, 584, 586, 591, 595-597, 610-612, 630, 782, 1057, 1058, 1061, 1063, 1064, 1068-1072, 1076, 1079, 1082, 1085, 1086-1089, 1091-1094 & 1097 (Telms 1-7, LMF).

In addition, 42 specimens are assigned by the same authors to *P. (P.) marambioensis* Stilwell & Zinsmeister ([18], p. 111]. These are from localities: 14, 15, 25, 28, 443, 456, 466, 482, 532, & 610 (Telms 3-5). Approximately 219 BAS specimens were collected from the following localities: DJ. 76. 1 (loose, probably Telm 3); DJ. 77. 1-14, 130-143, 197, 237-264, 345-356 (Telm 3); DJ. 79. 229-278 (Telm 3); DJ. 81. 42-86, 103 (Telm 4); D9. 212. 409-432, 501, 502, 3165-3190 (all juveniles - Telm 2, on the northern flank of Cross Valley).

After careful consideration and review of a large number of specimens it is considered likely that the vast majority of naticids within the La Meseta Formation belong to one very common and rather variable species. At almost all localities there is a mixture of both adult and juvenile specimens, and overall size ranges from just a few mm in height and width to approximately 45mm height and 55mm width. Well preserved shells are undoubtedly naticiform – globular in shape, with a globose last whorl that envelopes most, but not all, of the previous whorls. This leaves a short spire where the tops of 2-3 of the earliest whorls are exposed together with what appears to be a simple, planispiral protoconch. The aperture is large, subovate to D-shaped, with a comparatively small umbilicus and moderately thick callus. On the best preserved specimens the ornament comprises fine, sinuous growth lines, with virtually no trace of any spiral elements.

What is also apparent is that the last whorl erodes rather easily, with the posterior part in particular often being lost from the spire. This gives the shell a much more evolute appearance, with many specimens looking at first glance to be far from the globose – naticiform profile of the best preserved material. In addition, erosion of the last whorl often accentuates the spiral growth lines making them appear to be much stronger than they really are. Added to the wide variation in size, this often gives the impression that large collections of these naticids are taxonomically more variable than in fact they are.

Beu ([1], p. 216) indicated that the two commonest naticids from the La Meseta Formation are much more easily referable to the modern Antarctic genus *Falsilunatia* Powell, 1951 than the essentially tropical *Polinices* Montfort, 1810. As *Polinices subtenuis* Ihering should be reassigned to *Glossaulax* Pilsbry, 1929, the Antarctic material tentatively assigned to this species by Stilwell & Zinsmeister [18] should now be placed in *Falsilunatia* n. sp. ([1], p. 216). It is highly likely that *P. (P.) marambioensis* Stilwell & Zinsmeister should be assigned here too, as the grounds for its separation as a distinct species are slender. It is certainly not larger than *P. (P.)* cf. s*ubtenuis* and both spire height and last whorl inflation are highly variable features within the La Meseta Formation naticids ([18], p. 111).

*Sinuber powelli* (Stilwell & Zinsmeister, 1992) [18] (# 6, Fig. 2)

Material: A single specimen from locality 4 (Telm 4, LMF) ([18], p. 112).

A medium-sized (height = 20mm, width = 18mm) subglobose specimen with a moderately tall spire, expanded, convex last whorl, and large, subovate aperture. There is no obvious funicle or callus. Although it appears at first sight to be almost smooth, there are traces of fine, spiral ornament on the last whorl ([18], p.112). It is this latter feature, together with the form of the spire, that indicates a position within *Sinuber* Powell, 1951 rather than *Globisinum* Marwick, 1924 ([1, p.216).

*Perissodonta ellioti* (Zinsmeister, 1976) [173] (# 7, Fig. 2)

Material: 202 specimens from the following localities: 1, 8, 24, 26, 447, 451, 452, 461, 462, 469, 479, 584, 586, 612, 615, 1068-1070, 1085, 1087, 1088, 1091, & 1094 (Telms 2 & 3, LMF) ([18], p. 109); 58 specimens from DJ. 77 (Telm 3): DJ. 77. 15-24, 130-132, 214-234, 315-338; 19 specimens from DJ. 79 (Telm 3): DJ. 79.208-226; 13 rather poorly preserved specimens from D9. 212 (Telm 2) probably belong to this species: D9.212. 3091-3096, 3356, 3377-3378, 3386-3389; 25 specimens were collected loose at DJ.76, Cape Wiman region (but are either Telm 2 or 3): DJ. 76. 2-26.

This species is particularly common in Telms 2 and 3 and in places is a major component of the prominent coquinas. Some specimens within the BAS collections, such as DJ. 77. 316, measure up to 55mm in height and 44mm in width, and it is clear that adults show a considerable range in size variation ([27], pl. 1, figs 1-4; [18], pl. 14, figs f-i). The shell typically has a broad, robust form with a low to moderately elevated spire and a last whorl sculpture of numerous, sinuous, varix-like growth increments [18]. A distinctive feature of the species is a massive callus on the inner lip that extends well above the aperture and onto the spire. As noted above, *Antarctodarwinella* Zinsmeister (1976) is a subjective synonym of *Perissodonta* Martens, 1878*.* Stilwell & Zinsmeister ([18], p. 109) noted the superficial resemblance of *P. ellioti* to *Conchothyra australis* (Marshall) from the Wangaloan (Danian) of New Zealand [148], and Zinsmeister & Camacho ([27], text-fig. 7) suggested that Late Cretaceous *Conchothyra* from New Zealand may be stem to the entire Struthiolariidae lineage. However, *Conchothyra australis* is much larger and more smoothly and heavily callused than *Perissodonta ellioti*. No Late Cretaceous Struthiolariidae are known from Antarctica.

*Perissodonta nordenskjoeldi* (Wilckens, 1911) [188] (# 7, Fig. 2)

Material: 260 specimens from the following localities: 4, 5, 10, 15, 25, 28, 443, 445, 450, 453, 455-457, 466, 467, 472?, 486, 489?, 584?, 595, 597, 610, 1057, 1058, 1079, 1081, & 1089 (Telms 3-5, LMF)[18, p. 109]; 17 specimens from DJ. 81 (Telm 4): DJ. 81. 24-41.

The differences between this species and *P. ellioti* are subtle but nevertheless real and very probably sufficient to maintain a distinction at species level. *P. nordenskjoeldi* is a very slightly more slender taxon with a prominent, elevated spire; on some specimens at least there is also a deep, blunt, U-shaped sinus in the upper part of the outer lip. More importantly, the inner lip callus is less prominent and does not project onto the spire. There are indications too, of a more curved columella (e.g. [18], pl. 14, fig. l).

It is clear that there is a slight but distinct stratigraphical separation between the two species, and in the BAS collections *P. nordenskjoeldi* is restricted to Telm 4 (see also [18], fig. 37).

*Perissodonta variabilis* (Wilckens, 1911) [187] (# 7, Fig. 2)

Material: 88 specimens from the following localities: 29-4, 443, 450, 460, 465, 467, 471?, 473, 487, 489, 491, 577, 595?, 630, 1062, 1071-1073, 1082, 1083, & 1092 (Telms 3-6, LMF)([18], p. 106).

Distinctly smaller and thinner-shelled than either *P. ellioti* or *P. nordenskjoeldi* (maximum height = 30 mm), this species has a more bucciniform profile with an elevated spire and expanded, sub-rounded last whorl ([27], text-figs 6 G-K). The ornament comprises approximately 15 elongate, sinuous tubercles which give the early whorls a more quadrate profile than in *P. ellioti* or *P. nordenskjoeldi*. On the body whorl these tubercles are only present on approximately the posterior ⅓ of the whorl, with the anterior portion being occupied by numerous fine, spiral threads. This gives the shell a very distinctive style of ornament.

*Struthiolarella* Steinmann & Wilckens, 1908 [188] should also now be regarded as a subjective synonym of *Perissodonta*) [1].

*Perissodonta shackletoni* (Zinsmeister & Camacho 1980) [27] (# 7, Fig. 2)

Material: Three specimens from localities 10 and 450 (Telm 5, LMF) [18, p. 108].

Originally described by Zinsmeister & Camacho ([27], p. 7) from a single specimen, two more specimens were added by Stilwell & Zinsmeister ([18], p. 108) from locality 450 and the distinction confirmed that *P. shackletoni* was more slender, with a narrower spire and an axial ornament of more rounded nodes rather than the raised tubercles of *P. variabilis*. Nevertheless, these differences are rather subtle and it is currently uncertain as to whether they are such as to be of specific rank ([27], text-fig. 6, A-C, G-K). All the more common species of *Perissodonta* from the La Meseta Formation are characterised by a considerable degree of intra-specific variation.

*Perissodonta steinmanni* (Stilwell & Zinsmeister , 1992) [18] (# 7, Fig. 2)

Material: 19 specimens from localities 443, 445, 456, & 1057 (Telm 5, LMF) ([18], p. 108).

This small – medium (height >27mm) taxon also has the more bucciniform profle and a moderately elevated spire as in *P. shackletoni*. However, in *P. steinmanni* the axially elongated tubercles form a less prominent shoulder and the whorls have an altogether more rounded profile. There is a comparatively large, subovate aperture and the columella bears a moderately thick callus ([18], pl. 13, figs m-p). A distinctive feature of this species is the presence of stronger and more widely spaced spiral ribs, especially on the last whorl.

*Perissodonta laevis* (Wilckens, 1911) [188] (# 7, Fig. 2)

Material: 265+ specimens from the following localities: 2, 3, 16, 19, 27, 29, 464, 478, 491?, 598, 1059, 1061, 1063, 1064, 1073, 1075, 1076, 1096, 1097, 1100, & 1103 (Telms 5-7, LMF) ([18], p. 110).

At first sight this species appears to be close to *P. steinmanni* but it is distinguished by its generally less well developed axial tubercles, especially on the last whorl. It is characterised by numerous fine spiral threads, a broad, shallow posterior sinus, and only a poorly developed callus on the inner lip ([27], text-fig. 6, D-F, L). Originally described by Wilckens [188] as only a variety of *P. variabilis*, it was later established as a distinct species by Zinsmeister & Camacho [27] and this decision was subsequently upheld by Stilwell & Zinsmeister [18].

*Epitonium* *charitopolos* Stilwell & Zinsmeister, 1992 [18] (# 21, Fig. 2)

Material: Six specimens from localities 4, 453 & 1057, Telms 4 & 5, LMF ([18], p. 100).

This medium-sized species is characterised by seven rounded convex whorls and strong, blade-like axial ribs with smooth interspaces ([18], pl. 12, figs g-j). Therefore, it seems correctly referred to *Epitonium*.

*Acirsa antarctodelicatula* (Stilwell & Zinsmeister, 1992) [18] (# 22, Fig. 2)

Material: Five specimens from localities 8, 443, 451 & 453, Telms 3-5, LMF ([18], p. 101).

This material was originally placed into a new genus, *Kallinostrala*, of uncertain familial affinity within the Epitonioidea ([18], p. 101) because of the unusual combination of sinistral shell coiling and weak axial ornament. However, some affinity with the genus *Acirsa* Mörch, 1857 was noted and, on further reflection, this would seem to be a better placement for it ([1], p. 218).

*Acirsa cooki* (Stilwell & Zinsmeister, 1992) [18] (# 22, Fig. 2)

Material: 90 specimens from localities 4, 443, 445, 453, 456, 1057 & 1070? (Telms 4 & 5, LMF) ([18], p. 166).

This is the first of three species originally assigned by Stilwell & Zinsmeister [18] to the pyramidellid genus *Turbonilla* Risso, 1826. However, all three of them are altogether too large to be assigned here and were referred by Beu ([1,] p. 218) to the epitoniid genus *Acirsa*.

*Acirsa aerispira* (Stilwell & Zinsmeister, 1992) [18] (# 22, Fig. 2)

Material: 75 specimens from localities 10, 15, 450, 453, 457, 584, 610 & 1087 (Telms 2-5, LMF) ([18], p. 167).

This species is said to differ from *A. cooki* in having a slenderer shell, more numerous whorls, and generally stronger axial ornament ([18], pl. 24, figs m-t). Nevertheless, as the two species co-occur and both show a reasonable degree of morphological variation, this division into two separate species would repay further careful investigation.

*Acirsa woodburnei* (Stilwell & Zinsmeister, 1992) [18] (# 22, Fig. 2)

Material: Three incomplete specimens from localities 14, 1087? & 1093?, Telms 1(?) – 3, LMF ([18], p. 167).

This is a somewhat larger form which is characterised by axial ribs, deeply incised spiral lines, and irregularly developed varices ([18], pl. 24, figs u & v).

*Taioma bicarinata* Stilwell & Zinsmeister, 1992 [18] (# 7, Fig. 3)

Material: Stilwell & Zinsmeister ([18], p. 137) record approximately 20 specimens from the following localities: 1, 4, 14, 461, 463, 468, 611, 1057 & 1070; Telms 2-5, LMF. Additional BAS specimens: DJ. 77. 84a, 84b, 105, Telm 3 from Cape Wiman region, northern tip of Seymour Island; D9. 212. 3390, Telm 2, NE side of Cross Valley.

This medium – large (typically 60 – 90mm in length) gastropod has a distinctive, broadly fusiform outline, with a comparatively low spire in relation to the maximum diameter. This gives it a spindle shape that is accentuated by a projecting shoulder angulation on the last whorl formed by sharp, deflected tubercles. The aperture is large and sub-diamond shaped and there is a moderately long, tapering and slightly twisted siphonal canal. A strong basal fasciole is present. A second row of tubercles on the last whorl is much less strongly developed and there is an additional ornament of fine, closely spaced spiral cords.

Although the general nature of the shell and pagodaform whorl profile suggest an obvious link to the austral genus *Taioma* Finlay & Marwick, 1937, there are some clear differences from the type species, *T. tricarinata* [148]. The latter is more narrowly fusiform, has more subdued tubercles, and a distinctive tricarinate last whorl. The style of ornament is perhaps closer to the Maastrichtian species *T. charcotiana* (Wilckens), but *T. charcotiana* clearly has a higher spire, and a much more strongly bicarinate last whorl than *T. bicarinata*.

*Taioma*? *antarctocarinata* Stilwell & Zinsmeister, 1992 [18] (# 8, Fig. 3)

Material: Stilwell & Zinsmeister [18] record five specimens from localities 8, 584 and 612; these are all from Telm 2, LMF. Additional BAS specimens: DJ. 77. 82, 99, 100, from Telm 3; D9. 212. 591, 595, 3111-3113, 3120, 3127-3128, 3383, from Telm 2.

The three specimens from locality DJ.77 in the Cape Wiman region are distinctly larger (lengths = 35, 48 & 49mm) and are taken to be adults. All the specimens from locality D9. 212 (Cross Valley) are in the 10 – 25mm range and, like all the Stilwell & Zinsmeiser [18] specimens, are taken to be juveniles. In comparison with *T. bicarinata* this species is smaller, narrower and more distinctly fusiform. The moderately high spire comprises four subquadrate whorls with the angulation accentuated by a series of nodes that tend to be axially extended. Whorls are unicarinate and there is additional ornament of fine, regularly spaced spiral cords. The aperture is elongate – elliptical and siphonostomatous.

Stilwell & Zinsmeister ([18], p. 138) noted a closer resemblance to *T. tricarinata* than *T. bicarinata* but pointed out that there were also affinities to other buccinoidean and perhaps even conoidean taxa. These uncertain taxonomic affinities were confirmed by Beu & Raine [155], and Beu [1] indicated that *T. antarctocarinata* may be better placed within the long-ranging austral genus *Penion* Fischer, 1884.

“*Penion*” n. sp. A (# 18, Fig. 3)

Material: A single specimen, DJ. 77. 102, from Telm 3, LMF in the Cape Wiman region.

This medium – large fusiform specimen has a length of 80mm and maximum width of 33mm. The elongated, pagodaform spire is approximately ⅓ total length and comprises five whorls; it is counterbalanced by a long, slightly curving neck that is almost ½ the total length. Whorl angulation is accentuated by 7-10 sharply projecting and axially extending tubercles. Superimposed on these are traces of strong, regularly spaced spiral cords that cover the teleoconch but appear to be largely missing on the neck. The aperture is rounded – ovate, with a long, gently curving siphonal canal. There are traces of a callus on the inner lip.

The general form of this shell suggests that it can probably be placed within the long-ranging (Danian – Recent) Australasian genus *Penion* Fischer, 1884. It can be compared with the smaller forms of *Penion australocapax* Stilwell & Zinsmeister ([18], pl. 17, figs h-j) but this species is generally larger, broader, and has a proportionally shorter anterior canal.

“*Penion* n. sp. B” (# 19, Fig. 3)

Material: It is clear that the specimens originally identified as *Tudicla doylei* Stilwell & Zinsmeister [18] need to be reassigned. Two of the specimens, the paratypes USNM 441776 (loc. 14, towards the base of Telm 3, LMF) and USNM 44177 (loc. 4, Telm 4) are separated off here as a potential new species of *Penion* Fischer, 1884.

As the holotype and at least two of the paratypes of *Tudicla doylei* lack columellar plaits they cannot belong within the genus *Tudicla* [1]. The former is very probably a fasciolariid but the latter can be provisionally linked to *Penion*. USNM 44176 ([18], pl. 19, figs n & o) shows some affinities with “*Penion*” n. sp. A, but both the illustrated paratypes on the whole are smaller and have a less symmetrical profile. The stratigraphic ranges of “*Penion*” n. sp. A and “*Penion*” n. sp. B overlap and it could be that a more representative collection would show that the two species in fact intergrade.

*Penion australocapax* Stilwell & Zinsmeister, 1992 [18] (# 20, Fig. 3)

Material: 18 specimens from localities 4, 10, 14, 443, 453, 461, 584 & 1057, Telms 2-5, LMF [18, p. 128].

Stilwell & Zinsmeister ([18], p. 128) indicated that most of the 18 specimens are juveniles but the holotype (pl. 17, figs h & j) is quite distinctive and clearly differs from “*Penion*” n. spp. A and B.

*Prosipho stilwelli* Beu, 2009 [1] (# 21, Fig. 3)

Material: Stilwell & Zinsmeister ([18], p. 130) recorded some 36 specimens from the following localities: 8, 15, 584, 597, 1069 & 1070, LMF; these are from Telms 2 – 5, LMF. Approximately 27 BAS specimens were recorded from locality D9. 212 in the mid- to lower levels of Telm 2, Cross Valley: D9. 212. 404-406, 408, 576, 577, 3095-3108, 3116-3118, 3363-3367, 3391.

A small – medium, fusiform shell with heights within the 16 – 24mm range and moderately to well-rounded whorl profiles. Sutures are moderately to deeply impressed and there is a distinctive ornament of strong, widely spaced spiral ribs that bunch anteriorly; approximately 13 of these ribs on the last whorl. Axial ribs present on the early whorls but largely absent on the last whorl. There is a rounded-ovate aperture and short, slightly curved siphonal canal. A number of specimens show a varix-like, thickened outer lip.

Originally assigned to the genus *Aeneator* Finlay, 1927, Beu [1] showed that there was in fact much greater affinity to *Prosipho* Thiele, 1912, a small fusiform buccinoidean with predominantly spiral sculpture [166, 182]. As *Aeneator huttoni* is a junior primary homonym of *Aeneator huttoni* Finlay [189], Beu [1] renamed it *Prosipho stilwelli*. Similarly, both *Aeneator lawsi* and *A. delli* (see below) were transferred by Beu [1]to *Prosipho*.

*Prosipho lawsi* (Stilwell & Zinsmeister, 1992) [18] (# 22, Fig. 3)

Material: Five specimens collected from localities 1057 (mid-Telm 5. LMF) and 1103 (Telm 7) ([18], p. 129).

At first sight this species appears to be close to *P. stilwelli*, but it is slightly larger (holotype length = 28mm) and has more clearly defined and slightly more widely separated spiral cords; these are particularly well defined on the last whorl and the interspaces between them are deeply entrenched. The penultimate and earlier whorls have three equally spaced cords and overall the style of ornament is quite distinctive from that of *P. stilwelli*. The stratigraphic range of *P. lawsi* only partially overlaps with that of *P. stilwelli* (#21, 22, Fig. 3).

*Prosipho delli* (Stilwell & Zinsmeister, 1992) [18] (# 23, Fig. 3)

Material: Two incomplete specimens from locality 584, mid-Telm 2, LMF [18].

This small – medium species (holotype length = 23.5mm) shows strong similarities to both *P. stilwelli* and *P. lawsi* ([18], pl. 17, figs k – o). Nevertheless, it has fewer but more pronounced spiral cords on the last whorl than *P. stilwelli,* and only two more widely spaced cords on the earlier whorls. There is perhaps a closer resemblance to *P. lawsi* but this species clearly has three raised cords on the penultimate whorl. Stilwell & Zinsmeister ([18], p. 129) suggested that *P. delli* may well have been the ancestor of the stratigraphically higher *P. lawsi*. It is clear from the study of modern *Prosipho* species that there are sometimes only subtle differences in shell ornament style between species, e.g. ([182], plates 63 & 64).

*Prosipho polaris* (Stilwell & Zinsmeister, 1992) [18] (# 24, Fig. 3)

Material: 16 specimens from localities 2, 4, 443, 444, 450, 453, 457 & 610 range from Telm 3 to Telm 6, LMF ([18], p. 134). One BAS specimen, D9. 212.3367, occurs somewhat lower in the mid-levels of Telm 2.

A small (14.5 – 18.5mm in height), high-spired, delicate, fusiform shell with moderately to well-rounded whorls; the last whorl is approximately ⅓ total shell height. The aperture is elongate – subovate and a pronounced basal constriction leads into a moderately long, oblique siphonal canal. The shell is covered with very regular and evenly spaced spiral ribs, with approximately 18 being present on the last whorl.

This species was originally referred to *Streptochetus (Streptolathyrus)* Cossmann, 1901 but it is clear from the general form of the shell and style of ornament that it is better placed in *Prosipho*. The delicate, fusiform shell outline and longer siphonal canal separate it from *P. stilwelli,* and the style of ornament from both *P. lawsi* and *P. delli.*

*Prosipho antarctocosta* (Stilwell & Zinsmeister, 1992) [18] (# 25, Fig. 3)

Material: Four specimens from localities 443 and 456, Telm 5, LMF ([18], p. 135).

Although broadly fusiform, this small – medium species (15.0 – 20.0mm in height) has a very distinctive profile formed by seven subquadrate and strongly ornamented whorls. The sculpture comprises sharply raised, widely spaced spiral cords that increase in strength in a posterior direction across each whorl ([18], pl. 18, figs i & j). The aperture is subquadrate – rounded and there is a moderately long and twisted siphonal canal. This style of ornament is very characteristic of several modern *Prosipho* species and there is no doubt that this species belongs within this genus rather than *Neptunea* Röding, 1798 [1].

*Prosipho* n. sp. 1 (# 26, Fig. 3)

Material: Two specimens from BAS locality D9.212 on the northern flank of Cross Valley (low to mid-levels, Telm 2, LMF): D9. 212. 593, 3368.

Two small fusiform specimens measuring approximately 11mm (height) by 8mm (maximum width) and 14mm x 10mm at first sight appear to be very close to *Prosipho stilwelli*. They are of similar fusiform shape and bear traces of the distinctive, regular spiral ornament; both appear to be adults, with the characteristic thickened outer lip. Nevertheless on both these specimens there are traces of axial ornament on the last whorl giving them a rather different cancellate appearance. As both are worn, they could just be variants of *P. stilwelli*, but they are separated out here as a potentially new taxon pending further investigation.

*Prosipho lamesetaensis* (Stilwell & Zinsmeister, 1992) [18] (# 27, Fig. 3)

Material: Three specimens from localities 1061 and 1065, Telm 7, LMF ([18], p. 123).

This species, originally assigned to the muricid genus *Xymene*  Iredale, 1915 (Subfamily Xymene) by Stilwell & Zinsmeister ([18], p.120), is based on three specimens from the topmost levels of the La Meseta Formation. The medium size (height of holotype = 33.0mm; width = 16.5mm), fusiform shape, prominent spiral ornament, and lack of radials readily link this taxon to *Prosipho* rather than *Xymene* [1]. The general form of the shell, wide spacing of the spiral cords, together with their tendency to become more closely spaced anteriorly, distinguish this species from other members of the genus within the LMF.

*Pareuthria hookeri* Stilwell & Zinsmeister, 1992 [18] (# 28, Fig. 3)

Material: Four specimens from localities 10, 444 and 618, Telms 3 - 5 [18]. In addition three specimens bearing the number 4180 and collected from IPS 443 (Telm 5, “west side of the Meseta”) were examined in the A. Grebneff Collection housed in the GNS Science Palaeontological Collections, Lower Hutt, New Zealand.

This is a comparatively small fusiform taxon (height = 11.5 – 18.0mm) with slightly to moderately well rounded whorls; the last whorl comprises more than half the total shell height. The aperture is elongate – subovate, there is a short, recurved, siphonal canal, and well marked fasciole. The shell is almost smooth, but there are faint traces of both spiral and axial threads. The precise status of the three specimens from the A. Grebneff Collection is uncertain. Although similar in form to *P. hookeri*, they have more prominent axial growth lines and one of them bears traces of fine spiral cords toward the anterior end of the last whorl. On balance, they would appear to be closer to *Pareuthria* than *Chlanidota* (as labelled).

This taxon compares well with various modern representatives of *Pareuthria* Strebel, 1905 [166, 182]. A second species assigned to *Pareuthria* by Stilwell & Zinsmeister ([18], p. 123), *P. mccormicki*, is of much less certain affinity. It is clearly very common in Telms 2 and 3 and is said to be distinguishable from *P. hookeri* primarily by a series of axial ribs that fade anteriorly across the whorl ([18], p.123). However, the illustrated specimens are somewhat variable and the assignment to this genus is questionable [1]. It is possible that some of them may be juvenile specimens of a species of *Chlanidota*, which are particularly common in Telm 2.

*Pareuthria* n. sp. 1 (# 29, Fig. 3)

Material: Two specimens from BAS locality D9.212 on the northern flank of Cross Valley (low to mid-levels, Telm 2, LMF): D9. 212. 3354, 3355.

These two specimens both measure approximately 13mm (height) by 9mm (maximum width) and have the general form and appearance of *P. hookeri*. However, they both bear an ornament of fine and very evenly spaced spiral cords, which clearly sets them apart from this species. In addition one of them has a very clearly developed thin parietal callus on the inner margin of the aperture, and traces of this feature can also be seen on the other specimen.

*Chlanidota antarctica* (Wilckens, 1911) [188] (# 30, Fig. 3)

Material: Stilwell & Zinsmeister ([18], p. 131) recorded five specimens from localities 1063 and 1097, Telm 6, LMF. In contrast the BAS material comes from somewhat lower levels, with six specimens originating from locality D9.212 on the northern flank of Cross Valley (low to mid-levels, Telm 2): D9. 212. 544, 3089, 3350-3352, 3385, and over thirty from DJ. 79 in the Cape Wiman region (Telm 3): DJ.79. 313-343. A single specimen from the A. Grebneff Collection (4934) is from undifferentiated LMF.

A small – medium, fusiform taxon with height in the range 15 – 28mm and maximum width of the last whorl 9 – 21mm. The teleoconch consists of 4 – 5 evenly rounded, convex whorls that are separated by deeply impressed sutures; sutural ramps well developed on all whorls. Aperture variable from elongate – oval to oval – rounded; outer lip thin and weakly convex columella with characteristic strong siphonal fold. Siphonal notch broad and dorsally recurved; well developed fasciole present. Ornament comprises narrow, thin but well defined spiral cords. These are generally evenly spaced and approximately equal in width to the interspaces; occasionally less regular. Axial growth lines prominent and occasionally developed into distinct growth pauses. The general form of the ornament is very similar to that of the finer-ribbed varieties of the modern species *Chlanidota (Chlanidota) signeyana* (Powell), e.g. ([190], fig. 9K).

Stilwell & Zinsmeister ([18], p.131) identified this taxon as *Bullia (Buccinanops) antarctica* (Wilckens) but a provisional reassignment to *Chlanidota* [1] can now be confirmed. It was originally assigned to *Nassa nordenskjöldi* Steinmann & Wilckens, var. a*ntarctica* by Wilckens [188]; the taxonomic status of *“Nassa” nordenskjöldi* Steinmann & Wilckens (1908) is currently uncertain.

*Chlanidota tuberosa* (Stilwell & Zinsmeister, 1992) [18] (# 31, Fig. 3)

Material: An extremely common species, with Stilwell & Zinsmeister ([18], p. 126) recording 885 specimens from the following localities: 4, 8, 10, 15, 19, 22, 443, 445, 451, 453, 456, 469, 486, 584, 586, 597, 1057, 1058, 1068, 1069, 1070, 1072, 1081 & 1087 (= Telms 2-6, LMF). Approximately 450 BAS specimens were collected from the following localities: DJ. 77 (Cape Wiman region, Telm 3): DJ. 77. 101, 102a,b, 195, 196, 201; DJ. 81 (Cape Wiman region, Telm 4): DJ. 81.101. D9.212 on the northern flank of Cross Valley (low to mid-levels, Telm 2): D9. 212. 433-543, 583-585, 592, 594, 596, 2942-3090, 3129-3132, 3191-3349, 3353, 3371, 3394-3396. More than 600 specimens are housed in the collections of the Paleontological Research Institute, Ithaca; they come from localities 443 and 453 and have the registration numbers: PRI 59430, PRI 59688, PRI 65223. Nine specimens from the A. Grebneff collection are labelled 4178 and come from locality 443 (Telm 5).

Stilwell & Zinsmeister ([18], p. 126) based their new genus and species, *Austrobuccinum tuberosum*, on a prolific collection of small and small – medium specimens, with the types ranging from 15 – 22mm in height and 11.5 – 15.5mm in maximum width. These specimens have a squat, bucciniform profile with the last whorl typically exceeding well in excess of half the total shell height. The whorls are subquadrate to moderately rounded and are characterised by a distinctive ornament of axially elongated tubercles that fade rapidly in an anterior direction across the whorl. The aperture is elongate – subovate, and there is a short siphonal canal and wide notch. A fasciole is well developed even on the smallest specimens.

Close inspection of the range of material housed in both the BAS and PRI collections strongly suggests that *Austrobuccinum tuberosum* was based on juvenile specimens. Mixed in with them are a series of larger specimens in the 15 – 35mm height range that show a very similar form and style of ornament and in all probability are adults of the same species. The proportions of these larger forms are slightly different in that they have both a more pronounced spire and longer siphonal canal. In two of the largest specimens housed in the PRI there is a pronounced neck and in one of them this is quite strongly recurved. These two large specimens have been informally labelled “*Cominella knobbyformis* n. sp.” but in all likelihood they are just the largest forms of *Austrobuccinum tuberosum*. The distinctive pattern of tubercular ornament is very well preserved on these larger specimens and on some of them this is complemented with a very regular pattern of fine, evenly spaced spiral cords. Traces of these cords can indeed be seen on some of the better-preserved juveniles.

The reassignment of *Austrobuccinum tuberosum* to *Chlanidota* [1] is reinforced by the examination of the larger material. These show a high, oval aperture and a columella with a strong siphonal fold. The tubercular ornament readily distinguishes the species from all other LMF members of the genus.

*Chlanidota antarctohimaleos* (Stilwell & Zinsmeister, 1992) [18] (# 32, Fig. 3)

Material: Stilwell & Zinsmeister ([18], pp.130-131) record 395 specimens from the following localities: 4, 10, 14, 15, 17, 25, 443-445, 447, 450, 452, 453, 456, 457, 462, 467, 469, 470, 473, 486, 487, 596, 610, 615, 618, 1058, 1069, 1072, 1077, 1081-1083, 1085, 1088, 1091, & 1103 (Telms 2-7, LMF).

This is a more elongate – globose form of *Chlanidota* that ranges in height (type material) from 17 – 18mm and width from 13 – 14mm. The spire is depressed and the capacious, inflated last whorl comprises some 80% of the total shell height. The aperture is elongate – subovate and there is a short siphonal canal and well developed fasciole; the illustrated specimens ([18], pl. 17, figs r and t) display a prominent parietal callus. The ornament is quite distinctive in that it comprises flattened, equally spaced spiral ribs that are occasionally slightly irregular in form but always broader than the intervening interspaces. Axial growth lines appear to be only faintly developed and do not significantly deflect the spiral ribs.

Stilwell & Zinsmeister ([18], p.131) originally assigned this species to a new genus, *Sudonassarius*, but in their discussion noted a resemblance to the modern *Chlanidota vestita* Martens. There now seems little doubt that this is in fact a bona fide species of *Chlanidota* [1].

*Chlanidota* ?*antarctohimaleos* (Stilwell & Zinsmeister, 1992) [18] (# 33, Fig. 3)

Material: Specimen D9. 212.3090 from locality D9.212 on the northern flank of Cross Valley (low to mid-levels, Telm 2, LMF).

This single specimen measures 23mm by 15mm but has an incomplete spire. Like *C. antarctohimaleos* it has a more elongate – globose form but in this instance the last whorl comprises only approximately 70% of the total shell height. However, there are traces of the distinctive ornament pattern of closely spaced, flattened spiral ribs and there is a good resemblance to this species. Unfortunately the elongate – ovate aperture is blocked with hard sediment and it is not possible to determine if the parietal callus is present.

Certainly in style of ornament this specimen is closer to *C. antarctohimaleos* than any other species of the genus from the LMF and it is therefore provisionally assigned to this taxon.

*Chlanidota* n. sp. 1 (# 34, Fig. 3)

Material: Two specimens, D9. 212. 3093, 3393 from locality D9.212 on the northern flank of Cross Valley (low to mid-levels, Telm 2, LMF).

These are two even more globose forms of *Chlanidota* measuring 23mm (height) by 18mm (maximum width) and 26mm by 20mm, respectively. In each case the last whorl comprises more than 80% of the total height but the short remaining spire is distinctive in that it terminates in an acute apex, and the three component whorls are sharply defined by well impressed sutures. There are indications of a flat, planispiral protoconch comprising two distinct whorls. The other very obvious feature of these two specimens is that they are nearly completely smooth, with no trace of any spiral ornament and only faint to very occasionally stronger axial growth lines. Both specimens have a distinctive elongate – oval aperture and well defined parietal callus. In both cases the siphonal canal is incomplete but there appears to have been a prominent fasciole.

These two specimens are quite unlike any of the other LMF *Chlanidota* and should be assigned to a new species within the genus.

*Austroficopsis seymourensis* Stilwell & Zinsmeister, 1992 [18] (# 35, Fig. 3)

Material: Stilwell & Zinsmeister ([18], p.116) record 142 specimens from the following localities: 1, 8, 10, 22, 443-445, 456, 457, 469, 584, 586, 597, 610, 612, 1057, 1068-1070, 1081; Telms 2-5, LMF. In addition there are four BAS specimens: DJ. 77.86 (Telm 3, Cape Wiman region), D9.212. 575, 3124, 3133 (Telm 2, on the northern flank of Cross Valley).

A medium- sized (height generally in the 25-35mm range), relatively thin and pyriform taxon with a moderately prominent spire; the latter comprises 3-4 straight-sided whorls. The aperture is elongate and narrow; there is a long and essentially straight siphonal canal, and a weak fasciole. Ornament comprises 10-12 variably developed nodes on the last whorl that create a moderately strongly keeled periphery; these nodes are also present on the penultimate whorl. There is also a prominent ornament of strong, regularly spaced spiral ribs.

The precise taxonomic placement of the genus *Austroficopsis* is uncertain. In their original definition, Stilwell & Zinsmeister ([18], p.115) noted that the lack of reticulate sculpture and presence of a weak fasciole cast some doubt on an assignment within the Ficidae, and suggested that there were also buccinoidean affinities. This view was shared by Beu ([1], p. 217), who indicated that all Stilwell & Zinsmeister’s Ficidae genera (i.e. *Ficus, Ficus (Diconoficus)* and *Austroficus*) could be tentatively transferred to the Buccinoidea.

*Austroficopsis wimani* Stilwell & Zinsmeister, 1992 [18] (# 36, Fig. 3)

Material: Stilwell & Zinsmeister ([18], p.116) record 84 specimens from the following localities: 1, 4, 8, 10, 14, 22, 443, 447, 450, 452, 453, 456, 469, 486, 489, 586, 596?, 611, 630, 1069, 1079, 1091; Telms 2-6, LMF. In addition there are eight BAS specimens: DJ.81.94 (Cape Wiman region, Telm 4), DJ. 252.4 (Cape Wiman region, Telm 5), and locality D9.212 (Telm 2, on the northern flank of Cross Valley): D9. 212. 574, 578, 579, 3094, 3122, 3123.

In almost all respects this species is very close to *A. seymourensis* but is distinguished by its lack of nodes on the last two whorls. As it is also common, and very largely co-occurs with *A.* seymourensis, there is a possibility that the two are end members of one very variable species ([18], p.116), and this possibility should be re-investigated once all the type material has been located.

*Austroficopsis australis* (Stilwell & Zinsmeister, 1992) [18] (# 37, Fig. 3)

Material: A single specimen from locality 4, Telm 4, LMF ([18], p.114).

This single specimen was assigned to a new species by Stilwell & Zinsmeister [18] very largely on the basis of a distinctive style of ornament: in dorsal aspect it displays relatively coarse and evenly spaced spiral cords that are crossed by faint axial growth threads and at least one distinct growth pause. Such an ornament pattern does indeed look distinctive but in almost all other aspects the specimen is very close to the *A. seymourensis – A. wimani* group ([18], pl. 15, figs g,h, j-p).

*Austroficopsis austrinus* (Stilwell & Zinsmeister, 1992) [18] (# 38, Fig. 3)

Material: Nine specimens from the following localities: 10, 19, 443, 456, 469 (Telm 5, LMF) ([18], p.114).

A medium – large shell (height of type material varies from 33-39mm) with a distinctive broad, pyriform shape. It has a significantly lower spire than the *A. seymourensis – A. wimani* group with the sutures barely being discernible. The last whorl is greatly expanded, with a rounded, convex shoulder and an ornament of strong, broad spiral ribs. The aperture is elongate – narrow and the outer lip thin.

Although originally assigned to the subgenus *Ficus* (*Diconoficus*) by Stilwell & Zinsmeister [18], it is now placed, at least temporarily, within *Austroficopsis* [1]. It can be distinguished from *A. seymourensis, A. wimani* and *A. australis* by its broader form, more expanded last whorl, and significantly lower spire. In addition it is considerably larger than at least *A. australis*, from which it also differs in having finer and more closely spaced spiral ribs.

*Austroficopsis meridionalis* (Stilwell & Zinsmeister, 1992) [18] (# 39, Fig. 3)

Material: Two specimens from localities 2 and 597 (Telm 6, LMF) ([18], p.115).

This is another medium – large (31-38mm height), broad, pyriform species with a reduced spire ([18], pl. 15, figs e, f, j). However, the spire on this species is different in that it comprises four small, rounded, convex whorls as opposed to the straight-sided ones of *A. austrinus*. It would also appear to be slightly less inflated than this species.

*Austroficopsis meridionalis* is only known from Telm 6 and Stilwell & Zinsmeister ([18], p.115) have postulated that it may be a direct lineal descendant of *A. austrinus*. This is indeed possible but it has to be borne in mind that both species are known from only limited material and their distinction is perhaps best regarded at present as provisional.

Further study is required to determine if there are indeed five distinct species of *Austroficopsis* within the LMF. It is possible to distinguish two informal groups: the broader and lower-spired *A. meridionalis* and *A. austrinus* on the one hand, and the narrower and higher-spired *A. seymourensis, A. wimani* and *A. australis* on the other. But there is considerable variation within the common species *A. seymourensis* and *A. wimani* and this would repay a careful biometric investigation.

n. gen. *verrucosa* (Stilwell & Zinsmeister, 1992) [18] (# 40, Fig. 3)

Material: Stilwell & Zinsmeister ([18], p. 136) record 129 specimens from the following localities: 1, 4, 8, 14, 453, 465, 472, 532, 584, 611, 1057, 1070, 1087 & 1093 (Telms 1-5, LMF). The position of locality 1093 within Telm 1 may need to be verified; in all other respects Telm 1 has a very distinctive fauna.

This small – medium species (height >39.5mm) has a pronounced bucciniform outline, with the teleoconch comprising 4 – 5 subquadrate – rounded whorls; the last whorl is expanded and tends to be well rounded. There is a large elongate – oval aperture with a variably developed parietal callus and short, oblique siphonal canal. Ornament comprises a combination of axially extended, blunt nodes and spiral cords with somewhat variable spacing.

The presence of the strong abaxially reflected siphonal canal is a particularly diagnostic feature of this form and suggested to Stilwell & Zinsmeister ([18], p. 136) that it should be placed within *Zelandiella* Finlay, 1926. However, this has now been shown to be inappropriate by Beu ([1], p. 217) who pointed out that this genus is characterised by a distinctive callus pad on the neck to the left of the inner lip. Beu [1] also indicated that the holotype and paratype very probably belong within separate genera; the former is a generalised buccinid resembling *Probuccinum* Thiele, 1912 and *Neobuccinum* Smith, 1877, and the latter is closer to *Austrofusus* Kobelt, 1879 ([18], pl. 18, figs o-r). Although tentatively placed by Beu [1] within *Probuccinum*, further reflection suggests that the holotype should now be placed within a new genus; in all probability, establishment of a second new genus will also be necessary for the paratype material.

*Fusinus*? *doylei* (Stilwell & Zinsmeister 1992) [18] (# 45, Fig. 3)

Material: A single specimen, the holotype of *Tudicla doylei* ([18], pl. 19, figs l & m), USNM 441774 from loc. 5. Judging from the locality maps housed in the PRI, Ithaca, loc. 5 occurs at a low level in Telm 5, LMF.

Beu ([1], p. 27) noted that this specimen “belongs in a generalised, elongate fasciolariine genus such as *Fusinus*”.

*Microfulgur byrdi* (Stilwell & Zinsmeister, 1992) [18] (# 46, Fig. 3)

Material: A single specimen, USNM 44178, from loc. 453, Telm 5, LMF. Judging from the locality maps housed in the PRI, Ithaca, loc. 453 occurs at a low level in Telm 5, approximately equivalent to that of loc. 5.

Although Stilwell & Zinsmeister ([18], p.141) compared this specimen with *Tudiclana simulator* Finlay & Marwick, 1937 from the Danian Wangaloan Stage of New Zealand, Beu [1] pointed out that there was in fact a much closer similarity to *Microfulgur longirostris* (Marshall) from the same unit. The latter species is somewhat broader and more regularly ornamented, but the two forms are almost certainly congeneric. *Microfulgur* differs from *Tudiclana* in its taller spire and straighter, more weakly sculptured and more steeply inclined sutural ramp [1].

*Fusinus*? *eonodatus* (Stilwell & Zinsmeister, 1992) [18] (# 47, Fig. 3)

Material: Four specimens from localities 10 and 453, Telm 5, LMF ([18], p. 161).

This is a small – medium (height >36mm), thin, fusiform species with a narrow, pointed spire that roughly balances a long, narrow siphonal canal ([18], pl. 22, figs o, p, s & t). There is an elongate – oval aperture and an ornament pattern of blunt, axially extending nodes and fine, subequally spaced spiral cords. Although assigned to the Pseudomelatomidae (i.e. superfamily Conoidea, *sensu* [191]) genus *Inquisitor* Hedley, 1918 by Stilwell & Zinsmeister ([18], p. 161), it has none of the diagnostic features of this taxon and is very probably a buccinoidean related to *Fusinus* Rafinesque, 1815 ([1], p. 217).

*Fusinus* ? *suraknisos* Stilwell & Zinsmeister, 1992 [18] (# 48, Fig. 3)

Material: Four specimens from localities 6, 443 and 619, Telm 7, LMF ([18], p. 132).

This is a large (>73mm in height) fusiform species with a tall, slender spire and well rounded whorls. Although all the available specimens are worn and incomplete, it is apparent that there are strong axial ribs on each whorl and less prominent, fine spiral ornament. Assignment to the genus *Fusinus* is possible but there are also affinities with *Antarctoneptunea* Dell, 1972 [1].

*Fusinus*? *graciloaustralis* Stilwell & Zinsmeister, 1992 [18] (# 49, Fig. 3)

Material: Two incomplete specimens from locality 14 (Telm 3, LMF) ([18], p. 132).

This is another poorly preserved, fusiform species with a general resemblance to *Fusinus*. Whilst it may well be a distinct species in its own right it might also be just a slightly more nodose variety of *F.*? *suraknisos* ([1], p. 217).

*Trophon radwini* (Stilwell & Zinsmeister, 1992) [18] (# 50, Fig. 3)

Material: Three specimens from localities 586 & 1070, Telm 2, LMF. The species is restricted to the fine-grained siltstone/silty sandstone facies of Telm 2 on the northern flank of Cross Valley dominated by *Leionucula nova* ([18], p.120).

A very distinctive small – medium (height = 26-33mm) broadly pyriform to rapiform species characterised by thick varices on the last whorl separated by deep interspaces. There is a subovate aperture, deep pseudoumbilicus, and short, narrow siphonal canal. Although originally assigned by Stilwell & Zinsmeister ([18], p.117) to the new genus *Caelobassus*, it is better placed within *Trophon* Montfort, 1810 [1]. Merle et al. ([192], p.502, pl. 129, figs 1a, b) treated *Caelobassus* as a subgenus of *Poirieria* Jousseaume, 1880 (Subfamily Muricinae) but the holotype seems to be merely a severely abraded specimen of a *Trophon* species. Recognition of a calcitic outer shell layer in the holotype would confirm a position in *Trophon*, because such a layer is not present in *Poirieria*.

*Eupleura suroabdita* (Stilwell & Zinsmeister, 1992) [18] (# 51, Fig. 3)

Material: A single specimen from locality 630, Telm 6, LMF ([18], p.116).

A medium-sized (height = 26mm) fusiform shell with a prominent spire consisting of five subquadrate whorls. The teleoconch is characterised by strong, subparallel varices and less prominent spiral cords. Although Stilwell & Zinsmeister ([18], p.116) placed this specimen within the Ranellidae, linking it provisionally with *Turritriton* Dall, 1904, Beu [1] pointed out that the dorso-ventrally flattened shape and alignment of the varices to form ridges on opposite sides of the spire indicates a placement within the Muricidae. Details of the aperture and anterior canal are missing but there are affinities to the extant genus *Eupleura* Adams, 1853.

Turbinellidae indet. (# 52, Fig. 3)

Material: Comprises the four taxa: *Mitra (Eumitra) monoplicata* (2 specimens: USNM 441790, 441791)*, M. (E.) sadleri* (7 specimens: including USNM 441792 - 441794)*, ‘Mitra’ thomsoni* (1 specimens: USNM 441797) and *‘M.’ cramei* (2 specimens: USNM 441795, 441796). Total stratigraphic range for these four taxa is from ~220m in Telm 2 to high within Telm 5, LMF [18].

Stilwell & Zinsmeister ([18], pp. 146-149) identified a group of four species from the La Meseta Formation as belonging within the essentially tropical family Mitridae Swainson, 1831. These were each based on a comparatively small number of poorly preserved specimens but it was thought that two could be referred to *Mitra (Eumitra)* Tate, 1889, and two to *Mitra, sensu lato*. Collectively, they comprise a group of small – medium, elongate – fusiform shells with flush to very gently convex whorls and slightly impressed sutures. The apertures appear to be consistently narrow and elongate but the two species of *Mitra (Eumitra)* have only a single columella fold and the two species of “*Mitra*” have two. As such it is much more likely that all four species belong within the Turbinellidae, as Mitridae have three columellar folds, but from the state of preservation it is not possible to be any more specific than this [1].

*Fulgurofusus brecheri* Stilwell & Zinsmeister, 1992 [18] (# 53, Fig. 3)

Material: A single specimen from locality 443, Telm 5, LMF ([18], p.117).

A single medium-sized specimen (height = 19mm) with a distinctive narrow, elongate, fusiform profile and strongly carinate whorls. Each of the four whorls displays a prominent subcentral keel, with the posterior slope being slightly concave. The aperture is subtriangular and there is a long, straight siphonal canal. A link to the Paleocene - Recent turbinellid genus *Fulgurofusus* Grabau, 1904 seems appropriate; but there are clear differences from the New Zealand Wangaloan species *F. vulneratus* Finlay & Marwick, 1937 ([18], p.117).

*Adelomelon fordycei* (Stilwell & Zinsmeister, 1992) [18] (# 55, Fig. 3)

Material: Stilwell & Zinsmeister [18] record 35 specimens of *Eoscaphella fordycei* from localities 1, 5, 14, 453?, 457, 461, 466, 584?, 585, 611, 613, 782?, 1069, 1070, 1085? & 1088. In addition there are 10 specimens of *E. ellioti* from localities 4, 453, 455, 584? & 1057; collectively, these represent a range from low in Telm 2 to high in Telm 5, LMF. BAS material: DJ. 77. 85a, b; DJ. 80. 4; DJ. 81. 91-93; DJ. 252. 5; D9. 212. 581, 582, 3125, Telms 2-4.

*Eoscaphella fordycei* was distinguished by Stilwell & Zinsmeister [18] as a medium to large, fusiform species with a moderately high spire and an ornament pattern of long, widely spaced axial ribs that are strongly projecting at the periphery but then fade sub-centrally along the anterior slope of the whorl. There is a narrow, elliptical aperture and strong fasciole. It was separated from a second, largely coeval species, *E. ellioti*, which is smaller, more slender and has a slightly higher concentration of axial ribs. Nevertheless, when the full range of morphological variation across both species is considered the two species appear to intergrade and it would seem more sensible to combine them within the genotype, *E. fordycei*. In their distinction between the two species, Stilwell & Zinsmeister ([18], pp. 142-144) also emphasised that, whereas *E. ellioti* had three columellar plaits, *E. fordycei* only had two. It was not possible to confirm this distinction with the specimens to hand but in all other respects they seem to be very similar. Beu [1] considered both these taxa to be better placed within the South American genus *Adelomelon* Dall, 1906 [193].

? *Adelomelon suropsilos* (Stilwell & Zinsmeister, 1992) [18] (# 56, Fig. 3)

Material: Six specimens from locs 2, 443, 614? & 619 (Telms 5-6, LMF); one BAS specimen - DJ. 154. 1.

The status of this second species of *Adelomelon* is still uncertain. Stilwell & Zinsmeister ([18], p. 144) described a further medium – large, fusiform volutid which they assigned to the new species, *Pachymelon (Palomelon) suropsilos.* This appears to have slightly less angular whorls and more subdued ornament but the aperture and columella are unknown, and the type specimen is not that different from the holotype of *A. fordycei* ([18], pl. 20, figs a and j). It can certainly be assigned to *Adelomelon* [1], and *Palomelon* Finlay, 1926 is now regarded as a synonym of the restricted endemic New Zealand genus *Alcithoe* H. Adams and A. Adams, 1853. Some indication that this may indeed be a distinct species comes from its stratigraphic position. The localities listed by Stilwell & Zinsmeister [18] are from Telms 5 and 6, and in the text (p. 145) they also hint at rare occurrence in Telm 7. The BAS locality DJ. 154 is high Telm 5 or Telm 6.

*Odontocymbiola amundensi* (Stilwell & Zinsmeister, 1992) [18] (# 57, Fig. 3)

Material: Single specimen, USNM 44178, known from locality 453, Telm 5, LMF [18].

A further large (length = 106 mm) volutid was referred by Stilwell & Zinsmeister ([18], pl. 21, figs a and b) to *Alcithoe (Alcithoe) amundseni*. This specimen has a stoutly fusiform shell comprising six subquadrate whorls. The greatly expanded last whorl has a series of widely spaced nodes at the periphery but is otherwise smooth. There is an expanded outer lip but the columella is not preserved. Beu [1] suggested that such a distinctive shape indicated an affinity with *Pachycymbiola* Ihering, von, 1907 but was perhaps best assigned to the genus *Odontocymbiola* Clench & Turner, 1964 [193].

*Miomelon antarctica* (Stilwell & Zinsmeister, 1992) [18] (# 58, Fig. 3)

Material: Known from only a single specimen, USNM 441789 from loc. 1094, which occurs at a mid- to low level in Telm 2 in the Cape Wiman region (and thus is referred to a mid- to low level in the same unit in the composite range chart).

A small – medium (length = 33.5mm), elongate – fusiform volutid with a prominent shoulder giving the whorl a squarish profile ([18], pl. 20, fig. i). There is a strong ornament of widely spaced axial ribs, which are separated by incised interspaces. The columella possesses three distinct anterior plaits. Although Stilwell & Zinsmeister [18] placed this specimen in the genus *Lyria* Gray, 1847, this genus has much more numerous columellar plaits, and Beu [1] believed the arrangement of columella plaits and general form to be closer to *Miomelon* Dall, 1907, with *Palaeomelon* Nielsen & Frassinetti, 2007 [185] being a possible alternative.

*Tractolira* n. sp. (# 59, Fig. 3)

Material: 10 BAS specimens – D9. 212. 580, 2935-2941, 3380-3382 – from Telm 2, LMF, Cross Valley region. They are judged to have come from the mid-low levels in Telm 2 in the composite section.

Small – medium, slender, fusiform shells, the largest of which measure approximately 55mm (length) by 20mm (width) and 50mm by 23mm; in comparison the smallest shell is approximately 28mm by 14mm. Even though most of the specimens are incomplete with some or all of the juvenile whorls missing, it is clear that this is a distinctive volutid with the teleoconch comprising usually five only slightly convex whorls and a distinctly impressed suture. The elongate-oval aperture has a height equal to 50% or more of the total shell height and there are at least two distinct anterior columella plaits. There is an axial sculpture of very fine growth threads and, in places, traces of cross-cutting spiral threads and thus a faint cancellate structure. These specimens invite immediate comparison with modern specimens of *Tractolira* such as *T. germonae* Harasewych, 1987 and in particular *T. delli* [194] (see also [182], pl. 70, figs 7 and 8). This would appear to be the first occurrence of the genus in the fossil record and represents a new species.

*Volutomitra*? *cernohorskyi* (Stilwell & Zinsmeister, 1992) [18] (# 60, Fig. 3)

Material: Stilwell & Zinsmeister ([18], p.150) listed six specimens from six different localities: 4, 443, 456, 611, 1068 & 1070, Telms 2-5, LMF. In contrast the BAS material comprises approximately 45 specimens from just one locality at mid- to low levels in Telm 2, Cross Valley: D9. 212. 407, 489-492, 547, 586-588, 597, 3134-3163, 3165, 3369-3370 & 3392.

A small elongate-fusiform to biconic shell with lengths typically in the 12 – 15mm range and widths of 6 – 8mm. A moderate spire typically comprises 4-5 subquadrate to very slightly rounded whorls and the large last whorl is very slightly inflated. Shell smooth with a narrow, elongate aperture; there is a short, spout-shaped siphonal canal and the columella has four distinct but irregularly spaced plaits. Although undoubtedly volutomitrids, these specimens are probably better assigned to *Volutomitra* H. & A. Adams, 1853 than *Conomitra* Conrad, 1865. This is the more slender and higher-spired form of *Volutomitra*? to occur in the La Meseta Formation and is quite distinct from *V*.? *iredalei* (see below). It is likely that the comparatively rare *Volutomitra*? *antarctolirata* [18] is only a variant of *V*.? *cernohorskyi* with a lirate labrum.

*Volutomitra*? *iredalei* (Stilwell & Zinsmeister, 1992) [18] (# 62, Fig. 3)

Material: Stilwell & Zinsmeister ([18], p. 150) list some 30 specimens from a range of localities within Telms 2-5, LMF: 4, 10, 14, 443, 445, 447, 451, 453, 581, 610, 611, 618 & 1086. The single BAS specimen, DJ. 77.203, comes from a locality in the Cape Wiman region (Telm 3).

The single BAS specimen is in an excellent state of preservation and demonstrates the clear differences between this and the preceding species. This is a squatter, subovate form with a much shorter spire and a last whorl equal in length to ¾ total shell length. Whereas the two more posterior columella plaits are approximately perpendicular to the shell axis, the two anterior ones are much more oblique. Fine growth lines can be made out on the shell surface but there is no other form of ornament.

*Zemacies finlayi* Stilwell & Zinsmeister, 1992 [18] (# 65, Fig. 3)

Material: 19 specimens from the following localities: 2, 22, 29, 460, 471, 489, 577, 1058, 1071, 1072, 1092, 1100 & 1103 (Telms 5-7, LMF) ([18], p. 155).

A small – medium (type material ranges in height, 22.5 – 44.5mm) moderately solid, turriform taxon with strongly carinate whorls [18, pl. 22, figs w-y]. There is an ornament of fine, closely spaced spiral threads, long siphonal canal and moderately deep anal sinus. This conoidean genus is now assigned to the family Borsoniidae [191].

*Aforia*? *canalomos* (Stilwell & Zinsmeister, 1992) [18] (# 66, Fig. 3)

Material: Six specimens from localities 623, 1076 and 1097 (Telms 6 & 7, LMF) ([18], p. 154).

This species bears a general resemblance to *Zemacies finlayi* but is distinguished by a moderately deep canaliculated spiral groove on the shoulder ([18], pl. 22, figs u and v). However, its sculpture resembles that of some Recent Antarctic species belonging to *Aforia* Dall, 1889, and it may be better assigned there ([1], p. 217).

*Marshallaria*? *oliveroi* (Stilwell & Zinsmeister, 1992) [18] (# 67, Fig. 3)

Material: 156 specimens from the following localities: 4, 8, 10, 14, 15, 45, 443-445, 450, 453, 454, 456, 457, 478, 532, 584, 594?, 597, 612, 1057, 1068, 1070, 1087, & 1094 (Telm 2 – lowermost Telm 6, LMF) ([17], p. 155); D9. 212. 3113, 3359 (Telm 2, on the northern flank of Cross Valley).

A small – medium (>38mm height) fusiform shell with the periphery of subquadrate whorls keeled by prominent, rounded tubercles. There are also subequally spaced spiral cords, and the slightly concave posterior face of each whorl bears traces of a shallow posterior sinus. There is an obliquely elongated aperture, short, wide siphonal canal, and indications of a distinct fasciole.

From its inception it has proved difficult to decide whether this species should be assigned to either *Austrotoma* Finlay, 1924 or *Marshallaria* Finlay & Marwick, 1937 ([18], pp. 155-156). Whereas the apparent lack of a distinct siphonal notch and presence of a canaliculated suture originally suggested affiliation to the former of these genera, the presence of a relatively long, narrow anterior canal now indicates greater allegiance to the latter ([1], p. 218).

*Austrotoma* n. sp. (# 68, Fig. 3)

Material: Five BAS specimens: DJ. 79. 343 (Telm 3, LMF, Cape Wiman region); DJ. 81. 95 (Telm 4, Cape Wiman region); D9. 212. 589, 3119, 3373 (Telm 2, on the northern flank of Cross Valley).

A small – medium (height = 20 – 30mm), fusiform taxon, with the narrow, pointed spire equal to approximately half the total height of the shell. The teleoconch comprises 4-5 shouldered whorls with a distinctive concave sutural ramp; subsutural folds are weakly developed on early whorls. Axial sculpture comprises variably developed opisthocline costae extending from the shoulder angulation to mid-whorl initially but becoming obsolete on later whorls; 16-18 per whorl. Numerous well defined growth lines cover the whole surface on the best preserved specimens (DJ. 79. 343; DJ. 81. 95). The spiral sculpture comprises traces of fine threads on the ramp, and narrow, well defined cords below. The cords are regularly and evenly spaced, and on the specimen with the strongest growth lines (DJ. 81. 95) form a distinctive, crenulated pattern. The aperture is elongate – subrectangular, and there is a short, deeply notched siphonal canal; fasciole variably developed.

The general form of the shell and presence of finer spiral threads on the shoulder as opposed to coarser cords on the rest of the whorl link this taxon firmly to *Austrotoma* Finlay, 1924 [148, 155, 195]. In addition the presence of comparatively slender, opisthocline costae as opposed to more clearly rounded tubercles distinguish this species from *Marshallaria*? *oliveroi* (Stilwell & Zinsmeister). *Austrotoma ventricosa* Stilwell & Zinsmeister [18] (see below) is an altogether larger shell. The five specimens described here should be assigned to a new species.

*Austrotoma*? v*entricosa* Stilwell & Zinsmeister, 1992 [18] (# 69, Fig. 3)

Material: Five specimens from locality 14 [18] and one BAS specimen, DJ. 77.83. All the material comes from Telm 3, LMF at the northern end of Seymour Island.

A larger (height > 51.5mm), more robust form with a stoutly fusiform outline and spire that is approximately equal to half the shell height. The teleoconch comprises five whorls that are separated by slightly canaliculate sutures and subsutural swellings that are followed by a sulcate ramp ([18], pl. 23, figs f-i). The last whorl displays an angulate outer periphery of 16 blunt tubercles that are axially extended across the whorl. Fine axial growth lines are variably developed and a regular ornament of fine spiral cords is best seen on the anterior flank of the last whorl. Both axial and spiral ornament is less well developed on the earlier whorls. The aperture is elongate – suboval, there is a short, wide siphonal canal, and a prominent fasciole.

Although this distinctive larger conoidean bears obvious affinities to *Austrotoma* Finlay, 1924, there are still some grounds for making this only a tentative assignment at the moment (discussed at length in Beu [195]). On size and shape grounds alone, this taxon can be easily separated from all the other LMF conoideans.

*Austrosullivania lata* Stilwell & Zinsmeister, 1992 [18] (# 70, Fig. 3)

Material: 125 specimens from the following localities – 8, 443, 445, 447?, 456, 584, 586, 597, 612, 1068 (Telms 2-5, LMF) ([18], p. 159); BAS specimen D9. 212. 3360 (Telm 2, on the northern flank of Cross Valley).

A small – medium (height >21mm) elongate – biconical species with a high spire comprising 7-8 straight-sided to very slightly convex whorls. The suture is moderately impressed to canaliculated and there are faint traces of a very shallow, arcuate sinus. There is no appreciable axial ornament and spiral threads are only very faintly developed. The elongate – oval aperture leads into a moderately long and oblique siphonal canal ([18], pl. 23, figs m-p).

*Austrosullivania striata* Stilwell & Zinsmeister, 1992 [18] (# 71, Fig. 3)

Material: 32 specimens from the following localities – 4, 15, 453, 946?, 1057 (Telms 4 and 5, LMF) ([18], p. 158).

A narrower form of the Antarctic genus *Austrosullivania,* with a slightly higher spire, more convex whorls, and smaller last whorl ([18], pl. 23, figs j-l).

*Gemmula askinae* Stilwell & Zinsmeister, 1992 [18] (# 72, Fig. 3)

Material: Four specimens from locality 1087, Telm 2, LMF ([18], p.160).

A small (height = 10-13mm), turriculate species with distinctive carinate whorls. The last whorl is bicarinate with a strong posterior keel comprising 16 well defined gemmules, and there is a less well defined anterior one ([18], pl. 22, figs m, n). The aperture is elongate – pyriform, there is a moderately long, twisted siphonal canal, and a smooth labrum.

The four specimens come from a single locality within Telm 2 at the northern end of Seymour Island ([18], p.160). This makes them approximately Early – Middle Eocene in age and thus roughly equivalent to the probable first records of the genus in New Zealand [150]. Although the genus is widespread in Indo-Pacific warm waters at the present day [196], the palaeoclimatic significance of its occurrence in the Eocene of Antarctica has yet to be fully assessed.

*Spirotropis*? n. sp. (# 73, Fig. 3)

Material: Seven rather worn and incomplete specimens from locality D9. 212 (Telm 2, LMF, on the northern flank of Cross Valley): D9. 212. 488, 3357, 3361, 3362, 3374 – 3376.

A small – medium (height >18.5mm), slender, spindle-shaped form with 5-6 teleoconch whorls that are slightly to moderately strongly carinate. Suture deeply impressed to canaliculated and in the more abraded forms the early whorls appear more sub-rounded. Specimen D9. 212. 3374 bears faint traces of strongly prosogyrous axial ribs that are clearest across the carina region of the last whorl; on specimen D9. 212. 3351 the carina is more inflated and there are traces of coarser axial ribbing without any obvious flexure. The last whorl also bears occasional fine spiral cords; the aperture is elongate – oval, and there are traces of a short, broad siphonal canal.

The slender carinate form of these shells and style of ornament bears a strong resemblance to modern members of the genus *Spirotropsis* G.O. Sars, 1878, and in particular to taxa such as *S. patagonica* (d’Orbigny, 1841) from southern Argentina and *S. studeriana* (von Martens, 1873) from the Falkland Islands, e.g. [197]. Collection of better preserved material would be helpful to confirm this generic identification.

*Typhlomangelia*? n. sp. (# 74, Fig. 3)

Material: A single specimen, D9. 212. 3114, from Telm 2, LMF, on the northern flank of Cross Valley.

This small (height = ~12mm; maximum width = 6mm) incomplete specimen has a fusiform profile and narrow, acute spire. The teleoconch comprises four whorls and there is a distinctive protoconch of three plain, inflated whorls. The suture is quite strongly impressed and there is a characteristic ornament of raised, ridge-like nodes positioned sub-centrally on each whorl; approximately 12 nodes per whorl. The last whorl is expanded, there are indications of a comparatively narrow neck, but the siphonal canal is incomplete. Thin, regular spiral cords can be traced on the neck and there are indications that they are present on earlier whorls too.

This small conoidean with a very distinctive style of ornament bears a strong resemblance to the modern Antarctic species *Typhlomangelia* *principalis* Thiele, 1912, e.g. ([196], p.82, pl. 12, figs 16, 17). However, the correspondence is not exact and it is noticeable how close comparisons can also be made with other representatives of this widespread genus, such as *Typhlomangelia* sp. from the Solomon Islands ([191], fig. 2A). It should be formally assigned to a new species in due course.

*Agladrillia*? n. sp. (# 75, Fig. 3)

Material: A single specimen, D9. 212. 3115, from Telm 2, LMF, on the northern flank of Cross Valley.

This small – medium (height ~15mm, maximum width = 6mm) shell is clavate – fusiform, with a quite strongly constricted base. The teleoconch comprises ~4½ moderately convex whorls that are strongly impressed below the suture. Protoconch missing and aperture region also incomplete; there are indications that it was narrowly pyriform and led into a relatively long and narrow siphonal canal. Tip of the latter missing? Narrow, ridge-like and strongly opisthocline axial ribs developed on all whorls; terminating at the shoulder and on the last whorl reaching upper part of the rostrum. Approximately 12 ribs per whorl, separated by relatively broad interspaces. No spiral ornament detectable; traces of a moderately deep sinus on the shoulder of the last whorl.

This single specimen compares very well with the modern genus *Agladrillia* Woodring, 1928. Although essentially American in its distribution, it has also been recorded from both South Africa [198] and southern South America. Indeed, there are quite strong resemblances to *A. fuegensis* (Smith) from southern Argentina ([197], fig. 316).

*Makiyamaia*? n. sp. (# 76, Fig. 3)

Material: A single specimen, D9. 212. A, from Telm 2, LMF, on the northern flank of Cross Valley.

This small – medium species (height ~20mm; maximum width = 12mm) has a distinctive pagodaform profile with rather angulate whorls accentuated at the periphery by prominent nodes. The teleoconch comprises four whorls, with a moderately elongate spire and elongate - pyriform aperture; unfortunately the anterior canal is incomplete. A tilted, smooth, planispiral protoconch is well preserved and the primary ornament comprises a series of prominent, widely spaced nodes; there are 10 of these on the last whorl where they have a marked papillate appearance. There are numerous fine axial growth lines and traces of a comparatively shallow posterior sinus on the shoulder of the last whorl.

This unique specimen shows its closest taxonomic affinities to the Neogene – Recent Indo-Pacific genus *Makiyamaia* MacNeil, 1960.

? *Splendrillia antarctoliqua* Stilwell & Zinsmeister, 1992 [18] (#77, Fig. 3)

Material: A single specimen from locality 453, Telm 5, LMF ([18], p. 160).

This distinctive small, turriform specimen with prominent axial sculpture was referred to *Splendrillia*, *sensu stricto*, by Stilwell & Zinsmeister [18], but it should be noted that the genus is now classified within the Family Drilliidae [191]. This would appear to be the earliest fossil record of a genus that is widespread at the present day in the Indo-Pacific region [http://paleobiodb.org].

? *Cochlespira brychiosinus* (Stilwell & Zinsmeister, 1992) [18] (#78, Fig. 3)

Material: Nine specimens from localities 14, 453, 1055 & 1057, Telms 3-7, LMF ([18], p. 159).

A small form with a pagodaform spire comprising six concave whorls. These whorls display a prominent keel ornamented with a moniliform cord; well-defined spiral ribs on the anterior portion of the final whorl, and a comparatively long, curved siphonal canal ( [18], p. 159).

*Pristimercia australis* Stilwell & Zinsmeister, 1992 [18] (# 79, Fig. 3)

Material: Two specimens, possibly one adult and one juvenile, from localities 26 and 1057, Telms 3-5, LMF ([18], p. 151).

The two specimens assigned to this species by Stilwell & Zinsmeister [18] are rather different in both size and style of ornament. The larger of them, the holotype (USNM 441806), is medium-sized (height = 24mm; width = 20mm), fusiform, with a relatively short spire and subquadrate to rounded whorls. The suture is moderately deeply impressed, the last whorl well inflated and the aperture elongate – subovate; there is a short, broad siphonal canal. A characteristic style of ornament comprises coarse, widely and evenly spaced spiral cords and only the faintest traces of axial growth lines. The outer lip displays a finely lirate labrum and there are two central folds on the columella ([18], pp. 151-152). Overall this specimen shows a strong resemblance to the Wangaloan (Danian) genus *Pristimercia* Finlay & Marwick, 1937 from New Zealand, and the genotype, *P. dolioides* Finlay & Marwick ([148], pl. XI, figs 2 & 3).

Nevertheless, the paratype (USNM 441807) is somewhat different in form and may be closer to a second Wangaloan cancellariid genus from New Zealand, *Coptostomella* Finlay & Marwick, 1937. Stilwell & Zinsmeister [18] separated it off from their new La Meseta Formation species *Coptostomella*? *notopolaris* (Stilwell & Zinsmeister, 1992) largely on the style of ornament, noting that the spiral ribs were consistently more widely spaced. They thought that it most likely represented a juvenile *P. australis*, but this view may need to be substantiated by further study (see below).

*Coptostomella*? *notopolaris* (Stilwell & Zinsmeister, 1992) [18] (# 80, Fig. 3)

Material: Stilwell & Zinsmeister [18] record 14 specimens from localities 4?, 10, 447, 453, 457?, 581 and 586 (Telms 3-5, LMF).

This is a smaller (height of type material = 10-13.5mm), more slender, bucciniform taxon that bears a distinctive ornament of fine, closely spaced spiral cords. It clearly cannot be placed in *Pristimercia* and Stilwell & Zinsmeister ([18], pp. 152-154) thought that the best fit was with the Northern Hemisphere genus *Coptostoma* Cossman, 1899. This could be correct but until a more thorough review of Southern Hemisphere Paleogene cancellariids has been carried out it is tentatively referred here to the New Zealand *Coptostomella* Finlay & Marwick [1].

*Acteon inflatus* (Stilwell & Zinsmeister, 1992) [18] (# 23, Fig. 2)

Material: One specimen from locality 577, Telm 5, LMF [18, p. 163].

This specimen is also thought to be too large to be a pyramidellid and is more likely linked to *Acteon* Monfort, 1810 ([1], p. 218).

*Neacteonina eoantarctica* Stilwell & Zinsmeister, 1992 [18] (# 24, Fig. 2)

Material: Seven specimens from localities 10, 447, 451?, 610 & 613, Telms 3-5, LMF ([18], p. 168).

The greatly inflated, capacious last whorl is ornamented with ~25 spiral cords of variable distinction ([18], pl. 24, figs w & x). The genus is distinguished from the similar *Tornatellaea* Conrad, 1860 by the presence of a single, weak columella fold but it is more likely assignable to *Neactaeonina* Thiele, 1912 than *Acteon* Montfort, 1810 ([1], p. 218). Indeed, there is considerable similarity to the modern *Neacteonina edentula* (Watson, 1883) from Antarctica ([166], p. 248, figs 439, 440).

*Tornatellaea darwini* Stilwell & Zinsmeister, 1992 [18] (# 25, Fig. 2)

Material: Nine specimens from localities 1, 10, 445, 453, 597, 610, Telms 3-5, LMF ([18], p. 168).

The genus *Tornatellaea* is easily distinguished from both *Acteon* Montfort, 1810 and *Ringicula* Deshayes, 1838 by its two well separated columella folds ([18], p. 168).

*Kaitoa duseni* (Stilwell & Zinsmeister, 1992) [18] (# 26, Fig. 2)

Material: 110+ specimens from the following localities: 10, 14, 443, 445, 453, 456, 590, 597 & 1087, Telms 2-5. LMF ([18], p. 172). In addition 11 BAS specimens from the following localities: DJ. 77. 199 (Telm 3), DJ. 79. 344 (Telm 3), DJ. 81. 96-98, 102, DJ. 252. 1-3 (Telm 5), D9. 212. 573, 3121 (Telm 2).

In almost all respects the BAS material closely resembles the description given by Stilwell & Zinsmeister ([18], p. 172, pl. 25, figs d-f): the shell is ovate – oblong, with the apex covered by the outer lip, and the inflated last whorl covered by numerous, incised spiral lines separated by flattened interspaces. However, these authors apparently had only small specimens at their disposal, with the type material varying between 6mm and 10mm in height. Six of the BAS specimens are considerably larger, measuring 20 – 26mm in height and 12 – 17mm in maximum width. *Priscaphander schmitti* ([18], pl. 25, figs l-o) is larger but it lacks the distinctive spiral ornament and is apparently restricted to Telms 6 and 7. *“Kaitoa” antarctidis* [185] ([18], pl. 25, figs j,k) has an apically extended outer lip and is almost certainly not a scaphandrid; it has been tentatively reassigned to the haminoeid genus *Limulatys* Iredale, 1936 ([1], p. 218).

*Leionucula nova* (Wilckens, 1911) [188] (# 27, Fig. 2)

Material: 147 specimens from the following WJZ localities: 8, 10, 20, 443, 451, 453, 577, 584, 586, 610, 612, 613, 1068-1070, 1087, 1097 (Telms 2-5, LMF) ([18], p. 47); 1770 BAS specimens from locality D9. 212 (Telm 2, on the northern flank of Cross Valley): D9. 212. 1-242, 616-2145.

This prolific nuculid is characteristic of the mud-rich facies within Telm 2 of the Cross Valley region but can also occur as high as Telm 5 where Stilwell & Zinsmeister ([18], p. 47) have suggested that it has been washed in from “nearby lower-energy facies”. The relatively small size, sub-trigonal outline, well rounded ventral margin, and distinctive ornament of fine commarginal growth lines and distinct pauses readily distinguish this species from the Paleocene *oblonga/suboblonga/hunickeni* group. The only taxonomic uncertainty surrounding this species is its relationship to *L. palmeri* [199] (see below).

*Leionucula palmeri* (Zinsmeister, 1984) [199] (# 27, Fig. 2)

Material: 14 specimens from localities 1, 14, 597 (Telm 3, LMF) ([18], p. 48).

This much smaller form was originally established by Zinsmeister ([199], pp. 1501-1502) using a series of characters the most important of which appears to be the possession of fine, regular commarginal ornament. This may indeed be a character of specific rank but it is noticeable how the general standard of preservation in Telm 3 is significantly better than that in Telm 2. There are traces of more regular ornament on some specimens of *L. nova* and this difference between the two taxa needs further careful investigation.

*Yoldia (Aequiyoldia) antarctica* (Zinsmeister, 1984) [199] (# 28, Fig. 2)

Material: Two specimens from localities 10 and 443, Telm 5, LMF ([18], p. 50).

The general form of this species, with its subtrigonal outline, subcentral umbones that do not project above the hingeline, and only weakly defined rostrum, is very reminiscent of the widespread modern Antarctic species *Yoldia (Aequiyoldia) eightsi* (Couthouy, in Jay, 1839) ([1], p. 215). This medium-sized species (holotype = 24.0mm in length, 17.0mm in height) clearly also shows a broad, shallow resilifer, an unequal number of anterior and posterior teeth, and a moderately deep, rounded pallial sinus ([18], pl. 1, figs g & h).

*Yoldia (Aequiyoldia) maxwelli* (Stilwell & Zinsmeister, 1992) [18] (# 28, Fig. 2)

Material: A single disarticulated specimen of two worn valves from locality 591, Telm 3 ([18], p. 52).

This specimen can also be referred to *Yoldia (Aequiyoldia) (*[1], p. 215), but it is noticeably longer (length = 37mm) and thicker-shelled than *Y. (A.) antarctica* ([18], pl. 1, fig. i). In the illustrated left valve the umbo has a more anterior position and there is a long, straight postero-dorsal margin. Both this species and *Y. (A.) antarctica* are rare in the LMF.

*Cyclocardia mesembria* (Stilwell & Zinsmeister, 1992) [18] (# 31, Fig. 2)

Material: Five specimens, one from each of the following localities: 443, 450, 491, 630, 1096; Telms 5 and 6, LMF ([18], p. 70).

This is a medium-sized (length of holotype = 18.5mm; height = 17.5mm), rounded – subquadrate form that is slightly to moderately inflated and has a distinctive crenulated margin ([17], pl. 5, figs q, r). The illustrated right valve holotype shows a typical *Cyclocardia* dentition, with two prominent cardinals (3a, 3b) and a long, narrow postero-lateral that is sub-parallel to the postero-dorsal margin. Ornament comprises approximately 21 broad, rounded radial ribs that are separated by well defined interspaces that increase slightly in width towards the ventral margin. These radial ribs are crossed by numerous fine, commarginal growth lines, some of which are stronger than others.

*Cyclocardia* n. sp. 1 (# 31, Fig. 2)

Material: A single BAS specimen, D9. 208. 283, from Telm 1, LMF; locality D9. 208 is a small exposure approximately 500m to the south-west of Bill Hill on the westernmost extremity of the LMF area of outcrop.

This well preserved right valve is of similar dimensions to the holotype of *C. mesembria* (i.e. length = 17.0mm, height = 15.0mm) but slightly more asymmetrical and prosogyrous in form. There is a more rounded - rectangular outline, slightly longer and straighter postero-dorsal margin, and the radial ribs are very slightly straighter and more closely packed together. There are again traces of very fine commarginal growth lines and several prominent growth pauses towards the ventral margin. Another important difference is in the form of the RV dentition where the posterior cardinal (i.e. 3b) is narrower and straighter, and set at a significantly steeper angle to the prominent postero-lateral tooth. This is almost certainly a distinct species from *C. mesembria*.

*Parathyasira notosulca* (Stilwell & Zinsmeister, 1992) [18] (# 32, Fig. 2)

Material: Nine specimens from localities 490 and 491, Telm 5, LMF ([18], p. 65).

This is a medium – large species with the holotype measuring 19.0mm in length and 21.0mm in height. It has an erect, rounded – subangular profile with strongly prosogyrous but inconspicuous beaks ([18], pl. 4, figs k-m). The posterodorsal sulcus is less steeply impreseed than in *P. austrosulca* and defines a more rounded posterodorsal shell margin. The ventral margin is again well rounded but the anterior margin is more angular than in *P. austrosulca*.

*Mysella trigonoelliptica* Stilwell & Zinsmeister, 1992 [18] (# 34, Fig. 2)

Material: Nine specimens from locality 443, a single horizon within Telm 5, LMF ([18], p. 66).

This is a minute species measuring approximately 2mm in both height and length. The left valve shows a distinctive arrangement of cardinal teeth but the laterals are easily differentiated ([18], pl. 5, a-c).

*Gaimardia zinsmeisteri* Beu, 2009 [1] (# 35, Fig. 2)

Material: 51 specimens from localities 8, 14, 447, 600, 615? & 1091, Telms 2 & 3, LMF ([18], p. 68).

This small – medium species (length of holotype = 15mm, height = 12mm) is almost completely smooth, has a distinctive rounded – trapezoidal outline, and evidence of reduced cardinal and lateral dentition ([18], pl. 5, figs d – h).

*Gaimardia zinsmeisteri* Beu, 2009 is a replacement name for *G. flemingi* Zinsmeister (1984), a junior primary homonym of *G. trapesina flemingi* Powell, 1955 ([1], p. 215).

*Cyamiomactra truncilla* (Stilwell & Zinsmeister, 1992) [18] (# 36, Fig. 2)

Material: 150+ specimens from localities 1077 & 1091, Telms 2 & 3, LMF ([18], pp. 68-69).

This is a small (holotype 5.0mm length by 4.0mm height), rounded-rectangular species displaying a moderate degree of inflation and only the very faintest traces of both radial and commarginal ornament ([18], pl. 5, figs i-k). Originally assigned by Stilwell & Zinsmeister [18] to the genus *Anisodonta* Deshayes, 1858 within the family Sportellidae, it may in fact be better placed within *Cyamiomactra* Bernard, 1897 within the Cyamiidae. As Beu ([1], p. 215) has pointed out, there are considerable similarities between *C. truncilla* and the modern Antarctic species, *C. laminifera* (Lamy) ([166], fig. 100).

*Cyamiomactra subovata* (Stilwell & Zinsmeister, 1992) [18] (# 36, Fig. 2)

Material: Three specimens from locality 1062, Telm 7, LMF ([18], p. 69).

This species is of similar size to *C. truncilla* but is distinguished by its more ovate outline, more compressed shell, and greatly elongated anterodorsal margin ([18], pl. 5, figs l-n). It is known only from Telm 7 at the top of the LMF.

*Hiatella*? *arctica* (Linné) (# 37, Fig. 2)

Material: 172 specimens from the following localities: 2, 17, 464, 471, 478, 491, 492, 625, 630, 1059, 1061, 1062, 1071-1073, 1075, 1076, 1092, 1096, 1097, 1100, 1101 & 1103 (Telms 5-7, LMF) ([18], p. 88).

This is a medium-sized species (hypotype 31.0mm length by 16.0mm height) with a distinctive trapezoidal outline and well developed posterodorsal furrow. There is a variably developed ornament of irregularly spaced commarginal ribs. Originally referred to *Hiatella tenuis* [189] by both Zinsmeister [199] and Stilwell & Zinsmeister [18], it may be better placed within the ubiquitous *H. arctica* (Linné, 1767) ([1], p. 210).

*Thracia topei* (Stilwell & Zinsmeister, 1992) [18] (# 38, Fig. 2)

Material: 83 WJZ specimens from the following localities: 1, 2?, 3, 8, 14, 443, 447, 451, 454, 462, 464?, 492?, 577, 586, 591, 600, 615, 616, 624?, 1061, 1062, 1064?, 1067?, 1071?, 1076?, 1088, 1097?, 1100 & 1103? (Telms 2-5, LMF) ([18], p. 89). BAS material: D9. 212. 609, 2917-2921, 2886, mid- to low levels in Telm 2, Cross Valley.

This is a medium-sized species with the holotype having a length of 45.0mm and and a height of 35.0mm. It has a subquadrate outline, compressed form, and there are again signs that the right valve slightly overlaps the left. A low umbonal ridge is more visible on the left valve and there are indications of a chondrophore buttressed with a thin clavicle ([199], p. 1525 & fig. 10- F,G). Although assigned to the genus *Periploma* Schumacher, 1817 by both Zinsmeister [199]) and Stilwell & Zinsmeister [18], Beu ([1], p. 216) has pointed out a much closer match of this species to *Thracia* Sowerby, 1823. The right valve does indeed appear to be larger than the left but knowledge of the chondrophore is still incomplete.

**Additional references**

(References cited in the main paper are not repeated here)

1. Zinsmeister WJ (1983) New Late Paleocene molluscs from the Simi Hills, Venture County, California. J Paleont, 57, 1282-1303.
2. Saul LR (1988a) New Late Cretaceous and Early Tertiary Perissityidae (Gastropoda) from the Pacific Slope of North America. Contrib Sci Nat Hist Mus LA County, 400, 1-25.
3. Griffin M, Hünicken MA (1994) Late Cretaceous – Early Tertiary gastropods from southwestern Patagonia, Argentina. J Paleont, 68, 257-274.
4. Gabb WM (1869) Cretaceous and Tertiary fossils. Cal geol Surv, Paleontol, 2, 1-299.
5. Stewart RB (1927) Gabb’s California fossil type gastropods. Proc Acad Nat Sci Phil, 78, 287-447.
6. Finlay HJ, Marwick J (1937) The Wangaloan and associated molluscan faunas of Kaitangata-Green Island subdivision. Part I – The Wangaloan fauna. NZ Geol Surv Pal Bull, 15, 140p.
7. Wenz W (1938-44) Gastropoda. Band 6, Teil I. Allgemeiner Teil und Prosobranchia. Berli-Zehlendorf.
8. Popenoe WP, Saul LR (1987) Evolution and classification of the Late Cretaceous – Early Tertiary gastropod *Perissitys*. Contrib Sci Nat Hist Mus LA County, 380, 1-37.
9. Saul LR (1988b) Latest Cretaceous and Early Tertiary Tudiclidae and Melongenidae (Gastropoda) from the Pacific Slope of North America. J Paleont, 62, 880-889.
10. Squires, RL (2011) Northeast Pacific Cretaceous record of *Pyropsis* (Neogastropoda: Pyropsidae) and paleobiogeography of the genus. J Paleont, 85, 1199-1215.
11. Del Rio CJ (2012) A new Early Danian gastropod assemblage from northern Patagonia, Rio Negro province, Argentina. J Paleont, 86, 1002-1016.
12. Bandel K, Dockery DT III (2001) The Sarganidae (Pyrifusoidea, Latrogastropoda), their taxonomy and paleobiogeography. J Czech geol Surv, 46, 335-351.
13. Beu AG, Raine JI (2009) Revised descriptions of New Zealand Cenozoic Mollusca from Beu and Maxwell (1990). GNS Science Misc Ser, 27.
14. Sohl NF (1964) Neogastropoda, Opisthobranchia, and Basommatophora from the Ripley, Owl Creek and Prairie Bluff formations. US geol Surv Prof Pap, 331-B, 153-333
15. Wilckens O (1910) Die Anneliden, Bivalven und Gastropoden der antarktischen Kreideformation. Wiss Ergeb Schwed Südpolarexpedition 1901-1903, 3 (12), 1-132.
16. Snyder MA (2003) Catalogue of the marine gastropod family Fasciolariidae. Acad Nat Sci Philadel Spec Pub, 21, 1-328.
17. Meek FB, Hayden FV (1856) Descriptions of new species of Gastropoda from the Cretaceous formations of Nebraska Terr. Acad Nat Sci Phil Proc, 8, 63-69.
18. Erickson JM (1974) Revision of the Gastropoda of the Fox Hills Formation, Upper Cretaceous (Maestrichtian) of North Dakota. Bull Am Paleont, 66, 130-253.
19. Stoliczka F (1867) The Gastropoda of the Cretaceous rocks of southern India, vols 1-4. Mem geol Surv India Pal Indica, 498pp.
20. Sohl NF (1967) Upper Cretaceous gastropods from the Pierre Shale at Red Bird, Wyoming. US geol Surv Prof Pap, 393-B, 46p.
21. Kollman HA, Peel JS (1983) Paleocene gastropods from Nûgssuaq, West Greenland. Bull Grøn Geol Undersøgelse, 146, 115p.
22. Wilckens O (1907) Die lamellibranchiaten gastropoden u.s.w. der oberen Kreide Südpatagoniens. Ber Nat Gesell Freiburg, 15, 97-166.
23. Stilwell JD (2000) Eocene Mollusca (Bivalvia, Gastropoda and Scaphopoda) from McMurdo Sound: Systematics and paleoecologic significance. Ant Res Ser, 76, 261-320.
24. Dell RK (1990) Antarctic Mollusca. Bull R Soc NZ, 27, 311p.
25. White CA (1890) On certain Mesozoic fossils from the islands of St. Paul’s and St. Peter’s in the Straits of Magellan. Proc US Nat Mus, 13, 13-14.
26. Pirrie D, Crame JA, Lomas SA, Riding JB (1997) Late Cretaceous stratigraphy of the Admiralty Sound region, James Ross Basin, Antarctica. Cret Res, 18, 109-137.
27. Weller S (1903) The Stokes Collection of Antarctic fossils, J Geol, 11, 413-419.
28. Dall WH (1890) Synopsis of the Lucinacea and of the American species. Proc US Nat Mus, 23, 779-833.
29. Zelaya DG (2005) The bivalves from the Scotia Arc islands: species richness and faunistic affinities. Scientia Marina, 69, 113-122.
30. Von Martens E (1878) Einige conchylien aus den kälteren Meeresgegenden der südlichen Erdhafte. Zitzber Ges Fremnde Naturforsch Berlin, 20-26.
31. Zinsmeister WJ (1976) A new genus and species of the family Struthiolariidae, *Antarctodarwinella ellioti*, from Seymour Island, Antarctica. Ohio J Sci, 76, 111-114.
32. Powell AWB (1951) Antarctic and Subantarctic Mollusca: Pelecypoda and Gastropoda. Discov Rep, 26, 47-196.
33. Beu AG, Maxwell PA (1990) Cenozoic Mollusca of New Zealand. NZ Geol Surv Pal Bull, 58, 518p.
34. Marshall P (1917) The Wangaloa beds. Trans NZ Inst, 49, 450-460.
35. Stilwell JD (1993) New Early Paleocene Mollusca from the Wangaloa Formation of South Island, New Zealand. J Paleont 67, 360-369.
36. Furque G, Camacho HH (1949) El Cretácico superior de la costa atlántica de Tierra del Fuego. Rev Asoc Geol Arg, 4, 263-298.
37. Camacho HH (1949) La faunula Cretácica del Htio XIX (Tierra del Fuego). Rev Asoc Geol Arg, 4, 249-254.
38. Bandel K, Stinnesbeck W (2000) Gastropods of the Quiriquina Formation (Maastrichtian) in central Chile: Paleobiogeographic relationships and the description of a few new taxa. Zbl Geol Paläont Teil I, 1999, 7/8, 757-788.
39. Darragh TA (1997) Gastropoda, Scaphopoda, Cephalopoda and new Bivalvia of the Paleocene Pebble Point Formation, Victoria, Australia. Proc R Soc Vic, 109, 57-108.
40. Engl W (2012) Shells of Antarctica. Conch Books, Hackenheim, 402p.
41. Harasewych MG, Kantor YI (2004) The deep-sea Buccinoidea (Gastropoda, Neogastropoda) of the Scotia Sea and adjacent abyssal plains and trenches. The Nautilus, 118, 1-42.
42. Kiel S (2001) Taxonomy and biogeography of Late Cretaceous Gastropoda. Dissertation, Hamburg, 162p.
43. Nielsen SN, Frassinetti D (2007) The Neogene Volutidae (Gastropoda: Neogastropoda) from the Pacific Coast of Chile. J Paleont, 81, 82-102.
44. Stilwell JD (2005) A new species of *Calliotropis* (Mollusca: Gastropoda: Vetigastropoda: Trochidae: Eucyclinae) from the Eocene of Antarctica. Moll Res, 25, 9-13.
45. Wilckens O (1911) Die mollusken der antarktischen Tertiarformation. Wiss Ergeb Schwed Südpolarexpedition 1901-1903, 3 (13), 1-62.
46. Steinmann G, Wilckens O (1908) Kreide- und Tertiärfossilien aus der Magellansländern. K Sven Vetensk Stockh Ark Zool, 4, 1-118.
47. Finlay HJ (1930) Invalid molluscan names. No 1. Trans NZ Inst, 61, 37-48.
48. Harasewych MG, Kantor YuI (1999) A revision of the Antarctic genus *Chlanidota* (Gastropoda: Neogastropoda: Buccinulidae). Proc Biol Soc Washington, 112, 253-302.
49. Bouchet P, Kantor YuI, Sysoev A, Puillandre N (2011). A new operational classification of the Conoidea (Gastropoda). J Moll Stud, 77, 273-308.
50. Merle D, Garrigues B, Pointier J-P (2011) Fossil and Recent Muricidae of the world. Part Muricinae. Conch Books, Hackenheim, 652p.
51. Weaver CS, DuPont JE (1970) Living volutes. A monograph of the Recent Volutidae of the world. Delaware Mus Nat Hist Monogr, Ser 1.
52. Leal JH, Harasewych MG (2005) *Tractolira delli*, a new Volutidae (Mollusca: Gastropoda: Neogastropoda) from the abyssal plains off Antarctica. Zootaxa, 1071, 39-45.
53. Beu AG (2011) Marine Mollusca of isotope stages of the last 2 million years in New Zealand. Part 4. Gastropoda (Ptenoglossa, Neogastropoda, Heterobranchia). J R Soc NZ, 41, 1-153.
54. Powell AWB (1966) The molluscan families Speightiidae and Turridae. Bull Auckl Inst Mus, 5, 1-184.
55. Forcelli DO (2000) Moluscos Magallanicos. Vázquez Mazzini Editores, Buenos Aires, 200p.
56. Kilburn RN (1988) Turridae (Mollusca: Gastropoda) of southern Africa and Mozambique. Part 4. Subfamilies Drilliinae, Crassispirinae and Strictispirinae. Ann Natal Mus, 29, 167-320.
57. Zinsmeister WJ (1984) Late Eocene bivalves (Mollusca) from the La Meseta Formation, collected during the 1974-75 joint Argentine-American expedition to Seymour Island, Antarctic Peninsula. J Paleont, 58, 1497-1527.

**Geographical coordinates for all BAS localities on Seymour Island mentioned in the text**

Seymour Island: 64° 17’S; 56° 45’W (BAT Gazetteer)

DJ.77: 64° 13’ 07’’S; 56° 36’ 47’’W

DJ. 78: 64° 13’S; 56° 37’ 25’’W

DJ. 79: 64° 13’ 20’’S; 56° 37’ 25’’W

DJ. 80: 64° 13’ 26’’S; 56° 37’ 07’’W

DJ. 81: 64° 13’ 46’’S; 56° 37’ 47’’W

DJ. 952: base - 64° 16’ 15’’S; 56° 45’ 17’’W; top - 64° 16’ 11’’S; 56° 44’ 38’’W; section transferred laterally through - 64° 16’ 22’’S; 56° 44’ 36’’W

DJ.953: base - 64° 16’ 45’’S; 56° 44’ 45’’W; top - 64° 16’ 52’’S; 56° 43’ 20’’W

DJ. 957: base - 64° 16’ 03’’S; 56° 46’ 07’’W; top - 64° 16’ 15’’S; 56° 45’ 17’’W

DJ. 959: base - 64° 16’S; 56° 46’ 40’’W; top - 64° 16’ 03’’S; 56° 46’ 07’’W

D9. 205: 64° 16.286’S; 56° 43.024’W

D9. 207: 64° 16.019’S; 56° 42.453’W

D9. 208: 64° 15.782’S; 56° 42.493’W

D9.209: base - 64° 15.213’S; 56° 42.281’W; top - 64° 16.308’S; 56° 41.232’W

D9.210: base - 64° 16.272’S; 56° 41.315’W; top - 64° 16.285’S; 56° 40.888’W

D9. 211: base - 64° 15.821’S; 56° 41.520’W; top - 64° 15.861’S; 56° 41.382’W

D9. 212: 64° 15.262’S; 56° 41.530’W

D9.213: 64° 15.550’S; 56° 44.341’W

D9.214: 64° 14.821’S; 56° 40.929’W
